# Nonlinear Monte Carlo Methods with Polynomial Runtime for Bellman Equations of Discrete Time High-Dimensional Stochastic Optimal Control Problems

**DOI:** 10.1007/s00245-024-10213-7

**Published:** 2025-02-04

**Authors:** Christian Beck, Arnulf Jentzen, Konrad Kleinberg, Thomas Kruse

**Affiliations:** 1 Department of Mathematics, ETH Zurich, Zurich, Switzerland; 2 School of Data Science and Shenzhen Research Institute of Big Data, The Chinese University of Hong Kong, Shenzhen (CUHK-Shenzhen), Shenzhen, China; 3 Applied Mathematics: Institute for Analysis and Numerics, University of Münster, Münster, Germany; 4 Department of Mathematics & Informatics, University of Wuppertal, Wuppertal, Germany

**Keywords:** Markov decision processes, Optimal stopping, Bellman equations, Monte Carlo methods, Multilevel fixed-point approximations

## Abstract

Discrete time *stochastic optimal control* problems and *Markov decision processes* (MDPs), respectively, serve as fundamental models for problems that involve sequential decision making under uncertainty and as such constitute the theoretical foundation of *reinforcement learning*. In this article we study the numerical approximation of MDPs with infinite time horizon, finite control set, and general state spaces. Our set-up in particular covers infinite-horizon optimal stopping problems of discrete time Markov processes. A key tool to solve MDPs are *Bellman equations* which characterize the value functions of the MDPs and determine the optimal control strategies. By combining ideas from the *full-history recursive multilevel Picard approximation method*, which was recently introduced to solve certain nonlinear partial differential equations, and ideas from *Q-learning* we introduce a class of suitable *nonlinear Monte Carlo methods* and prove that the proposed methods do not suffer from the *curse of dimensionality* in the numerical approximation of the solutions of Bellman equations and the associated discrete time stochastic optimal control problems.

## Introduction

*Reinforcement learning* is an active research field in machine learning and has important applications in many areas which involve sequential decision making such as economics, engineering, finance, healthcare, logistics, and robotics (see, e.g., Sutton and Barto [54], Bertsekas [13], and Bertsekas and Tistsiklis [15] for overviews of the field and its application areas). In particular the combination of deep neural networks and reinforcement learning, i.e., deep reinforcement learning, has achieved remarkable success in complex decision-making problems during recent years (see, e.g., Li [41], François-Lavet et al. [24], and Arulkumaran et al. [3] for survey articles). The mathematical foundations of reinforcement learning are provided by the theory of *stochastic optimal control* and, in particular, *Markov decision processes* (MDPs; see, e.g., Bertsekas and Shreve [14], Powell [45], and Puterman [46]).

The basis of many reinforcement learning algorithms such as temporal difference learning (see, e.g., Sutton [53]), *Q*-learning (see, e.g., Watkins [57] and Watkins and Dayan [58]), and the SARSA algorithm (see, e.g., Rummery and Niranjan [48]) is formed by the stochastic dynamic programming principle. It reduces the problem of making the optimal decision at a given state to solving a particular functional equation – the so-called *Bellman equation*. The approximative solution of Bellman equations in high dimensional state spaces is a notoriously difficult challenge due to the *curse of dimensionality* (cf., e.g., Bellman [11], Novak and Woźniakowski [44, Chapter 1], and Novak and Ritter [43]) which has been shown to hold for all deterministic numerical approximation methods (cf., e.g., Chow and Tsitsiklis [19, 20]).

In this work we consider randomized numerical approximation methods. More specifically, we introduce *nonlinear Monte Carlo methods* for MDPs with infinite time horizon and finite control set that are polynomially tractable in the sense that the computational effort of the algorithm to approximatively compute the solution of the Bellman equation grows at most polynomially in the reciprocal $1/\epsilon$ of the prescribed approximation accuracy $\epsilon \in (0,1]$ and the dimension $d\in {\mathbb {N}}=\{1,2,3,\ldots \}$ of the underlying state space. In particular, the proposed methods do not suffer from the curse of dimensionality in the numerical approximation of solutions of Bellman equations.

More formally, we consider MDPs that are specified by a measurable space $({\mathbb {X}},{\mathcal {X}})$ (typically $({\mathbb {R}}^d,{\mathcal {B}}({\mathbb {R}}^d))$ with $d\in {\mathbb {N}}$ but our framework also covers discrete state spaces), a finite control set *A*, a discount factor $\delta \in (0,1)$, transition kernels $\kappa _a:{\mathbb {X}} \times {\mathcal {X}} \rightarrow [0,1]$, $a\in A$, and a measurable and bounded reward function $g:{\mathbb {X}}\times A \rightarrow {\mathbb {R}}$. The set of strategies $\mathcal A$ consists of all measurable functions $\alpha :{\mathbb {X}}\rightarrow A$ (under appropriate assumptions the restriction to such non-randomized, stationary strategies is without loss of generality, see, e.g., [14, Chapter 9]). Every $\alpha \in {\mathcal {A}}$ defines a Markov process $X^\alpha =(X^\alpha _k)_{k\in {\mathbb {N}}_0}$ on a filtered probability space $(\Omega , {\mathcal {F}}, ({\mathbb {F}}_k)_{k\in {\mathbb {N}}_0}, {\mathbb {P}})$ with state space $({\mathbb {X}},{\mathcal {X}})$ with the property that for all $k \in {\mathbb {N}}_0$, $B \in {\mathcal {X}}$ it holds ${\mathbb {P}}$-a.s. that ${\mathbb {P}}[X^\alpha _{k+1}\in B|{\mathbb {F}}_k]=\kappa _{\alpha (X^\alpha _k)}(X^\alpha _k, B)$. The expected gain of a strategy $\alpha \in {\mathcal {A}}$ when starting in $x\in {\mathbb {X}}$ is

1 $$\begin{aligned} J(x,\alpha )={\mathbb {E}} \!\left[ \sum _{k=0}^\infty \delta ^k g(X^\alpha _k,\alpha (X^\alpha _k))\Big |X^\alpha _0=x\right] . \end{aligned}$$

The value function $v:{\mathbb {X}} \rightarrow {\mathbb {R}}$ is given by $v(x)=\sup _{\alpha \in {\mathcal {A}}}J(x,\alpha )$, $x\in {\mathbb {X}}$. The dynamic programming principle ensures that under appropriate conditions *v* satisfies for all $x\in {\mathbb {X}}$ that

2 $$\begin{aligned} v(x)=\max _{a\in A}\left\{ g(x,a)+\delta \, \mathbb E[v(X^{x,a})]\right\} , \end{aligned}$$

where it holds for all $x\in {\mathbb {X}}$, $a\in A$ that the random variable $X^{x,a}:\Omega \rightarrow {\mathbb {X}}$ has distribution $\kappa _a(x,\cdot )$ (see, e.g., [14, Chapter 9] for a proof). An important subclass of these MDPs is given by optimal stopping problems in discrete time with infinite time horizon. For such problems the control set *A* consists of the two elements “stop” and “continue” and once the control “stop” is taken the Markov process jumps to a hold state from which it cannot leave and generates no further gains.

There is a large number of numerical approximation methods for Bellman equations of the form (2) that have been proposed and analyzed in the scientific literature. Deterministic numerical approximation methods for Bellman equations suffer in general from the curse of dimensionality. Indeed, Chow and Tsitsiklis [19, 20] show for all $d\in {\mathbb {N}}$ that in the case where the state space ${\mathbb {X}}$ is given by the *d*-dimensional unit cube $[0,1]^d$ deterministic numerical approximation methods need at least $O(\epsilon ^{-2d})$ computational operations to approximate the solution of the Bellman equation with precision $\epsilon \in (0,1]$. Rust [50] shows that under certain assumptions it is possible to overcome this curse of dimensionality by allowing for randomized algorithms. Rust’s method consists of randomly sampling grid points in the state space and performing value iteration on this stochastic grid. The method is model-based as it requires explicit knowledge of the transition density. In the companion paper [49] Rust points out that convergence of his method might break down in situations where the transition density has spikes. Also Kristensen et al. [40] report a dramatic rise in the method’s variance as the state dimension increases. This is theoretically confirmed by Bray [16] who proves that Rust’s method only overcomes the curse of dimensionality in the special case where the MDP is equivalent to an MDP where all but a vanishingly small fraction of state variables behave like history-independent uniform random variables. Variants of Rust’s method for optimal stopping problems are designed by Broadie and Glasserman in [17] and [18]. The literature on optimal stopping comprises a variety of further randomized algorithms. We refer, for example, to [42, 55], and [56] for regression-based algorithms for optimal stopping problems, we refer, for example, to [2, 21, 28, 47], and [12] for duality-based algorithms for optimal stopping problems, and we refer, for example to [9, 10], and [27] for deep learning-based algorithms for optimal stopping problems.

There is a substantial body of research on sampling methods for MDPs in the literature of reinforcement learning. Most closely related to our work are the articles on reinforcement learning with a generative model (i.e., a simulator) that is capable of producing independent realizations of one-step transitions $X^{x,a}$ for arbitrary actions $a\in A$ and states $x\in {\mathbb {X}}$ (see, e.g., [1, 4, 37, 38, 51, 52]). Much of this literature focuses on MDPs with finite state spaces. In contrast, our paper examines MDPs on general state spaces, including ${\mathbb {R}}^d$, $d\in {\mathbb {N}}$, and we are particularly interested in high-dimensional problems.

More specifically, in this paper we introduce nonlinear Monte Carlo algorithms that overcome the curse of dimensionality in the approximation of Bellman equations, that require only very weak regularity assumptions and that are model-free in the sense that they do not use explicit knowledge of the transition kernel $\kappa$ but only need access to a generative model. Our approach combines ideas from *Q-learning* and the recently introduced *full-history recursive multilevel Picard* (MLP) approximations which have been proven to overcome the curse of dimensionality in the numerical approximation of certain semilinear partial differential equations (PDEs) (see, e.g., [5–8, 22, 23, 26, 31–35]). *Q*-learning is based on the idea to switch the order of expectation and maximization in (2). Formally, the *Q*-function satisfies for all $x\in {\mathbb {X}}$, $a\in A$ that $Q(x,a)=g(x,a)+\delta \, {\mathbb {E}}[v(X^{x,a})]$. This together with (2) implies for all $x \in {\mathbb {X}}$ that $v(x)=\max _{a\in A}Q(x,a)$ and, hence, that for all $x \in {\mathbb {X}}$, $a \in A$ it holds that

3 $$\begin{aligned} Q(x,a)=g(x,a)+\delta \, {\mathbb {E}}\!\left[ \max _{b\in A}Q(X^{x,a},b)\right] . \end{aligned}$$

Under appropriate conditions one can show that *Q* is the unique solution of this fixed-point equation and that the sequence of fixed-point iterates $Q_n:{\mathbb {X}} \times A \rightarrow {\mathbb {R}}$, $n\in {\mathbb {N}}_0$, which satisfies for all $n\in {\mathbb {N}}$, $x\in {\mathbb {X}}$, $a\in A$ that $Q_0(x,a)= g(x,a)$ and

4 $$\begin{aligned} Q_{n}(x,a)=g(x,a)+\delta \, {\mathbb {E}}\!\left[ \max _{b\in A}Q_{n-1}(X^{x,a},b)\right] \end{aligned}$$

converges to *Q*. We next employ a central idea of MLP approximations and decompose the iterates into multilevels to obtain for all $n\in {\mathbb {N}}_0$, $x\in {\mathbb {X}}$, $a\in A$ that

5 $$\begin{aligned} \begin{aligned} Q_{n}(x,a)&=g(x,a)+\delta \left[ \sum _{l=0}^{n-1} {\mathbb {E}}\!\left[ \!\left( \!\max _{b\in A}Q_{l}(X^{x,a},b)\!\right) - \mathbb {1}_{{\mathbb {N}}}(l)\! \left( \!\max _{b\in A}Q_{l-1}(X^{x,a},b)\!\right) \! \right] \right] . \end{aligned} \end{aligned}$$

In this telescope expansion, we apply a fundamental idea of Heinrich [29, 30] and Giles [25] and approximate the expected values by Monte Carlo averages with different degrees of accuracy at different levels $l\in \{1,\ldots ,n\}$. The convergence of $(Q_l)_{l\in {\mathbb {N}}}$ ensures that for large $l\in \{1,\ldots ,n\}$ the difference between $Q_{l}$ and $Q_{l-1}$ is small and hence we use for large $l\in \{1,\ldots ,n\}$ less Monte Carlo samples to approximate the expected value ${\mathbb {E}}\left[ \left( \max _{b\in A}Q_{l}(X^{x,a},b)\right) -\left( \max _{b\in A}Q_{l-1}(X^{x,a},b)\right) \right]$ than for small $l\in \{1,\ldots ,n\}$. More specifically, we fix $M\in {\mathbb {N}}$ and use $M^{n-l}$ independent Monte Carlo samples to approximate the expected value at level $l\in \{1,\ldots ,n\}$ which leads to the full-history recursive multilevel fixed-point (MLFP) approximation scheme in (7) below.

To briefly sketch the contribution of this article within this introductory section, we now present in the following result, Theorem 1.1 below, a special case of Theorem 4.1, the main result of this article. Below Theorem 1.1 we explain in words the statement of Theorem 1.1 as well as the mathematical objects appearing in Theorem 1.1.

### Theorem 1.1

Let *A* be a nonempty set, for every $d\in {\mathbb {N}}$ let $g_d:{\mathbb {R}}^d \times A \rightarrow {\mathbb {R}}$ be $({\mathcal {B}}({\mathbb {R}}^d) \otimes \text{2 }^{A}) / {\mathcal {B}}({\mathbb {R}})$-measurable, assume

6 $$\begin{aligned} \sup _{d\in {\mathbb {N}}} \sup _{x \in {\mathbb {R}}^d}\sup _{ a\in A} |g_d(x, a)| < \infty , \end{aligned}$$

let $(\Omega , {\mathcal {F}}, {\mathbb {P}})$ be a probability space, let $\Theta = \cup _{n \in {\mathbb {N}}} {\mathbb {Z}}^n$, for^1^ every $d\in {\mathbb {N}}$ let ${\mathbb {F}}_d^\theta \subseteq {\mathcal {F}}$, $\theta \in \Theta$, be independent sub-sigma-algebras of ${\mathcal {F}}$, for every $d\in {\mathbb {N}}$ let $X_d^{\theta } = (X_d^{\theta , x, a} (\omega ) )_{ (x, a, \omega ) \in {\mathbb {R}}^d\times A\times \Omega } :{\mathbb {R}}^d\times A \times \Omega \rightarrow {\mathbb {R}}^d$, $\theta \in \Theta ,$ be i.i.d. random fields which satisfy for all $d\in {\mathbb {N}}$, $\theta \in \Theta$ that $X_d^\theta$ is $( {\mathcal {B}}({\mathbb {R}}^d) \otimes \text{2 }^{A} \otimes {\mathbb {F}}_d^\theta ) / {\mathcal {B}}({\mathbb {R}}^d)$-measurable, for every $d\in {\mathbb {N}}$ let $\delta _d\in [0,1)$, ${\mathcal {R}}_d\in [0, \infty )$, let $M \in {\mathbb {N}}\cap [\sup _{d\in {\mathbb {N}}} (4 |A|^2 (1-\delta _d)^{-2}), \infty ]$, for every $d\in {\mathbb {N}}$ let ${\mathcal {Q}}_{d, n}^\theta :{\mathbb {R}}^d\times A \times \Omega \rightarrow {\mathbb {R}}$, $n \in {\mathbb {N}}_0$, $\theta \in \Theta$, satisfy for all $n \in {\mathbb {N}}_0$, $\theta \in \Theta$, $x \in {\mathbb {R}}^d$, $a\in A$ that

7 $$\begin{aligned} {\mathcal {Q}}_{d, n}^{\theta }(x,a)= &   g_d(x,a) + \sum _{l = 0}^{n-1} \frac{\delta _d}{M^{n-l}} \sum _{i = 1}^{M^{n-l}} \left[ \max _{b \in A}\big \{\! {\mathcal {Q}}_{d, l}^{(\theta , l, i)}(X_d^{(\theta , l, i), x,a},b) \! \big \} \nonumber \right. \\  &   \left. -\mathbb {1}_{{\mathbb {N}}}(l) \max _{b \in A} \big \{\! {\mathcal {Q}}_{d, \max \{ l-1, 0 \}}^{(\theta , -l, i)}(X_d^{(\theta , l, i), x,a},b) \!\big \}\right] , \end{aligned}$$

and for every $d\in {\mathbb {N}}$ let ${\mathcal {C}}_{d, n} \in [0, \infty )$, $n \in {\mathbb {N}}_0$, satisfy for all $n \in {\mathbb {N}}_0$ that

8 $$\begin{aligned} {\mathcal {C}}_{d, n} \le \sum _{l = 0}^{n-1} M^{n-l}\big ( {\mathcal {R}}_d+ |A|{\mathcal {C}}_{d, l} + |A|{\mathcal {C}}_{d, \max \{l-1, 0\}} \mathbb {1}_{{\mathbb {N}}}(l)\big ). \end{aligned}$$

Then

(i) it holds for all $d\in {\mathbb {N}}$ that there exists a unique bounded $({\mathcal {B}}({\mathbb {R}}^d)\otimes \text{2 }^{A})/ {\mathcal {B}}({\mathbb {R}})$-measurable $Q_d:{\mathbb {R}}^d\times A \rightarrow {\mathbb {R}}$ which satisfies for all $x \in {\mathbb {R}}^d$, $a \in A$ that
  9 $$\begin{aligned} Q_d(x,a) = g_d(x, a) + \delta _d\, {\mathbb {E}}\! \left[ \max _{b \in A} Q_d(X_d^{0,x,a}, b) \right] \end{aligned}$$
  and
(ii) there exist $N :(0, 1] \rightarrow {\mathbb {N}}$ and $c \in {\mathbb {R}}$ such that for all $d\in {\mathbb {N}}$, $\varepsilon \in (0,1]$ it holds that ${\mathcal {C}}_{d, N_\varepsilon } \le c {\mathcal {R}}_d\varepsilon ^{-c}$ and 10

Theorem 1.1 is an immediate consequence from Corollary 4.2 in Sect. 4 below. Corollary 4.2, in turn, follows from Theorem 4.1, which is the main result of this article. In the following we add some comments on the mathematical objects appearing in Theorem 1.1 above.

In Theorem 1.1 we introduce in (7) a Monte Carlo-type approximation algorithm for a sequence of MDPs indexed by the dimension $d\in {\mathbb {N}}$ of the state space. To formulate the proposed Monte Carlo-type approximation algorithm in (7) we need, roughly speaking, sufficiently many independent random variables which are indexed over a sufficiently large index set. This sufficiently large index set is provided through the set $\Theta = \cup _{n \in {\mathbb {N}}} {\mathbb {Z}}^n$ introduced in Theorem 1.1. The triple $(\Omega , {\mathcal {F}}, {\mathbb {P}})$ in Theorem 1.1 is the probability space on which the random variables are defined. In Theorem 1.1 we consider for every $d\in {\mathbb {N}}$ an MDP with state space $({\mathbb {R}}^d, {\mathcal {B}}({\mathbb {R}}^d))$. We assume that all elements of the sequence of MDPs have a common control set *A*. For every $d\in {\mathbb {N}}$, $x\in {\mathbb {R}}^d$, $a\in A$ the one-step transition of the controlled Markov chain of the MDP with state space $({\mathbb {R}}^d, {\mathcal {B}}({\mathbb {R}}^d))$ is given by the random variable $X_d^{0,x,a}:\Omega \rightarrow {\mathbb {R}}^d$. In the language of MDPs for every $d\in {\mathbb {N}}$, $a\in A$ the transition kernel

11 $$\begin{aligned} \kappa _{d,a}:{\mathbb {R}}^d\times {\mathcal {B}}({\mathbb {R}}^d) \rightarrow [0,1] \end{aligned}$$

is thus determined by the distribution of $X_d^{0,\cdot ,a}$. For every $d\in {\mathbb {N}}$ the function $g_d:{\mathbb {R}}^d\times A \rightarrow {\mathbb {R}}$ introduced in the first line of Theorem 1.1 is the reward function of the MDP with state space $({\mathbb {R}}^d, \mathcal B({\mathbb {R}}^d))$. In Theorem 1.1 we assume that the functions $g_d$, $d\in {\mathbb {N}}$, are uniformly bounded in $d\in {\mathbb {N}}$. For every $d\in {\mathbb {N}}$ the real number $\delta _d\in [0, 1)$ introduced in Theorem 1.1 is the discount factor of the MDP with state space $({\mathbb {R}}^d, {\mathcal {B}}({\mathbb {R}}^d))$.

Item (i) in Theorem 1.1 establishes the essentially well-known result that under the above assumptions for every $d\in {\mathbb {N}}$ the Bellman equation (2) for the *Q*-function associated to the MDP with state space $({\mathbb {R}}^d, {\mathcal {B}}({\mathbb {R}}^d))$ has a unique solution $Q_d:{\mathbb {R}}^d\times A\rightarrow {\mathbb {R}}$.

In (7) in Theorem 1.1 we specify the MLFP approximation scheme which we propose to approximate the solution of the Bellman equation (2). The MLFP approximations ${\mathcal {Q}}^\theta _{d, n}$, $d\in {\mathbb {N}}$, $n\in {\mathbb {N}}_0$, $\theta \in \Theta$, are indexed by the dimension $d\in {\mathbb {N}}$, by the number $n\in {\mathbb {N}}_0$ of fixed-point iterates, and by a parameter $\theta \in \Theta$ which is different for different appearences of MLFP approximations in (7). As random input sources the MLFP approximation scheme proposed in (7) employs the random variables

12 $$\begin{aligned} X_d^{\theta ,x,a}:\Omega \rightarrow {\mathbb {R}}^d \end{aligned}$$

for $\theta \in \Theta \backslash \{0\}$, $d\in {\mathbb {N}}$, $x\in {\mathbb {R}}^d$, $a\in A$. Note that for every $\theta \in \Theta \backslash \{0\}$, $d\in {\mathbb {N}}$, $x\in {\mathbb {R}}^d$, $a\in A$ we have that the random variable $X_d^{\theta ,x,a}:\Omega \rightarrow {\mathbb {R}}^d$ which is used as random input source of the MLFP approximation scheme proposed in (7) and the random variable $X_d^{0,x,a}:\Omega \rightarrow {\mathbb {R}}^d$ which is used to formulate the Bellman equation (2) are identically distributed. The parameter $\theta \in \Theta$ ensures that different appearances of MLFP approximations in (7) are independent and this ensures that (7) can be implemented with recursive function calls. The natural number $M \in {\mathbb {N}}\cap [\sup _{d\in {\mathbb {N}}} (4 |A|^2 (1-\delta _d)^{-2}), \infty ]$ in Theorem 1.1 determines the number of Monte Carlo samples used in the definition of the MLFP approximation ${\mathcal {Q}}^\theta _{d,n}$, $d\in {\mathbb {N}}$, $n\in {\mathbb {N}}_0$, $\theta \in \Theta$, in (7). The assumption that the natural number *M* is an element of the set ${\mathbb {N}}\cap [\sup _{d\in {\mathbb {N}}} (4 |A|^2 (1-\delta _d)^{-2}), \infty ]$ ensures that

13 $$\begin{aligned} \sup _{d\in {\mathbb {N}}} (4 |A|^2 (1-\delta _d)^{-2}) < \infty . \end{aligned}$$

This implies that the control set *A* is finite and that the sequence $(\delta _d)_{d\in {\mathbb {N}}}\subseteq [0,1)$ is bounded away from 1 in the sense that $\sup _{d\in {\mathbb {N}}} \delta _d< 1$. Note that Theorem 1.1 also covers the case where for each dimension $d\in {\mathbb {N}}$ there is an individual control set ${\mathcal {A}}_d$ with the property that $\sup _{d\in {\mathbb {N}}} |{\mathcal {A}}_d| < \infty$ by considering $A = \cup _{d\in {\mathbb {N}}} {\mathcal {A}}_d$.

For every $d\in {\mathbb {N}}$, $x \in {\mathbb {R}}^d$, $\theta \in \Theta$ the nonnegative real number ${\mathcal {R}}_d\in [0, \infty )$ in Theorem 1.1 is understood as an upper bound for the computational cost to compute one realization of the random variable $(X^{\theta ,x, a}_d)_{a \in A}$. The real numbers ${\mathcal {C}}_{d,n}\in [0,\infty )$, $d\in {\mathbb {N}}$, $n\in {\mathbb {N}}_0$, in (8) in Theorem 1.1 model the computational costs of the MLFP approximation scheme in (7) (i.e., the sample complexity). More specifically, for every $d\in {\mathbb {N}}$, $n \in {\mathbb {N}}_0$ we have that the real number ${\mathcal {C}}_{d, n}\in [0,\infty )$ represents an upper bound for the number of realizations of the random variables $(X^{\theta , x, a}_d)_{a \in A}$ for $(x, \theta ) \in {\mathbb {R}}^d\times \Theta$ required to compute one realization of the random variable $({\mathcal {Q}}^0_{d, n}(0, a))_{a \in A}$.

Item (ii) in Theorem 1.1 proves that the solutions of the Bellman equations in (2) can be approximated in the $L^2$-sense by means of the MLFP approximation scheme in (7) with a computational cost which grows at most polynomially in the reciprocal $1/\epsilon$ of the prescribed approximation accuracy $\epsilon \in (0,1]$ and linearly in the computational cost ${\mathcal {R}}_d$ to compute one realization of any of the random variables $X^{\theta ,x}_d:\Omega \rightarrow ({\mathbb {R}}^d)^A$, $\theta \in \Theta$, $x\in {\mathbb {R}}^d$, where $d\in {\mathbb {N}}$ is the dimension of the state space of the associated MDP. In particular, if the computational cost to compute one realization of any of the random variables $X^{\theta ,x}_d:\Omega \rightarrow ({\mathbb {R}}^d)^A$, $\theta \in \Theta$, $x\in {\mathbb {R}}^d$, grows at most polynomially in the dimension $d\in {\mathbb {N}}$ of the state space of the associated MDP (as it is often the case in practical applications), then the MLFP approximation scheme in (7) overcomes the curse of dimensionality for the approximation of the solutions of the Bellman equations in (2). However, we would like to point out that the constant $c \in {\mathbb {R}}$ appearing in item (ii) in Theorem 1.1 may become arbitrary large if $\sup _{d\in {\mathbb {N}}}\delta _d$ is close to 1. Thus the computational cost of the nonlinear Monte Carlo methods in (7) may become impractical even so the methods in (7) provably overcome the curse of dimensionality.

In the following we also add some comments on generalizations and variants of Theorem 1.1 presented in this article. While Theorem 1.1 considers a sequence of MDPs indexed by the dimension $d\in {\mathbb {N}}$ of the Euclidean state spaces $({\mathbb {R}}^d, {\mathcal {B}}({\mathbb {R}}^d))$, Corollary 4.2 considers a family of MDPs with general index set ${\mathfrak {D}}$ and general measurable state spaces $({\mathbb {X}}_d, {\mathcal {X}}_d)$, $d\in {\mathfrak {D}}$. While Theorem 1.1 considers MDPs with bounded reward functions, Corollary 4.2 allows for unbounded reward functions. While Theorem 1.1 and Corollary 4.2 consider Bellman equations of MDPs, Theorem 4.1 considers more general functional fixed-point equations (we refer to (91) in Theorem 4.1 for details). Corollary 4.3 proves that a variant of the MLFP approximation scheme (see (119) for details) overcomes the curse of dimensionality for the approximation of the solutions of Bellman equations for optimal stopping problems (see (121) for details).

The remainder of this article is organized as follows. In Sect. 2 below we establish existence, uniqueness, and integrability properties for solutions of functional fixed-point equations. In Sect. 3 below we introduce MLFP approximations for solutions of functional fixed-point equations, we study measurability, distributional, and integrability properties for the introduced MLFP approximations and we establish recursive and subsequently non-recursive upper bounds for the $L^2$-distances between the exact solutions of the considered functional fixed-point equations and the proposed MLFP approximations. In Sect. 4 we combine the existence, uniqueness, and regularity properties for solutions of functional fixed-point equations, which we have established in Sect. 2, with the error analysis for MLFP approximations for functional fixed-point equations, which we have established in Sect. 3, to obtain a computational complexity analysis for MLFP approximations for functional fixed-point equations and for Bellman equations of MDPs and optimal stopping problems.

## Existence and Uniqueness of Solutions of Functional Fixed-Point Equations

In this section we provide in Lemma 2.2 an elementary existence and uniqueness result for functional fixed-point equations formulated using stochastic kernels. In Corollary 2.3 we specialize Lemma 2.2 for functional fixed-point equations formulated using random variables. Lemma 2.4 establishes a priori estimates for solutions of functional fixed-point equations. In the special case where the functional fixed-point equation takes the form of a Bellman equation associated to an optimal stochastic control problem and the weight function ${\mathfrak {w}}$ is constant equal to one the results of this section are essentially well-known (see, e.g., [46, Theorem 6.2.5]).

### Definition 2.1

Let $({\mathbb {X}}, {\mathcal {X}})$ and $({\mathbb {Y}}, {\mathcal {Y}})$ be nonempty measurable spaces and let $\kappa :{\mathbb {X}}\times {\mathcal {Y}} \rightarrow [0,1]$ satisfy for all $M \in {\mathcal {Y}}$ that ${\mathbb {X}}\ni x \mapsto \kappa (x,M) \in [0,1]$ is ${\mathcal {X}}/{\mathcal {B}}( [0,1])$-measurable and for all $x \in {\mathbb {X}}$ that ${\mathcal {Y}} \ni M \mapsto \kappa (x,M) \in [0,1]$ is a probability measure on $({\mathbb {Y}}, {\mathcal {Y}})$. Then we say that $\kappa$ is a stochastic kernel from $({\mathbb {X}}, {\mathcal {X}})$ to $({\mathbb {Y}}, {\mathcal {Y}})$.

### Lemma 2.2

Let $c,L \in [0,\infty )$ with $cL < 1$, let $({\mathbb {X}}, {\mathcal {X}})$ be a nonempty measurable space, let *A* be a nonempty at most countable set, for every $a \in A$ let $\kappa _a :{\mathbb {X}}\times {\mathcal {X}} \rightarrow [0,1]$ be a stochastic kernel from $({\mathbb {X}}, {\mathcal {X}})$ to $({\mathbb {X}}, {\mathcal {X}})$, let $\widetilde{{\mathcal {A}}}= \bigotimes _{a \in A} {\mathcal {B}}({\mathbb {R}})$, let $f :{\mathbb {X}}\times {\mathbb {R}}^A \rightarrow {\mathbb {R}}$ be $({\mathcal {X}} \otimes \widetilde{{\mathcal {A}}})/{\mathcal {B}}({\mathbb {R}})$-measurable, let ${\mathfrak {w}}:{\mathbb {X}}\rightarrow (0,\infty )^A$ be ${\mathcal {X}}/\bigotimes _{a \in A}{\mathcal {B}}\big ((0, \infty )\big )$-measurable, let

14 $$\begin{aligned} \begin{aligned} {\mathfrak {W}}= \bigg \{\! (u :{\mathbb {X}}\rightarrow {\mathbb {R}}^A) : u \text{ is } {\mathcal {X}}/\widetilde{{\mathcal {A}}}\text{-measurable, } \sup _{(x,a) \in {\mathbb {X}}\times A} \big [|\big ({\mathfrak {w}}(x)\big )(a)|^{-1} |\big (u(x)\big )(a)| \big ] < \infty \! \bigg \}, \end{aligned} \end{aligned}$$

and assume for all $x \in {\mathbb {X}}$, $a \in A$, $r,s \in {\mathbb {R}}^A$ that $|f(x,r) - f(x,s)| \le L \sup _{b \in A} |r(b) - s(b) |$, $\int _{\mathbb {X}}\sup _{b \in A} \big ({\mathfrak {w}}(y)\big )(b)\kappa _a(x,dy) \le c \big ({\mathfrak {w}}(x)\big )(a)$, and $\sup _{(t,b) \in {\mathbb {X}}\times A} [|\big ({\mathfrak {w}}(t)\big )(b)|^{-1} \int _{\mathbb {X}}|f(y,0)|\kappa _b(t,dy)] < \infty$. Then there exists a unique $v \in {\mathfrak {W}}$ which satisfies for all $x \in {\mathbb {X}}$, $a \in A$ that

15 $$\begin{aligned} \int _{\mathbb {X}}|f(y,v(y))| \kappa _a(x,dy) < \infty \qquad \text {and} \qquad \big (v(x)\big )(a) = \int _{\mathbb {X}}f(y,v(y))\kappa _a(x,dy). \end{aligned}$$

### Proof of Lemma 2.2

Let $\left\| \cdot \right\| :{\mathfrak {W}}\rightarrow [0,\infty )$ satisfy for all $u \in {\mathfrak {W}}$ that $\Vert u\Vert = \sup _{(x,a) \in {\mathbb {X}}\times A} \frac{|\left( u(x)\right) (a)|}{|\left( {\mathfrak {w}}(x)\right) (a)|}$. Note that $\left\| \cdot \right\|$ is a norm on ${\mathfrak {W}}$ and $({\mathfrak {W}}, \left\| \cdot \right\| )$ is a Banach space. The assumption that for all $x \in {\mathbb {X}}$, $r,s \in {\mathbb {R}}^A$ it holds that $|f(x,r) - f(x,s)| \le L \sup _{a \in A} |r(a) - s(a)|$ and the assumption that for all $x \in {\mathbb {X}}$, $a \in A$ it holds that $\int _{\mathbb {X}}\sup _{b \in A} \big ({\mathfrak {w}}(y)\big )(b) \kappa _a(x,dy) \le c \big ({\mathfrak {w}}(x)\big )(a)$, yield that for all $u \in {\mathfrak {W}}$, $x \in {\mathbb {X}}$, $a \in A$ it holds that

16 $$\begin{aligned}&\frac{1}{\big ({\mathfrak {w}}(x)\big )(a) } \bigg | \int _{\mathbb {X}}f(y,u(y)) \kappa _a(x,dy) \bigg | \nonumber \\&\quad \le \frac{1}{\big ({\mathfrak {w}}(x)\big )(a)} \int _{\mathbb {X}}|f(y,u(y)) - f(y,0)| \kappa _a(x,dy) \nonumber \\&\quad \quad + \frac{1}{\big ({\mathfrak {w}}(x)\big )(a)} \int _{\mathbb {X}}|f(y,0)| \kappa _a(x,dy)\nonumber \\&\quad \le \frac{L}{\big ({\mathfrak {w}}(x)\big )(a)} \int _{\mathbb {X}}\sup _{b \in A} |\big (u(y)\big )(b)| \kappa _a(x,dy) \nonumber \\&\quad \quad + \frac{1}{\big ({\mathfrak {w}}(x)\big )(a)} \int _{\mathbb {X}}|f(y,0)| \kappa _a(x,dy)\nonumber \\&\quad = \frac{L}{\big ({\mathfrak {w}}(x)\big )(a)} \int _{\mathbb {X}}\sup _{b \in A} \bigg \{ \frac{| \big (u(y)\big )(b)|}{\big ({\mathfrak {w}}(y)\big )(b)}\big ({\mathfrak {w}}(y)\big )(b) \bigg \} \kappa _a(x,dy) \nonumber \\&\quad \quad + \frac{1}{\big ({\mathfrak {w}}(x)\big )(a)} \int _{\mathbb {X}}|f(y,0)| \kappa _a(x,dy)\nonumber \\&\quad \le \frac{L \Vert u \Vert }{\big ({\mathfrak {w}}(x)\big )(a)} \int _{\mathbb {X}}\sup _{b \in A}\big ({\mathfrak {w}}(y)\big )(b) \kappa _a(x,dy) + \frac{1}{\big ({\mathfrak {w}}(x)\big )(a)} \int _{\mathbb {X}}|f(y,0)| \kappa _a(x,dy)\nonumber \\&\quad \le cL \Vert u\Vert + \frac{1}{\big ({\mathfrak {w}}(x)\big )(a)} \int _{\mathbb {X}}|f(y,0)| \kappa _a(x,dy). \end{aligned}$$

The assumption that $\sup _{(x,a) \in {\mathbb {X}}\times A} \big [|\big ({\mathfrak {w}}(x)\big )(a)|^{-1}\int _{\mathbb {X}}\big | f(y,0) \big | \kappa _a(x,dy)\big ] < \infty$ demonstrates that for all $u \in {\mathfrak {W}}$ it holds that

17 $$\begin{aligned}&\sup _{(x,a) \in {\mathbb {X}}\times A} \frac{1}{\big ({\mathfrak {w}}(x)\big )(a) } \bigg | \int _{\mathbb {X}}f(y,u(y)) \kappa _a(x,dy) \bigg |\nonumber \\&\quad \le cL \Vert u \Vert + \sup _{(x,a) \in {\mathbb {X}}\times A} \frac{1}{\big ({\mathfrak {w}}(x)\big )(a)} \int _{\mathbb {X}}|f(y,0)| \kappa _a(x,dy) < \infty . \end{aligned}$$

Next, observe that [39, Lemma 14.20] ensures that for all $u \in {\mathfrak {W}}$ it holds that the map ${\mathbb {X}}\ni x \mapsto \big [ A \ni a \mapsto \int _{\mathbb {X}}f(y,u(y)) \kappa _a(x,dy) \in {\mathbb {R}}\big ] \in {\mathbb {R}}^A$ is ${\mathcal {X}}/\widetilde{{\mathcal {A}}}$-measurable. Let $\Phi :{\mathfrak {W}}\rightarrow {\mathfrak {W}}$ be the function which satisfies for all $u \in {\mathfrak {W}}$, $(x,a) \in {\mathbb {X}}\times A$ that

18 $$\begin{aligned} {[}\Phi (u)](x)(a) = \int _{\mathbb {X}}f(y,u(y))\kappa _a(x,dy). \end{aligned}$$

The assumption that for all $x \in {\mathbb {X}}$, $r,s \in {\mathbb {R}}^A$ it holds that $|f(x,r) - f(x,s)| \le L \sup _{a \in A} |r(a) - s(a)|$, the assumption that $\sup _{(x,a) \in {\mathbb {X}}\times A} \big [|\big ({\mathfrak {w}}(x)\big )(a)|^{-1}\int _{\mathbb {X}}\big | f(y,0) \big | \kappa _a(x,dy)\big ] < \infty$, and the assumption that for all $(x,a) \in {\mathbb {X}}\times A$ it holds that $\int _{\mathbb {X}}\sup _{b \in A}\big ({\mathfrak {w}}(y)\big )(b) \kappa _a(x,dy) \le c \big ({\mathfrak {w}}(x)\big )(a)$, ensure that for all $u,v \in {\mathfrak {W}}, (x,a) \in {\mathbb {X}}\times A$ it holds that

19 $$\begin{aligned}&\frac{1}{ \big ({\mathfrak {w}}(x)\big )(a)}\Big | [\Phi (u)](x)(a) - [\Phi (v)](x)(a) \Big |\nonumber \\&\quad \le \frac{1}{\big ({\mathfrak {w}}(x)\big )(a)} \int _{\mathbb {X}}\big | f(y,u(y)) - f(y,v(y)) \big |\kappa _a(x,dy)\nonumber \\&\quad \le \frac{L}{\big ({\mathfrak {w}}(x)\big )(a)} \int _{\mathbb {X}}\sup _{b \in A} | \big (u(y)\big )(b) - \big (v(y)\big )(b) | \kappa _a(x,dy)\nonumber \\&\quad = \frac{L}{\big ({\mathfrak {w}}(x)\big )(a)} \int _{\mathbb {X}}\sup _{b \in A} \bigg \{ \frac{|\big (u(y)\big )(b) - \big (v(y)\big )(b)|}{\big ({\mathfrak {w}}(y)\big )(b)} \big ({\mathfrak {w}}(y)\big )(b) \bigg \} \kappa _a(x,dy)\nonumber \\&\quad \le \frac{L \Vert u - v \Vert }{\big ({\mathfrak {w}}(x)\big )(a)} \int _{\mathbb {X}}\sup _{b \in A} \big ({\mathfrak {w}}(y)\big )(b)\kappa _a(x,dy)\nonumber \\&\quad \le cL \Vert u - v \Vert . \end{aligned}$$

This in turn proves that for all $u,v \in {\mathfrak {W}}$ it holds that

20 $$\begin{aligned} \big \Vert \Phi (u) - \Phi (v) \big \Vert \le cL\Vert u - v \Vert . \end{aligned}$$

The assumption that $cL < 1$ shows that $\Phi$ is a contraction. Hence Banach’s fixed-point theorem proves that there exists a unique function $v \in {\mathfrak {W}}$ such that $v = \Phi (v)$. The fact that $v \in {\mathfrak {W}}$ ensures that for all $(x,a) \in {\mathbb {X}}\times A$ it holds that $\int _{\mathbb {X}}| f(y,v(y)) | \kappa _a(x,dy) < \infty$. The proof of Lemma 2.2 is thus complete. $\square$

### Corollary 2.3

Let $c,L \in [0, \infty )$ with $cL < 1$, let $({\mathbb {X}}, {\mathcal {X}})$ be a nonempty measurable space, let $(\Omega , {\mathcal {F}}, {\mathbb {P}})$ be a probability space, let *A* be a nonempty at most countable set, let $\widetilde{{\mathcal {A}}}= \bigotimes _{a \in A} {\mathcal {B}}({\mathbb {R}})$, let $\widetilde{{\mathcal {X}}} = \bigotimes _{a \in A} {\mathcal {X}}$, let $X = \big ( X^{x,a}(\omega ) \big )_{x \in {\mathbb {X}},\;a\in A,\; \omega \in \Omega }:{\mathbb {X}}\times \Omega \rightarrow {\mathbb {X}}^A$ be $({\mathcal {X}} \otimes {\mathcal {F}})/\widetilde{{\mathcal {X}}}$-measurable, let $f :{\mathbb {X}}\times {\mathbb {R}}^A \rightarrow {\mathbb {R}}$ be $({\mathcal {X}} \otimes \widetilde{{\mathcal {A}}})/{\mathcal {B}}({\mathbb {R}})$-measurable, let ${\mathfrak {w}}:{\mathbb {X}}\rightarrow (0,\infty )^A$ be ${\mathcal {X}}/\bigotimes _{a \in A}{\mathcal {B}}\big ((0, \infty )\big )$-measurable, let

21 $$\begin{aligned} \begin{aligned}&{\mathfrak {W}}= \bigg \{ \!(u :{\mathbb {X}}\rightarrow {\mathbb {R}}^A) : u \text{ is } {\mathcal {X}}/\widetilde{{\mathcal {A}}}\text{-measurable, } \\  &\sup _{(x,a) \in {\mathbb {X}}\times A} \big [|\big ({\mathfrak {w}}(x)\big )(a)|^{-1} |\big (u(x)\big )(a)| \big ] < \infty \! \bigg \}, \end{aligned} \end{aligned}$$

assume that for all $(x,a) \in {\mathbb {X}}\times A$, $r,s \in {\mathbb {R}}^A$ it holds that $|f(x,r) - f(x,s)| \le L \sup _{b \in A} |r(b) - s(b)|$, ${\mathbb {E}}[\sup _{b \in A} \big ({\mathfrak {w}}(X^{x,a})\big )(b)] \le c \big ({\mathfrak {w}}(x)\big )(a)$, and $\sup _{(y, b) \in {\mathbb {X}}\times A} \big [ | \big ({\mathfrak {w}}(y)\big )(b)|^{-1}{\mathbb {E}}[|f(X^{y,b},0)|] \big ] < \infty$. Then there exists a unique function $v \in {\mathfrak {W}}$ which satisfies for all $(x,a) \in {\mathbb {X}}\times A$ that

22 $$\begin{aligned} {\mathbb {E}}\big [|f(X^{x,a},v(X^{x,a}))|\big ] < \infty \qquad \text {and} \qquad \big (v(x)\big )(a) = {\mathbb {E}}\big [ f(X^{x,a},v(X^{x,a})) \big ]. \end{aligned}$$

### Proof of Corollary 2.3

Let $\kappa _a :{\mathbb {X}}\times {\mathcal {X}} \rightarrow [0,1]$, $a \in A$, satisfy for all $(x,a) \in {\mathbb {X}}\times A$, $M \in {\mathcal {X}}$, that $\kappa _a(x,M) = {\mathbb {P}}[X^{x,a} \in M]$. The fact that *X* is $({\mathcal {X}} \otimes {\mathcal {F}})/\widetilde{{\mathcal {X}}}$-measurable ensures that for all $(x,a) \in {\mathbb {X}}\times A$ it holds that $X^{x,a} :\Omega \rightarrow {\mathbb {X}}$ is ${\mathcal {F}}/{\mathcal {X}}$-measurable. This implies that for all $(x,a) \in {\mathbb {X}}\times A$ it holds that ${\mathcal {X}} \ni M \mapsto \kappa _a(x,M) = {\mathbb {P}}[X^{x,a} \in M] \in [0,1]$ is a probability measure on $({\mathbb {X}},{\mathcal {X}})$. The fact that *X* is $({\mathcal {X}} \otimes {\mathcal {F}})/\widetilde{{\mathcal {X}}}$-measurable and [39, Theorem 14.16] imply that for all $a \in A,$ $M \in {\mathcal {X}}$ it holds that ${\mathbb {X}}\ni x \mapsto \kappa _a (x,M) = {\mathbb {P}}[X^{x,a} \in M]$ is ${\mathcal {X}}/ {\mathcal {B}}([0,1])$-measurable. Hence it holds for all $a \in A$ that $\kappa _a$ is a stochastic kernel form $({\mathbb {X}}, {\mathcal {X}})$ to $({\mathbb {X}}, {\mathcal {X}})$. The assumption that for all $(x,a)\in {\mathbb {X}}\times A$ it holds that ${\mathbb {E}}\big [ \sup _{b \in A} \big ({\mathfrak {w}}(X^{x,a})\big )(b) \big ] \le c \big ({\mathfrak {w}}(x)\big )(a)$ and the assumption that $\sup _{(y,b) \in {\mathbb {X}}\times A} \big [ | \big ({\mathfrak {w}}(y)\big )(b) |^{-1} {\mathbb {E}}[ |f(X^{y,b}, 0 )| ] \big ] < \infty$ prove that for all $(x,a) \in {\mathbb {X}}\times A$ it holds that

23 $$\begin{aligned} \int _{\mathbb {X}}\sup _{b \in A} \big ({\mathfrak {w}}(y)\big )(b) \kappa _a(x,dy)&= \hspace{-0.15cm} \int _{\mathbb {X}}\sup _{b \in A} \big ({\mathfrak {w}}(y)\big )(b) \big ( X^{x,a}({\mathbb {P}}) \big )(dy) \nonumber \\&= {\mathbb {E}}\Big [\!\sup _{b \in A} \big ({\mathfrak {w}}(X^{x,a})\big )(b) \!\Big ]\le c \big ({\mathfrak {w}}(x)\big )(a), \end{aligned}$$

and

24 $$\begin{aligned}&\sup _{(y,b)\in {\mathbb {X}}\times A} \frac{1}{\big ({\mathfrak {w}}(y)\big )(b)} \int _{\mathbb {X}}|f(t,0)| \kappa _b(y,dt)\nonumber \\&\quad = \sup _{(y,b)\in {\mathbb {X}}\times A} \frac{1}{\big ({\mathfrak {w}}(y)\big )(b)} \int _{\mathbb {X}}|f(t,0)|\big ( X^{y,b}({\mathbb {P}}) \big )(dt) \nonumber \\&\quad = \sup _{(y,b)\in {\mathbb {X}}\times A} \frac{1}{\big ({\mathfrak {w}}(y)\big )(b)} {\mathbb {E}}\big [ |f(X^{y,b},0)| \big ] < \infty . \end{aligned}$$

Lemma 2.2 implies that there exists a unique function $v \in {\mathfrak {W}}$ such that for all $(x,a) \in {\mathbb {X}}\times A$ it holds that

25 $$\begin{aligned} {\mathbb {E}}\Big [ \big |f\big (X^{x,a}, v(X^{x,a})\big )\big | \Big ]&= \int _{\mathbb {X}}| f( y, v(y) ) | \big ( X^{x,a}({\mathbb {P}}) \big )(dy) \nonumber \\&= \int _{\mathbb {X}}| f(y,v(y)) | \kappa _a(x,dy) < \infty , \end{aligned}$$

and

26 $$\begin{aligned} \big (v(x)\big )(a)&= \int _{\mathbb {X}}f(y,v(y)) \kappa _a(x,dy) = \int _{\mathbb {X}}f(y, v(y)) \big ( X^{x,a}({\mathbb {P}}) \big )(dy) \nonumber \\&= {\mathbb {E}}\big [ f\big (X^{x,a}, v(X^{x,a}) \big ) \big ]. \end{aligned}$$

The proof of Corollary 2.3 is thus complete. $\square$

### Lemma 2.4

Let $c_f, c_{\mathfrak {w}}, L \in [0,\infty )$ with $c_{\mathfrak {w}}L < 1$, let $({\mathbb {X}}, {\mathcal {X}})$ be a nonempty measurable space, let $(\Omega , {\mathcal {F}}, {\mathbb {P}})$ be a probability space, let *A* be a nonempty at most countable set, let $\widetilde{{\mathcal {A}}}= \bigotimes _{a \in A} {\mathcal {B}}({\mathbb {R}})$, let $\widetilde{{\mathcal {X}}} = \bigotimes _{a \in A}{\mathcal {X}}$, let $X = \big ( X^{x,a}(\omega )\big )_{x \in {\mathbb {X}},\; a\in A,\; \omega \in \Omega } :{\mathbb {X}}\times \Omega \rightarrow {\mathbb {X}}^A$ be $({\mathcal {X}} \otimes {\mathcal {F}})/\widetilde{{\mathcal {X}}}$-measurable, let $f :{\mathbb {X}}\times {\mathbb {R}}^A \rightarrow {\mathbb {R}}$ be $({\mathcal {X}}\otimes \widetilde{{\mathcal {A}}})/{\mathcal {B}}({\mathbb {R}})$-measurable, let ${\mathfrak {w}}:{\mathbb {X}}\rightarrow (0,\infty )^A$ be ${\mathcal {X}}/\bigotimes _{a \in A}{\mathcal {B}}((0,\infty ))$-measurable, let $v :{\mathbb {X}}\rightarrow {\mathbb {R}}^A$ be ${\mathcal {X}}/\widetilde{{\mathcal {A}}}$-measurable, assume that for all $(x,a)\in {\mathbb {X}}\times A$, $r,s \in {\mathbb {R}}^A$ it holds that $|f(x,r) - f(x,s)| \le L \sup _{b \in A} |r(b) - s(b)|$, $\sup _{(y,b) \in {\mathbb {X}}\times A} \big [ | \big ({\mathfrak {w}}(y)\big )(b)|^{-1} |\big (v(y)\big )(b) | \big ] < \infty$, $\big ( {\mathbb {E}}\big [\sup _{b \in A} \big |\big ({\mathfrak {w}}(X^{x,a})\big )(b)\big |^2\big ] \big )^\frac{1}{2} \le c_{\mathfrak {w}}\big ({\mathfrak {w}}(x)\big )(a)$, $\big ( {\mathbb {E}}\big [ | f(X^{x,a},0) |^2 \big ] \big )^\frac{1}{2}\le c_f \big ({\mathfrak {w}}(x)\big )(a)$, and $\big (v(x)\big )(a) = {\mathbb {E}}[f(X^{x,a},v(X^{x,a}))]$. Then

27 $$\begin{aligned}&\sup _{(x,a) \in {\mathbb {X}}\times A} \frac{| \big (v(x)\big )(a)|}{\big ({\mathfrak {w}}(x)\big )(a)} \le \frac{c_f}{1-c_{\mathfrak {w}}L} \quad \text {and} \hspace{0.2cm}\nonumber \\&\quad \sup _{(x,a) \in {\mathbb {X}}\times A} \frac{\big ( {\mathbb {E}}\big [ \sup _{b \in A} | \big (v(X^{x,a})\big )(b)|^2 \big ] \big )^\frac{1}{2}}{\big ({\mathfrak {w}}(x)\big )(a)} \le \frac{c_f c_{\mathfrak {w}}}{1- c_{\mathfrak {w}}L}. \end{aligned}$$

### Proof of Lemma 2.4

Jensen’s inequality, the triangle inequality, and the assumption that for all $x \in {\mathbb {X}}$, $r,s \in {\mathbb {R}}^A$ it holds that $|f(x,r) - f(x,s)| \le L \sup _{a \in A} |r(a) - s(a)|$ prove that for all $(x,a) \in {\mathbb {X}}\times A$ it holds that

28 $$\begin{aligned} \frac{|\big (v(x)\big )(a)|}{\big ({\mathfrak {w}}(x)\big )(a)}&= \frac{\Big | {\mathbb {E}}\big [ f(X^{x,a}, v(X^{x,a})) \big ] \Big |}{\big ({\mathfrak {w}}(x)\big )(a)} \le \frac{{\mathbb {E}}\big [ |f(X^{x,a}, v(X^{x,a}))|^2 \big ]^{ \frac{1}{2} }}{\big ({\mathfrak {w}}(x)\big )(a)} \nonumber \\&\le L\frac{{\mathbb {E}}\big [ \sup _{b \in A}| \big (v(X^{x,a})\big )(b) |^2 \big ]^\frac{1}{2}}{\big ({\mathfrak {w}}(x)\big )(a)} + \frac{{\mathbb {E}}\big [ |f(X^{x,a}, 0)|^2 \big ]^\frac{1}{2}}{\big ({\mathfrak {w}}(x)\big )(a)} \nonumber \\&\le \frac{L}{\big ({\mathfrak {w}}(x)\big )(a)} {\mathbb {E}}\bigg [ \sup _{b \in A} \bigg \{ \frac{| \big (v(X^{x,a})\big )(b)|}{\big ({\mathfrak {w}}(X^{x,a})\big )(b)} \big ({\mathfrak {w}}(X^{x,a})\big )(b) \bigg \}^2 \bigg ]^\frac{1}{2} + c_f \nonumber \\&\le \frac{L}{\big ({\mathfrak {w}}(x)\big )(a)} \sup _{(y,b) \in {\mathbb {X}}\times A} \bigg \{ \frac{|\big (v(y)\big )(b)|}{\big ({\mathfrak {w}}(y)\big )(b)} \bigg \} {\mathbb {E}}\big [ \sup _{b \in A} {\mathfrak {w}}(X^{x,a})(b)^2 \big ]^\frac{1}{2} + c_f \nonumber \\&\le c_{\mathfrak {w}}L \sup _{(y,b) \in {\mathbb {X}}\times A} \bigg \{ \frac{|\big (v(y)\big )(b)|}{\big ({\mathfrak {w}}(y)\big )(b)} \bigg \} + c_f. \end{aligned}$$

Combining this, the assumption that $\sup _{(x,a)\in {\mathbb {X}}\times A} [|\big ({\mathfrak {w}}(x)\big )(a)|^{-1} |\big (v(x)\big )(a)|] < \infty$, and the assumption that $c_{\mathfrak {w}}L < 1$ shows that

29 $$\begin{aligned} \sup _{(x,a)\in {\mathbb {X}}\times A} \frac{|\big (v(x)\big )(a)|}{\big ({\mathfrak {w}}(x)\big )(a)} \le \frac{c_f}{1- c_{\mathfrak {w}}L}. \end{aligned}$$

This and the assumption that for all $(x,a) \in {\mathbb {X}}\times A$ it holds that ${\mathbb {E}}\big [ |\sup _{b \in A} \big ({\mathfrak {w}}(X^{x,a})\big )(b)|^2 \big ]^\frac{1}{2} \le c_{\mathfrak {w}}\big ({\mathfrak {w}}(x)\big )(a)$ imply that for all $(x,a) \in {\mathbb {X}}\times A$ it holds that

30 $$\begin{aligned} \begin{aligned}&\frac{\big ( {\mathbb {E}}\big [ \sup _{b \in A} |\big (v(X^{x,a})\big )(b)|^2 \big ] \big )^\frac{1}{2}}{\big ({\mathfrak {w}}(x)\big )(a)} \\&\le \bigg [\sup _{(y,b) \in {\mathbb {X}}\times A} \frac{|\big (v(y)\big )(b)|}{\big ({\mathfrak {w}}(y)\big )(b)} \bigg ] \frac{{\mathbb {E}}\big [ \sup _{b \in A} \big ({\mathfrak {w}}(X^{x,a})\big )(b)^2 \big ]^\frac{1}{2}}{\big ({\mathfrak {w}}(x)\big )(a)} \\  &\le \frac{c_f c_{\mathfrak {w}}}{1- c_{\mathfrak {w}}L}. \end{aligned} \end{aligned}$$

Taking the supremum over ${\mathbb {X}}\times A$ yields

31 $$\begin{aligned} \sup _{(x,a) \in {\mathbb {X}}\times A} \frac{\big ( {\mathbb {E}}\big [ \sup _{b \in A} |\big (v(X^{x,a})\big )(b)|^2 \big ] \big )^\frac{1}{2}}{\big ({\mathfrak {w}}(x)\big )(a)} \le \frac{c_f c_{\mathfrak {w}}}{1- c_{\mathfrak {w}}L}. \end{aligned}$$

The proof of Lemma 2.4 is thus complete. $\square$

## Full-History Recursive Multilevel Fixed-Point (MLFP) Approximations

In this section we introduce in Setting 3.1 in Sect. 3.1 below MLFP approximations for functional fixed-point equations, we establish in Lemma 3.2 in Sect. 3.2 below basic measurability properties for MLFP approximations, we establish in Lemma 3.3 in Sect. 3.2 below basic distributional properties for MLFP approximations, and we establish in Lemmas 3.4 and 3.5 in Sect. 3.3 below basic integrability properties for MLFP approximations. In Lemma 3.7 in Sect. 3.4 below we provide a recursive variance estimate for MLFP approximations for functional fixed-point equations. Our proof of Lemma 3.7 in Sect. 3.4 below is based on the measurability, distibutional, and integrability properties for MLFP approximations established in Sects. 3.2 and 3.3. In Proposition 3.8 in Sect. 3.5 below we provide a full error analysis for MLFP approximations for functional fixed-point equations. Our proof of Proposition 3.8 in Sect. 3.5 below is based on the idea to combine the recursive variance estimate in Lemma 3.7 in Sect. 3.4 below with bias-variance decompositions for the approximation errors of the proposed MLFP approximation schemes, and the discrete Gronwall-type two step recursion in [36, Lemma 2.1].

### Mathematical Description of MLFP Approximations

#### Setting 3.1

Let $M \in {\mathbb {N}}$, let $\Theta = \bigcup _{n \in {\mathbb {N}}} {\mathbb {Z}}^n$, let $({\mathbb {X}}, {\mathcal {X}})$ be a nonempty measurable space, let $(\Omega , {\mathcal {F}}, {\mathbb {P}})$ be a probability space, let *A* be a nonempty set, let $\widetilde{{\mathcal {A}}}= \bigotimes _{a \in A} {\mathcal {B}}({\mathbb {R}})$, let $\left\| \cdot \right\| _\infty :{\mathbb {R}}^A \rightarrow [0,\infty ]$ satisfy for all $r \in {\mathbb {R}}^A$ that $\left\| r \right\| _\infty = \sup _{a \in A} \left| r(a) \right|$, let $\widetilde{{\mathcal {X}}} = \bigotimes _{a \in A} {\mathcal {X}}$, let $f:{\mathbb {X}}\times {\mathbb {R}}^A \rightarrow {\mathbb {R}}$ be $({\mathcal {X}} \otimes \widetilde{{\mathcal {A}}})/{\mathcal {B}}({\mathbb {R}})$-measurable, let ${\mathbb {F}}^\theta \subseteq {\mathcal {F}}$, $\theta \in \Theta$, be independent sub-sigma-algebras of ${\mathcal {F}}$, let $X^\theta = \big ( X^{\theta , x, a}(\omega ) \big )_{x \in {\mathbb {X}},\; a \in A,\; \omega \in \Omega } :{\mathbb {X}}\times \Omega \rightarrow {\mathbb {X}}^A$, $\theta \in \Theta$, be i.i.d. random fields, assume for all $\theta \in \Theta$ that $X^\theta$ is $({\mathcal {X}} \otimes {\mathbb {F}}^\theta )/\widetilde{{\mathcal {X}}}$-measurable, let $V_n^\theta :{\mathbb {X}}\times \Omega \rightarrow {\mathbb {R}}^A$, $n \in {\mathbb {N}}_0$, $\theta \in \Theta$, satisfy for all $n \in {\mathbb {N}}_0$, $\theta \in \Theta$, $x \in {\mathbb {X}}$, $a \in A$ that

32 $$\begin{aligned} \big (V_n^\theta (x)\big )(a) =&\sum _{ l = 0}^{n-1} \frac{1}{M^{n - l}} \sum _{i = 1}^{M^{n-l}}\Big [ f\big ( X^{(\theta ,l,i),x,a}, V^{(\theta ,l,i)}_l(X^{(\theta , l ,i),x,a}) \big )\nonumber \\&- \mathbb {1}_{{\mathbb {N}}}(l) f\big ( X^{(\theta ,l,i),x,a}, V^{(\theta ,-l,i)}_{\max \{l - 1, 0\}}(X^{(\theta , l ,i),x,a}) \big ) \Big ]. \end{aligned}$$

### Measurability and Distributional Properties for MLFP Approximations

#### Lemma 3.2

Assume Setting 3.1. Then

(i) it holds for all $n \in {\mathbb {N}}_0$, $\theta \in \Theta$ that $V_n^\theta$ is $({\mathcal {X}} \otimes \sigma ( \bigcup _{\eta \in \Theta } {\mathbb {F}}^{(\theta , \eta )} ))/\widetilde{{\mathcal {A}}}$-measurable and
(ii) it holds for all $n \in {\mathbb {N}}_0$, $\theta , \vartheta \in \Theta$ that
  33 $$\begin{aligned} \begin{aligned} {\mathbb {X}}\times \Omega \ni (x,\omega ) \mapsto \big [ A \ni a \mapsto f\big ( X^{\theta , x, a}(\omega ), V^\vartheta _n(X^{\theta , x, a}(\omega ), \omega ) \big ) \in {\mathbb {R}}\big ] \in {\mathbb {R}}^A \end{aligned} \end{aligned}$$
  is $$({\mathcal {X}} \otimes \sigma ((\bigcup _{\eta \in \Theta } {\mathbb {F}}^{(\vartheta , \eta )}) \cup {\mathbb {F}}^\theta ))/\widetilde{{\mathcal {A}}}$$-measurable.

#### Proof of Lemma 3.2

Note that (32) implies that for all $\theta \in \Theta$, $x \in {\mathbb {X}}$, $a \in A$ it holds that $\big (V_0^\theta (x)\big )(a) = 0$. Hence for all $\theta \in \Theta$ it holds that $V_0^\theta$ is $({\mathcal {X}} \otimes \sigma ( \bigcup _{\eta \in \Theta } {\mathbb {F}}^{(\theta , \eta )} ))/\widetilde{{\mathcal {A}}}$-measurable. Fix $n \in {\mathbb {N}}$ and assume for all $l \in \{0,1,\dots , n-1\}$, $\theta \in \Theta$ that $V_l^\theta$ is $({\mathcal {X}} \otimes \sigma ( \bigcup _{\eta \in \Theta } {\mathbb {F}}^{(\theta , \eta )} ))/\widetilde{{\mathcal {A}}}$-measurable. Observe that for all $l \in \{ 0,1,\dots , n-1\}$, $\theta \in \Theta$ it holds that

34 $$\begin{aligned} {\mathbb {X}}\times \Omega \ni (x,\omega ) \mapsto (x, V_l^\theta (x,\omega )) \in {\mathbb {X}}\times {\mathbb {R}}^A \end{aligned}$$

is $({\mathcal {X}} \otimes \sigma ( \bigcup _{\eta \in \Theta } {\mathbb {F}}^{(\theta , \eta )} )) / ({\mathcal {X}} \otimes \widetilde{{\mathcal {A}}})$-measurable. Since for all $\theta \in \Theta$ it holds that $X^\theta$ is $({\mathcal {X}} \otimes {\mathbb {F}}^\theta )/\widetilde{{\mathcal {X}}}$-measurable it follows that for all $\theta \in \Theta$, $a \in A$ it holds that ${\mathbb {X}}\times \Omega \ni (x, \omega ) \mapsto X^{\theta , x, a}(\omega ) \in {\mathbb {X}}$ is $({\mathcal {X}} \otimes {\mathbb {F}}^\theta )/{\mathcal {X}}$-measurable. This implies that for all $\theta ,\vartheta \in \Theta$, $a \in A$ it holds that ${\mathbb {X}}\times \Omega \ni (x,\omega ) \mapsto (X^{\theta ,x,a}(\omega ),\omega ) \in {\mathbb {X}}\times \Omega$ is $({\mathcal {X}} \otimes \sigma ( (\bigcup _{\eta \in \Theta } {\mathbb {F}}^{(\vartheta , \eta )}) \cup {\mathbb {F}}^\theta ))/({\mathcal {X}} \otimes \sigma ( \bigcup _{\eta \in \Theta } {\mathbb {F}}^{(\vartheta , \eta )} ))$-measurable. This and (34) ensure that for all $l \in \{ 0,1,\dots , n-1\}$, $\theta , \vartheta \in \Theta$, $a \in A$ it holds that ${\mathbb {X}}\times \Omega \ni (x,\omega ) \mapsto \big ( X^{\theta , x,a}(\omega ), V_l^\vartheta (X^{\theta ,x,a}(\omega ), \omega ) \big ) \in {\mathbb {X}}\times {\mathbb {R}}^A$ is $({\mathcal {X}} \otimes \sigma ( (\bigcup _{\eta \in \Theta } {\mathbb {F}}^{(\vartheta , \eta )}) \cup {\mathbb {F}}^\theta ))/({\mathcal {X}} \otimes \widetilde{{\mathcal {A}}})$-measurable. Therefore for all $l \in \{ 0,1,\dots ,n-1\}$, $\theta , \vartheta \in \Theta$, $a \in A$ it holds that ${\mathbb {X}}\times \Omega \ni (x,\omega ) \mapsto f \big ( X^{\theta , x,a}(\omega ), V_l^\vartheta (X^{\theta ,x,a}(\omega ), \omega ) \big ) \in {\mathbb {R}}$ is $({\mathcal {X}} \otimes \sigma ( (\bigcup _{\eta \in \Theta } {\mathbb {F}}^{(\vartheta , \eta )}) \cup {\mathbb {F}}^\theta ))/{\mathcal {B}}({\mathbb {R}})$-measurable. This implies that for all $l \in \{ 0,1,\dots ,n-1 \}$, $\theta , \vartheta \in \Theta$ it holds that

35 $$\begin{aligned} {\mathbb {X}}\times \Omega \ni (x, \omega ) \mapsto \big [A \ni a \mapsto f\big ( X^{\theta , x,a}(\omega ), V_l^\vartheta (X^{\theta ,x,a}(\omega ), \omega )\big ) \in {\mathbb {R}}\big ] \in {\mathbb {R}}^A \end{aligned}$$

is $({\mathcal {X}} \otimes \sigma ( (\bigcup _{\eta \in \Theta } {\mathbb {F}}^{(\vartheta , \eta )}) \cup {\mathbb {F}}^\theta ))/\widetilde{{\mathcal {A}}}$-measurable. Note that for all $l \in \{ 0,1,\dots , n-1\}$, $i \in {\mathbb {N}}$, $\theta \in \Theta$ it holds that $\sigma \big ( (\bigcup _{\eta \in \Theta } {\mathbb {F}}^{(\theta ,l,i,\eta )}) \cup {\mathbb {F}}^{(\theta , l, i)} \big ) \subseteq \sigma \big ( \bigcup _{\eta \in \Theta } {\mathbb {F}}^{(\theta , \eta )} \big )$ and $\sigma \big ( (\bigcup _{\eta \in \Theta } {\mathbb {F}}^{(\theta ,-l,i,\eta )}) \cup {\mathbb {F}}^{(\theta , -l, i)} \big ) \subseteq \sigma \big ( \bigcup _{\eta \in \Theta } {\mathbb {F}}^{(\theta , \eta )} \big )$. This and (35) ensure that for all $\theta \in \Theta$ it holds that $V_n^\theta :{\mathbb {X}}\times \Omega \rightarrow {\mathbb {R}}^A$ is $({\mathcal {X}} \otimes \sigma ( \bigcup _{\eta \in \Theta } {\mathbb {F}}^{(\theta , \eta )} ))/\widetilde{{\mathcal {A}}}$-measurable. Induction hence proves that for all $n \in {\mathbb {N}}_0$, $\theta \in \Theta$ it holds that $V_n^\theta$ is $({\mathcal {X}} \otimes \sigma ( \bigcup _{\eta \in \Theta } {\mathbb {F}}^{(\theta , \eta )} ))/\widetilde{{\mathcal {A}}}$-measurable. This establishes item (i). Note that item (i) and (35) imply that for all $n \in {\mathbb {N}}_0$, $\theta , \vartheta \in \Theta$ it holds that ${\mathbb {X}}\times \Omega \ni (x, \omega ) \mapsto \big [ A \ni a \mapsto f\big ( X^{\theta , x, a}(\omega ), V^\vartheta _n(X^{\theta , x, a}(\omega ), \omega ) \big ) \in {\mathbb {R}}\big ] \in {\mathbb {R}}^A$ is $({\mathcal {X}} \otimes \sigma ((\bigcup _{\eta \in \Theta } {\mathbb {F}}^{(\vartheta , \eta )}) \cup {\mathbb {F}}^\theta ))/\widetilde{{\mathcal {A}}}$-measurable. This demonstrates item (ii). The proof of Lemma 3.2 is thus complete. $\square$

#### Lemma 3.3

Assume Setting 3.1. Then for all $n \in {\mathbb {N}}_0$ it holds that $V_n^\theta$, $\theta \in \Theta$, are identically distributed random fields.

#### Proof of Lemma 3.3

For all $\theta \in \Theta$, $(x,a) \in {\mathbb {X}}\times A$ it holds that $\big (V_0^\theta (x)\big )(a) = 0$. Therefore $V_0^\theta , \theta \in \Theta$, are identically distributed random fields. Fix $n \in {\mathbb {N}}$ and assume that for all $l \in \{0,1,\dots , n-1\}$ it holds that $V_l^\theta$, $\theta \in \Theta$, are identically distributed random fields. Lemma 3.2 and [7, Lemma 2.6] ensure for all $l \in \{ 0,1,\dots , n-1\}$, $i \in {\mathbb {N}}$, $\theta \in \Theta$ that ${\mathbb {X}}\times \Omega \ni (x, \omega ) \mapsto (V_l^{(\theta , l,i)}(x,\omega ), V_{\max \{ l-1,0 \}}^{(\theta , -l,i)}(x,\omega )) \in {\mathbb {R}}^A \times {\mathbb {R}}^A$ and ${\mathbb {X}}\times \Omega \ni (x, \omega ) \mapsto (V_l^{(0, l,i)}(x,\omega ), V_{\max \{l-1,0\}}^{(0, -l,i)}(x,\omega ) ) \in {\mathbb {R}}^A \times {\mathbb {R}}^A$ are identically distributed random fields. This ensures that for all $l \in \{0,1,\dots , n-1\}$, $i\in {\mathbb {N}}$, $\theta \in \Theta$ it holds that ${\mathbb {X}}\times \Omega \ni (x, \omega ) \mapsto f(x,V_l^{(\theta , l,i)}(x,\omega )) - \mathbb {1}_{\mathbb {N}}(l)f(x,V_{\max \{l-1,0 \}}^{(\theta , -l,i)}(x,\omega )) \in {\mathbb {R}}$ and ${\mathbb {X}}\times \Omega \ni (x, \omega ) \mapsto f(x,V_l^{(0, l,i)}(x,\omega )) - \mathbb {1}_{\mathbb {N}}(l)f(x,V_{\max \{l-1,0 \}}^{(0, -l,i)}(x,\omega )) \in {\mathbb {R}}$ are identically distributed random fields. Combining this, the assumption that for all $\theta \in \Theta$ it holds that $X^\theta$ is $({\mathcal {X}} \otimes {\mathbb {F}}^\theta )/\widetilde{{\mathcal {X}}}$-measurable, and [7, Lemma 2.5] establishes that for all $l\in \{ 0,1,\dots ,n-1\}$, $i \in {\mathbb {N}}$, $\theta \in \Theta$ it holds that

36 $$\begin{aligned}&{\mathbb {X}}\times A \times \Omega \ni (x,a,\omega ) \mapsto f\big ( X^{(\theta , l ,i),x,a}(\omega ), V_l^{(\theta , l ,i)}\big ( X^{(\theta , l ,i), x,a}(\omega ), \omega \big )\big )\nonumber \\&\quad - \mathbb {1}_{\mathbb {N}}(l)f\big ( X^{(\theta , l ,i),x,a}(\omega ), V_{\max \{l-1,0\}}^{(\theta , -l ,i)}\big ( X^{(\theta , l ,i), x,a}(\omega ), \omega \big ) \big ) \in {\mathbb {R}}\end{aligned}$$

and

37 $$\begin{aligned}&{\mathbb {X}}\times A \times \Omega \ni (x,a,\omega ) \mapsto f\big ( X^{(0, l ,i),x,a}(\omega ), V_l^{(0, l ,i)}\big ( X^{(0, l ,i), x,a}(\omega ), \omega \big )\big ) \nonumber \\&\quad - \mathbb {1}_{\mathbb {N}}(l)f\big ( X^{(0, l ,i),x,a}(\omega ), V_{\max \{l-1,0\}}^{(0, -l ,i)}\big ( X^{(0, l ,i), x,a}(\omega ), \omega \big ) \big ) \in {\mathbb {R}}\end{aligned}$$

are identically distributed random fields. This implies that for all $l \in \{0,1,\dots ,n-1\}$, $i \in {\mathbb {N}}$, $\theta \in \Theta$ it holds that

38 $$\begin{aligned}&{\mathbb {X}}\times \Omega \ni (x,\omega ) \mapsto \Big [ A \ni a \mapsto f\big ( X^{(\theta , l ,i),x,a}(\omega ), V_l^{(\theta , l ,i)}\big ( X^{(\theta , l ,i), x,a}(\omega ), \omega \big )\big ) \nonumber \\&\quad - \mathbb {1}_{\mathbb {N}}(l)f\big ( X^{(\theta , l ,i),x,a}(\omega ), V_{\max \{l-1,0\}}^{(\theta , -l ,i)}\big ( X^{(\theta , l ,i), x,a}(\omega ), \omega \big ) \big ) \in {\mathbb {R}}\Big ] \in {\mathbb {R}}^A \end{aligned}$$

and

39 $$\begin{aligned}&{\mathbb {X}}\times \Omega \ni (x,\omega ) \mapsto \Big [ A \ni a \mapsto f\big ( X^{(0, l ,i),x,a}(\omega ), V_l^{(0, l ,i)}\big ( X^{(0, l ,i), x,a}(\omega ), \omega \big )\big ) \nonumber \\&\quad - \mathbb {1}_{\mathbb {N}}(l)f\big ( X^{(0, l ,i),x,a}(\omega ), V_{\max \{l-1,0\}}^{(0, -l ,i)}\big ( X^{(0, l ,i), x,a}(\omega ), \omega \big ) \big ) \in {\mathbb {R}}\Big ] \in {\mathbb {R}}^A \end{aligned}$$

are identically distributed random fields. Let $g_k:({\mathbb {R}}^A)^k \rightarrow {\mathbb {R}}^A$, $k \in {\mathbb {N}}$, satisfy for all $k\in {\mathbb {N}}$, $r_1, \dots , r_k \in {\mathbb {R}}^A$ that $g_k(r_1, \dots , r_k) = \sum _{j = 1}^kr_j$. Note that for all $k \in {\mathbb {N}}$ it holds that $g_k$ is $\widetilde{{\mathcal {A}}}^{\otimes k}/\widetilde{{\mathcal {A}}}$-measurable. Let $U^\theta _{l,i} :{\mathbb {X}}\times \Omega \rightarrow {\mathbb {R}}^A$, $l \in \{ 0,1,\dots , n-1\}$, $i \in {\mathbb {N}}$, $\theta \in \Theta$, satisfy for all $l \in \{0,1,\dots ,n-1\}$, $i \in {\mathbb {N}}$, $\theta \in \Theta$, $(x,\omega ) \in {\mathbb {X}}\times \Omega$ that

40 $$\begin{aligned} U^\theta _{l,i}(x,\omega )&= \big [ A \ni a \mapsto f\big ( X^{(\theta , l ,i),x,a}(\omega ), V_l^{(\theta , l ,i)}\big ( X^{(\theta , l ,i), x,a}(\omega ), \omega \big )\big ) \nonumber \\&\quad - \mathbb {1}_{\mathbb {N}}(l)f\big ( X^{(\theta , l ,i),x,a}(\omega ), V_{\max \{l-1,0\}}^{(\theta , -l ,i)}\big ( X^{(\theta , l ,i), x,a}(\omega ), \omega \big ) \big ) \in {\mathbb {R}}\big ]. \end{aligned}$$

Lemma 3.2 demonstrates that for all $l \in \{0,1,\dots ,n-1\}$, $i \in {\mathbb {N}}$, $\theta \in \Theta$ it holds that $U^\theta _{l,i}$ is $({\mathcal {X}} \otimes \sigma ( (\bigcup _{\eta \in \Theta } {\mathbb {F}}^{(\theta , l,i,\eta )} \cup {\mathbb {F}}^{(\theta , -l,i,\eta )}) \cup {\mathbb {F}}^{(\theta , l,i)} ) )/\widetilde{{\mathcal {A}}}$-measurable. Note that (32) ensures for all $(x,\omega ) \in {\mathbb {X}}\times \Omega$, $\theta \in \Theta$ that $V_n^\theta (x, \omega ) = \sum _{l = 0}^{n-1} \frac{1}{M^{n-l}} \sum _{i = 1}^{M^{n-l}} U^\theta _{l,i}(x,\omega )$. Let $\widetilde{M} = \sum _{j = 1}^nM^j \in {\mathbb {N}}$, let $Y^\theta _n :{\mathbb {X}}\times \Omega \rightarrow ({\mathbb {R}}^A)^{\widetilde{M}}$, $\theta \in \Theta$, satisfy for all $\theta \in \Theta$ that

41 $$\begin{aligned} Y^\theta _n&= \Big ( \frac{1}{M^n} U^\theta _{0,1}, \frac{1}{M^n} U^\theta _{0,2}, \dots , \frac{1}{M^n} U^\theta _{0,M^n} ,\nonumber \\&\quad \frac{1}{M^{n-1}}U^\theta _{1,1}, \frac{1}{M^{n-1}} U^\theta _{1,2},\dots , \frac{1}{M^{n-1}} U^\theta _{1,M^{n-1}} , \dots \nonumber \\&\quad \dots , \frac{1}{M} U^\theta _{n-1,1}, \frac{1}{M} U^\theta _{n-1,2}, \dots , \frac{1}{M} U^\theta _{n-1,M} \Big ). \end{aligned}$$

Observe that for all $(x,\omega ) \in {\mathbb {X}}\times \Omega$, $\theta \in \Theta$ it holds that

42 $$\begin{aligned} \begin{aligned} g_{\widetilde{M}} (Y^\theta _n(x,\omega )) = \sum _{l = 0}^{n-1} \frac{1}{M^{n-l}} \sum _{i = 1}^{M^{n-l}} U^\theta _{l,i}(x,\omega ) = V_n^\theta (x, \omega ). \end{aligned} \end{aligned}$$

The fact that for all $l \in \{ 0,1,\dots , n-1 \}$, $i \in {\mathbb {N}}$, $\theta \in \Theta$ it holds that $U_{l,i}^\theta$ is $({\mathcal {X}} \otimes \sigma ( (\bigcup _{\eta \in \Theta } {\mathbb {F}}^{(\theta , l,i,\eta )} \cup {\mathbb {F}}^{(\theta , -l,i,\eta )}) \cup {\mathbb {F}}^{(\theta , l,i)} ) )/\widetilde{{\mathcal {A}}}$-measurable, the assumption that $({\mathbb {F}}^\theta )_{\theta \in \Theta }$ are independent sigma-algebras, and [7, Lemma 2.6] prove for all $\theta \in \Theta$ that $Y^\theta _n$ and $Y^0_n$ are identically distributed random fields. This implies for all $\theta \in \Theta$ that $V_n^\theta$ and $V_n^0$ are identically distributed random fields. Induction and the fact that $V_0^\theta$, $\theta \in \Theta ,$ are identically distributed random fields prove that for all $n \in {\mathbb {N}}_0$ it holds that $V_n^\theta$, $\theta \in \Theta$, are identically distributed random fields. The proof of Lemma 3.3 is thus complete. $\square$

### Integrability Properties for MLFP Approximations

#### Lemma 3.4

Assume Setting 3.1, assume *A* is finite, let $L \in [0,\infty )$, let ${\mathfrak {w}}:{\mathbb {X}}\rightarrow (0, \infty )$ be ${\mathcal {X}}/ {\mathcal {B}}((0,\infty ))$-measurable, assume that for all $(x,a) \in {\mathbb {X}}\times A$, $r,s \in {\mathbb {R}}^A$ it holds that $|f(x,r) - f(x,s)| \le L \max _{b \in A} |r(b) - s(b)|$, and $\big ( {\mathbb {E}}\big [ |f(X^{0,x,a}, 0 )|^2 + |{\mathfrak {w}}(X^{0,x,a})|^2 \big ] \big )^\frac{1}{2} \le L{\mathfrak {w}}(x)$. Then it holds for all $n \in {\mathbb {N}}_0$ that $\sup _{x \in {\mathbb {X}}} \big ( {\mathbb {E}}\big [ \max _{a \in A} \left| \big (V_n^0(x)\big )(a) \right| ^2 \big ] |{\mathfrak {w}}(x)|^{-2} \big ) < \infty$.

#### Proof of Lemma 3.4

For all $(x,a) \in {\mathbb {X}}\times A$, $\theta \in \Theta$ it holds that $\big (V_0^\theta (x)\big )(a) = 0$. Hence it holds that $\sup _{x \in {\mathbb {X}}} ({\mathbb {E}}\big [ \Vert V_0^0(x) \Vert ^2_\infty \big ] |{\mathfrak {w}}(x)|^{-2} ) < \infty$. Fix $n \in {\mathbb {N}}$ and assume for all $l \in \{ 0,1,\dots ,n-1\}$ that $\sup _{x \in {\mathbb {X}}} ({\mathbb {E}}\big [ \Vert V_l^0(x) \Vert ^2_\infty \big ] |{\mathfrak {w}}(x)|^{-2} ) < \infty$. Note that for all $m \in {\mathbb {N}}$ it holds that

43 $$\begin{aligned} \sup _{x \in {\mathbb {X}}} \frac{{\mathbb {E}}\big [ \Vert V_m^0(x) \Vert ^2_\infty \big ]}{|{\mathfrak {w}}(x)|^2}&\le \sup _{x \in {\mathbb {X}}} \sum _{a \in A} \frac{{\mathbb {E}}\big [ |\big (V_m^0(x)\big )(a)|^2 \big ]}{|{\mathfrak {w}}(x)|^2} \nonumber \\&\le \sum _{a \in A} \sup _{x \in X} \frac{{\mathbb {E}}\big [ |\big (V_m^0(x)\big )(a)|^2 \big ]}{|{\mathfrak {w}}(x)|^2} . \end{aligned}$$

Lemma 3.3 and Jensen’s inequality prove that for all $m \in {\mathbb {N}}$, $(x, a) \in {\mathbb {X}}\times A$, $\theta \in \Theta$ it holds that

44 $$\begin{aligned} {\mathbb {E}}\big [ |\big (V_m^0(x)\big )(a)|^2 \big ]&= {\mathbb {E}}\big [ |\big (V_m^\theta (x)\big )(a)|^2 \big ] \nonumber \\&\le \sum _{l = 0}^{m-1} \frac{m}{M^{m-l}} \sum _{i = 1}^{M^{m-l}} {\mathbb {E}}\Big [ \big | f\big ( X^{(\theta , l,i), x,a}, V_l^{(\theta ,l,i)}(X^{(\theta , l, i),x,a})\big ) \nonumber \\&\quad - \mathbb {1}_{\mathbb {N}}(l)f\big ( X^{(\theta , l, i), x, a}, V_{\max \{ l-1,0\}}^{(\theta , -l, i)}(X^{(\theta , l, i), x, a}) \big ) \big |^2 \Big ] \nonumber \\&\le \sum _{l = 0}^{m-1} \frac{2m}{M^{m-l}} \sum _{i = 1}^{M^{m-l}} {\mathbb {E}}\big [ |f\big ( X^{(\theta , l,i), x,a}, V_l^{(\theta ,l,i)}(X^{(\theta , l, i),x,a})\big )|^2 \big ] \nonumber \\&\quad + {\mathbb {E}}\big [ |f\big ( X^{(\theta , l, i), x, a}, V_{\max \{ l-1,0\}}^{(\theta , -l, i)}(X^{(\theta , l, i), x, a}) \big )|^2 \big ]. \end{aligned}$$

The triangle inequality, Jensen’s inequality, the assumption that for all $x \in {\mathbb {X}}$, $r, s \in {\mathbb {R}}^A$ it holds that $|f(x,r) - f(x,s)| \le L \max _{a \in A} |r(a) - s(a)|$, and the assumption that for all $(x,a) \in {\mathbb {X}}\times A$ it holds that $\big ( {\mathbb {E}}\big [ |f(X^{0,x,a}, 0)|^2 \big ] \big )^\frac{1}{2} \le L{\mathfrak {w}}(x)$ ensure that for all $m \in {\mathbb {N}}_0$, $(x,a) \in {\mathbb {X}}\times A$, $\theta , \vartheta \in \Theta$ it holds that

45 $$\begin{aligned}&{\mathbb {E}}\big [ |f\big ( X^{\theta , x, a}, V_m^\vartheta (X^{\theta , x,a }) \big )|^2 \big ] \nonumber \\&\quad \le 2 {\mathbb {E}}\big [ |f\big ( X^{\theta , x, a}, V_m^\vartheta (X^{\theta , x,a }) \big ) - f(X^{\theta , x, a}, 0)|^2 \big ] + 2 {\mathbb {E}}\big [ |f(X^{\theta , x, a}, 0)|^2 \big ] \nonumber \\&\quad \le 2L^2 {\mathbb {E}}\big [ \max _{b \in A} |\big (V_m^\vartheta (X^{\theta , x, a})\big )(b)|^2 \big ] + 2L^2|{\mathfrak {w}}(x)|^2. \end{aligned}$$

Lemma 3.3 ensures for all $m \in {\mathbb {N}}$, $\vartheta \in \Theta$ that $V_m^\vartheta$ and $V_m^0$ are identically distributed. Combining this and the assumption that *A* is finite demonstrates that for all $m \in {\mathbb {N}}$, $\vartheta \in \Theta$ it holds that ${\mathbb {X}}\times \Omega \ni (x, \omega ) \mapsto \Vert V_m^\vartheta (x,w) \Vert ^2_\infty \in [0,\infty )$ and ${\mathbb {X}}\times \Omega \ni (x,\omega ) \mapsto \Vert V_m^0(x,\omega ) \Vert ^2_\infty \in [0,\infty )$ are identically distributed. This, Lemma 3.2, the assumption that for all $\theta \in \Theta$ it holds that $X^\theta$ is $({\mathcal {X}} \otimes {\mathbb {F}}^\theta )/\widetilde{{\mathcal {X}}}$-measurable, the assumption that the sigma-algebras ${\mathbb {F}}^\theta$, $\theta \in \Theta$, are independent, and [33, Lemma 2.2] imply that for all $m \in {\mathbb {N}}_0$, $(x,a) \in {\mathbb {X}}\times A$, $\theta , \vartheta \in \Theta$ with $\theta \not \in \bigcup _{\eta \in \Theta } \{(\vartheta , \eta )\}$ it holds that

46 $$\begin{aligned} {\mathbb {E}}\big [ \Vert V_m^\vartheta (X^{\theta , x,a}) \Vert ^2_\infty \big ]&= \int _{\mathbb {X}}{\mathbb {E}}\big [ \Vert V_m^\vartheta (y) \Vert ^2_\infty \big ] \big ( X^{\theta , x, a} ({\mathbb {P}}) \big )(dy) \nonumber \\&= \int _{\mathbb {X}}{\mathbb {E}}\big [ \Vert V_m^0(y) \Vert ^2_\infty \big ] \big ( X^{0,x,a} ({\mathbb {P}}) \big )(dy). \end{aligned}$$

This, (45), the assumption that for all $(x, a) \in {\mathbb {X}}\times A$ it holds that $({\mathbb {E}}\big [ |{\mathfrak {w}}(X^{0,x,a})|^2 \big ])^\frac{1}{2} \le L{\mathfrak {w}}(x)$, and the assumption that for all $l \in \{0,1,\dots , n-1\}$ it holds that $\sup _{x \in {\mathbb {X}}}( {\mathbb {E}}\big [ \Vert V_l^0(x) \Vert ^2_\infty \big ] |{\mathfrak {w}}(x)|^{-2}) < \infty$ prove for all $l \in \{ 0,1, \dots , n-1\}$, $(x,a) \in {\mathbb {X}}\times A$, $\theta , \vartheta \in \Theta$ with $\theta \not \in \bigcup _{\eta \in \Theta } \{(\vartheta , \eta )\}$ that

47 $$\begin{aligned}&\frac{ {\mathbb {E}}\big [ |f\big ( X^{\theta , x, a}, V_l^\vartheta (X^{\theta , x,a }) \big )|^2 \big ]}{|{\mathfrak {w}}(x)|^2} \nonumber \\&\quad \le \frac{2L^2}{|{\mathfrak {w}}(x)|^2} \int _{\mathbb {X}}{\mathbb {E}}\big [ \Vert V_l^0(y) \Vert ^2_\infty \big ] \big ( X^{0,x,a} ({\mathbb {P}}) \big )(dy) + 2L^2 \nonumber \\&\quad \le 2L^2 \bigg (\sup _{y \in {\mathbb {X}}} \frac{{\mathbb {E}}\big [ \Vert V_l^0(y) \Vert ^2_\infty \big ]}{|{\mathfrak {w}}(y)|^2}\bigg ) \bigg ( \frac{{\mathbb {E}}\big [ |{\mathfrak {w}}(X^{0,x,a})|^2 \big ]}{|{\mathfrak {w}}(x)|^2} \bigg ) + 2L^2 \nonumber \\&\quad \le 2L^4 \bigg (\sup _{y \in {\mathbb {X}}} \frac{{\mathbb {E}}\big [ \Vert V_l^0(y) \Vert ^2_\infty \big ]}{|{\mathfrak {w}}(y)|^2}\bigg ) + 2L^2. \end{aligned}$$

This ensures that for all $l \in \{ 0,1,\dots ,n-1 \}$, $\theta , \vartheta \in \Theta$ with $\theta \not \in \bigcup _{\eta \in \Theta } \{ (\vartheta , \eta ) \}$ that

48 $$\begin{aligned} \sup _{(x,a) \in {\mathbb {X}}\times A} \frac{ {\mathbb {E}}\big [ |f\big ( X^{\theta , x, a}, V_l^\vartheta (X^{\theta , x,a }) \big )|^2 \big ]}{|{\mathfrak {w}}(x)|^2}&\le 2L^4 \bigg (\sup _{y \in {\mathbb {X}}} \frac{{\mathbb {E}}\big [ \Vert V_l^0(y) \Vert ^2_\infty \big ]}{|{\mathfrak {w}}(y)|^2}\bigg ) + 2L^2 < \infty . \end{aligned}$$

This and (44) demonstrate for all $a \in A$ that $\sup _{x \in {\mathbb {X}}} \big ( {\mathbb {E}}\big [ |\big (V_n^0(x)\big )(a)|^2 \big ] |{\mathfrak {w}}(x)|^{-2} \big ) < \infty$. This, the assumption that *A* is finite, and (43) show that $\sup _{x \in {\mathbb {X}}} \big ({\mathbb {E}}\big [ \Vert V_n^0(x) \Vert ^2_\infty \big ] |{\mathfrak {w}}(x)|^{-2} \big ) < \infty$. Induction hence proves that for all $n \in {\mathbb {N}}_0$ it holds that $\sup _{x \in {\mathbb {X}}} \big ( {\mathbb {E}}\big [ \max _{a \in A} | \big (V_n^0(x)\big )(a) |^2 \big ] |{\mathfrak {w}}(x)|^{-2} \big ) < \infty$. The proof of Lemma 3.4 is thus complete. $\square$

#### Lemma 3.5

Assume Setting 3.1, assume *A* is finite, let $L \in [0,\infty )$, let ${\mathfrak {w}}:{\mathbb {X}}\rightarrow (0, \infty )$ be ${\mathcal {X}}/{\mathcal {B}}((0,\infty ))$-measurable, assume that for all $(x,a) \in {\mathbb {X}}\times A$, $r,s \in {\mathbb {R}}^A$ it holds that $|f(x,r) - f(x,s)| \le L \max _{b \in A} |r(b) - s(b)|,$ $\big ( {\mathbb {E}}\big [ |f(X^{0,x,a}, 0)|^2 + |{\mathfrak {w}}(X^{0,x,a})|^2 \big ] \big )^\frac{1}{2} \le L {\mathfrak {w}}(x)$. Then it holds for all $n \in {\mathbb {N}}$, $(x,a) \in {\mathbb {X}}\times A$, $\theta \in \Theta$ that ${\mathbb {E}}\big [ |f(X^{0,x,a}, V_{n-1}^0(X^{0,x,a}))| + |\big (V_n^\theta (x)\big )(a)| \big ] < \infty$ and ${\mathbb {E}}\big [ \big (V_n^\theta (x)\big )(a) \big ] = {\mathbb {E}}\big [ f(X^{0,x,a}, V_{n-1}^0(X^{0,x,a})) \big ]$.

#### Proof of Lemma 3.5

The assumption that for all $x \in {\mathbb {X}}$, $r,s \in {\mathbb {R}}^A$ it holds that $|f(x,r) - f(x,s)| \le L \max _{a \in A} |r(a) - s(a)|$, Lemmas 3.3, 3.4, the fact that $0 \not \in \bigcup _{\eta \in \Theta } \{(0,\eta )\}$, the assumption that $\big ( {\mathbb {E}}\ \big [ |f(X^{0,x,a}, 0)|^2 \big ] \big )^\frac{1}{2} \le L{\mathfrak {w}}(x)$, and [33, Lemma 2.2] prove that for all $n \in {\mathbb {N}}$, $(x,a) \in {\mathbb {X}}\times A$, $\theta \in \Theta$ it holds that ${\mathbb {E}}\big [ |f( X^{0,x,a}, V_{n-1}^0(X^{0,x,a}) )|^2 + |\big (V_n^\theta (x)\big )(a)|^2 \big ] < \infty$. This ensures for all $n \in {\mathbb {N}}$, $(x,a) \in {\mathbb {X}}\times A$, $\theta \in \Theta$ that ${\mathbb {E}}\big [ |f( X^{0,x,a}, V_{n-1}^0(X^{0,x,a}) )| + |\big (V_n^\theta (x)\big )(a)| \big ] < \infty$. This, Lemma 3.2, the assumption that $X^\theta$, $\theta \in \Theta$, are i.i.d. random fields, Lemma 3.3, and [33, Lemma 2.2] demonstrate that for all $n \in {\mathbb {N}}_0$, $(x,a) \in {\mathbb {X}}\times A$, $\theta , \vartheta \in \Theta$ with $\theta \not \in \bigcup _{\eta \in \Theta } \{(\vartheta , \eta )\}$ it holds that

49 $$\begin{aligned} {\mathbb {E}}\big [ f\big ( X^{\theta , x,a}, V_n^\vartheta (X^{\theta ,x,a}) \big ) \big ]&= {\mathbb {E}}\big [ f\big ( X^{0,x,a}, V_n^0(X^{0,x,a}) \big ) \big ]. \end{aligned}$$

This establishes for all $n \in {\mathbb {N}}$, $(x,a) \in {\mathbb {X}}\times A$, $\theta \in \Theta$ that

50 $$\begin{aligned} {\mathbb {E}}\big [ \big (V_n^\theta (x)\big )(a) \big ]&= \sum _{l = 0}^{n-1} \frac{1}{M^{n-l}} \sum _{i = 1}^{M^{n-l}} {\mathbb {E}}\big [ f\big ( X^{(\theta , l, i),x,a}, V_l^{(\theta , l, i)}(X^{(\theta ,l,i),x,a}) \big ) \big ] \nonumber \\&\quad - \mathbb {1}_{{\mathbb {N}}}(l) {\mathbb {E}}\big [ f\big ( X^{(\theta , l, i),x,a}, V_{\max \{l-1,0 \}}^{(\theta , -l ,i)}(X^{(\theta , l ,i), x, a}) \big ) \big ] \nonumber \\&= \sum _{l = 0}^{n-1} {\mathbb {E}}\big [ f\big ( X^{0,x,a}, V_l^0(X^{0,x,a}) \big ) \big ]\nonumber \\&\quad - \mathbb {1}_{{\mathbb {N}}}(l) {\mathbb {E}}\big [ f \big ( X^{0,x,a}, V_{\max \{ l-1,0 \}}^0(X^{0,x,a}) \big ) \big ] \nonumber \\&= {\mathbb {E}}\big [ f \big ( X^{0,x,a}, V_{n-1}^0(X^{0,x,a}) \big ) \big ]. \end{aligned}$$

The proof of Lemma 3.5 is thus complete. $\square$

### Recursive Error Bounds for MLFP Approximations

#### Lemma 3.6

Assume Setting 3.1, assume *A* is finite, let $c, L \in [0,\infty )$, let ${\mathfrak {w}}:{\mathbb {X}}\rightarrow (0, \infty )$ be ${\mathcal {X}}/{\mathcal {B}}((0,\infty ))$-meaurable, let $v :{\mathbb {X}}\rightarrow {\mathbb {R}}^A$ be ${\mathcal {X}}/\widetilde{{\mathcal {A}}}$-measurable, assume that for all $(x,a) \in {\mathbb {X}}\times A$, $r,s \in {\mathbb {R}}^A$ it holds that $|f(x,r) - f(x,s)| \le L \max _{b \in A} |r(b) - s(b)|$, $\big ( {\mathbb {E}}\big [ |f(X^{0,x,a}, 0)|^2 + \Vert v(X^{0,x,a}) \Vert _\infty ^2 + \left| {\mathfrak {w}}(X^{0,x,a}) \right| ^2 \big ] \big )^\frac{1}{2} \le c {\mathfrak {w}}(x)$, and $\big (v(x)\big )(a) = {\mathbb {E}}[ f(X^{0,x,a}, v(X^{0,x,a})) ]$. Then it holds for all $n \in {\mathbb {N}}$, $(x,a) \in {\mathbb {X}}\times A$, $\theta \in \Theta$ that

51 $$\begin{aligned} \big | \big (v(x)\big )(a) - {\mathbb {E}}\big [ \big (V_n^\theta (x)\big )(a) \big ] \big | \le L \left( {\mathbb {E}}\Big [ \big \Vert v(X^{0,x,a}) - V_{n-1}^0(X^{0,x,a}) \big \Vert ^2_\infty \Big ] \right) ^{\frac{1}{2}}. \end{aligned}$$

#### Proof of Lemma 3.6

The assumption that for all $(x,a) \in {\mathbb {X}}\times A$ it holds that $\big (v(x)\big )(a) = {\mathbb {E}}\big [ f(X^{0,x,a}, v(X^{0,x,a})) \big ]$, Lemma 3.5, the assumption that for all $x \in {\mathbb {X}}$, $r,s \in {\mathbb {R}}^A$ it holds that $\left| f(x,r) - f(x,s) \right| \le L \max _{a \in A} \left| r(a) - s(a) \right|$, Lemma 3.4, and the Cauchy-Schwarz inequality imply that for all $n \in {\mathbb {N}}$, $(x,a) \in {\mathbb {X}}\times A$, $\theta \in \Theta$ it holds that

52 $$\begin{aligned}&\left| v(x)(a) - {\mathbb {E}}\big [ V_n^\theta (x)(a)\big ] \right| \nonumber \\&\quad \le {\mathbb {E}}\big [ \big | f\big ( X^{0,x,a}, v(X^{0,x,a}) \big ) - f\big ( X^{0,x,a}, V_{n-1}^0(X^{0,x,a}) \big ) \big | \big ] \nonumber \\&\quad \le L {\mathbb {E}}\Big [ \big \Vert v(X^{0,x,a})-V_{n-1}^0(X^{0,x,a}) \big \Vert _\infty \Big ] \nonumber \\&\quad \le L \Big ( {\mathbb {E}}\Big [ \big \Vert v(X^{0,x,a}) - V_{n-1}^0(X^{0,x,a}) \big \Vert ^2_\infty \Big ] \Big )^\frac{1}{2}. \end{aligned}$$

The proof of Lemma 3.6 is thus complete. $\square$

#### Lemma 3.7

Assume Setting 3.1, assume *A* is finite, let $c,L \in [0, \infty )$, let ${\mathfrak {w}}:{\mathbb {X}}\rightarrow (0,\infty )$ be ${\mathcal {X}}/{\mathcal {B}}((0,\infty ))$-measurable, let $v :{\mathbb {X}}\rightarrow {\mathbb {R}}^A$ be ${\mathcal {X}}/\widetilde{{\mathcal {A}}}$-measurable, assume that for all $(x,a)\in {\mathbb {X}}\times A$, $r,s \in {\mathbb {R}}^A$ it holds that $|f(x,r) - f(x,s)| \le L \max _{b \in A} |r(b) - s(b)|$, $\big ( {\mathbb {E}}\big [ |f(X^{0,x,a}, 0)|^2 + \Vert v(X^{0,x,a}) \Vert _\infty ^2 + \left| {\mathfrak {w}}(X^{0,x,a})\right| ^2\big ] \big )^\frac{1}{2} \le c{\mathfrak {w}}(x)$, and $\big (v(x)\big )(a) = {\mathbb {E}}\big [ f(X^{0,x,a}, v(X^{0,x,a})) \big ]$. Then it holds for all $n \in {\mathbb {N}}$, $(x,a) \in {\mathbb {X}}\times A$, $\theta \in \Theta$ that

53 $$\begin{aligned} \begin{aligned}&\left( \operatorname {Var}\left[ \big (V_n^\theta (x)\big )(a) \right] \right) ^\frac{1}{2} \\  &\quad \le \frac{1}{\sqrt{M^n}} \Big ( {\mathbb {E}}\big [ |f( X^{0,x,a},0 )|^2 \big ] \Big )^\frac{1}{2} + \mathbb {1}_{[2, \infty )}(n) \frac{L}{\sqrt{M}} \big ( {\mathbb {E}}\big [ \Vert V_{n-1}^0(X^{0,x,a}) - v(X^{0,x,a}) \Vert ^2_\infty \big ] \big )^\frac{1}{2} \\  &\quad + \mathbb {1}_{[2, \infty )}(n) \frac{L\sqrt{M}}{\sqrt{M^n}} \big ( {\mathbb {E}}\big [ \Vert v(X^{0,x,a}) \Vert ^2_\infty \big ] \big )^\frac{1}{2} \\  &+ \sum _{l = 1}^{n - 2} \frac{L(1 + \sqrt{M})}{\sqrt{M^{n-l}}} \big ( {\mathbb {E}}\big [ \Vert V_l^0(X^{0,x,a}) - v(X^{0,x,a}) \Vert ^2_\infty \big )^\frac{1}{2}. \end{aligned} \end{aligned}$$

#### Proof of Lemma 3.7

Lemma 3.4 demonstrates for all $n \in {\mathbb {N}}$, $(x,a) \in {\mathbb {X}}\times A$, $\theta \in \Theta$ that $\operatorname {Var}\big [ \big (V_n^\theta (x)\big )(a) \big ] < \infty$. Lemma 3.2 yields that for all $l,i \in {\mathbb {N}}_0$, $(x,a) \in {\mathbb {X}}\times A$, $\theta \in \Theta$ it holds that

54 $$\begin{aligned} \Omega \ni \omega&\mapsto f \big ( X^{(\theta , l, i), x, a}(\omega ), V_l^{(\theta , l, i)}(X^{(\theta , l, i), x, a}(\omega ), \omega ) \big ) \nonumber \\&\quad -\mathbb {1}_{\mathbb {N}}(l) f\big ( X^{(\theta , l, i),x,a}(\omega ), V_{\max \{l-1,0\}}^{(\theta , -l, i)}(X^{(\theta , l, i),x ,a}(\omega ), \omega ) \big ) \in {\mathbb {R}}\end{aligned}$$

is $\sigma \big ( (\bigcup _{\eta \in \Theta } {\mathbb {F}}^{(\theta , l, i, \eta )} \cup {\mathbb {F}}^{(\theta , -l, i, \eta )}) \cup {\mathbb {F}}^{(\theta , l, i)} \big ) / {\mathcal {B}}({\mathbb {R}})$-measurable. This demonstrates that for all $n \in {\mathbb {N}}_{0}$, $(x,a) \in {\mathbb {X}}\times A$ it holds that

55 $$\begin{aligned} \operatorname {Var}\left[ \big (V_n^\theta (x)\big )(a) \right]&= \sum _{l = 0}^{n-1} \frac{1}{(M^{n-l})^2} \sum _{i = 1}^{M^{n-l}} \operatorname {Var}\Big [ f\big ( X^{(\theta , l,i),x,a}, V_l^{(\theta , l,i)}(X^{(\theta , l, i),x,a}) \big )\nonumber \\&\quad - \mathbb {1}_{\mathbb {N}}(l) f\big ( X^{(\theta , l ,i),x,a}, V_{\max \{ l-1,0 \}}^{(\theta , -l ,i)}(X^{(\theta , l ,i), x ,a}) \big ) \Big ]. \end{aligned}$$

Lemma 3.2, Lemma 3.3, and [7, Lemma 2.6] ensure that for all $l,i \in {\mathbb {N}}_{0}$, $\theta \in \Theta$ it holds that

56 $$\begin{aligned} {\mathbb {X}}\times \Omega \ni (x, \omega )&\mapsto \big ( V_l^{(\theta , l, i)}(x,\omega ), V_{\max \{ l-1,0 \}}^{(\theta , -l ,i)}(x,\omega ) \big ) \in {\mathbb {R}}^A \times {\mathbb {R}}^A \quad \text {and} \nonumber \\ {\mathbb {X}}\times \Omega \ni (x, \omega )&\mapsto \big ( V_l^0(x,\omega ), V^1_{\max \{ l-1, 0 \}}(x, \omega ) \big ) \in {\mathbb {R}}^A \times {\mathbb {R}}^A. \end{aligned}$$

are identically distributed random fields. This and the fact that *f* is $({\mathcal {X}} \otimes \widetilde{{\mathcal {A}}})/{\mathcal {B}}({\mathbb {R}})$-measurable show that for all $l,i \in {\mathbb {N}}_{0}$, $\theta \in \Theta$ it holds that

57 $$\begin{aligned} {\mathbb {X}}\times \Omega \ni (x, \omega )&\mapsto f(x, V_l^{(\theta , l,i)}(x,\omega )) - \mathbb {1}_{\mathbb {N}}(l) f( x, V_{\max \{ l-1, 0 \}}^{(\theta , -l, i)}(x,\omega ) ) \in {\mathbb {R}}\quad \text {and} \nonumber \\ {\mathbb {X}}\times \Omega \ni (x, \omega )&\mapsto f( x, V_l^0(x, \omega ) ) - \mathbb {1}_{\mathbb {N}}(l) f(x , V_{\max \{ l-1,0 \}}^1(x,\omega )) \in {\mathbb {R}}\end{aligned}$$

are identically distributed random fields. This and [7, Lemma 2.5] prove that for all $l,i \in {\mathbb {N}}_0$, $\theta \in \Theta$ it holds that

58 $$\begin{aligned}&{\mathbb {X}}\times A \times \Omega \ni (x,a,\omega ) \nonumber \\&\quad \mapsto f\big ( X^{(\theta , l, i), x, a}(\omega ), V_l^{(\theta , l, i)}( X^{(\theta , l, i),x,a}(\omega ) , \omega ) \big ) \nonumber \\&\quad \quad - \mathbb {1}_{\mathbb {N}}(l) f\big ( X^{(\theta , l, i), x, a}(\omega ), V_{\max \{ l-1, 0 \}}^{(\theta , -l,i)} (X^{(\theta , l, i), x, a}(\omega ), \omega ) \big ) \in {\mathbb {R}}\quad \text {and} \nonumber \\&{\mathbb {X}}\times A \times \Omega \ni (x,a,\omega ) \nonumber \\&\quad \mapsto f\big ( X^{0, x, a}(\omega ), V_l^0( X^{0,x,a}(\omega ) , \omega ) \big ) \nonumber \\&\quad \quad - \mathbb {1}_{\mathbb {N}}(l) f\big ( X^{0, x, a}(\omega ), V_{\max \{ l-1, 0 \}}^1 (X^{0, x, a}(\omega ), \omega ) \big ) \in {\mathbb {R}}\end{aligned}$$

are identically distributed random fields. This implies for all $l,i \in {\mathbb {N}}_{0}$, $(x,a) \in {\mathbb {X}}\times A$, $\theta \in \Theta$ that

59 $$\begin{aligned}&\operatorname {Var}\Big [ f\big ( X^{(\theta , l,i),x,a}, V_l^{(\theta , l,i)}(X^{(\theta , l, i),x,a}) \big ) - \mathbb {1}_{\mathbb {N}}(l) f\big ( X^{(\theta , l ,i),x,a}, V_{\max \{ l-1,0 \}}^{(\theta , -l ,i)}(X^{(\theta , l ,i), x ,a}) \big ) \Big ]\nonumber \\&\quad = \operatorname {Var}\Big [ f\big ( X^{0,x,a}, V_l^0(X^{0,x,a}) \big ) - \mathbb {1}_{\mathbb {N}}(l) f\big ( X^{0,x,a}, V_{\max \{ l-1,0 \}}^1(X^{0, x ,a}) \big ) \Big ]. \end{aligned}$$

This and (55) ensure that for all $n \in {\mathbb {N}}$, $(x, a) \in {\mathbb {X}}\times A$, $\theta \in \Theta$ it holds that

60 $$\begin{aligned}&\operatorname {Var}\big [ \big (V_n^\theta (x)\big )(a)\big ] \nonumber \\&\quad = \sum _{l = 0}^{n-1} \frac{1}{M^{n-l}} \operatorname {Var}\Big [ f\big ( X^{0,x,a}, V_l^0(X^{0,x,a}) \big ) - \mathbb {1}_{\mathbb {N}}(l) f\big ( X^{0,x,a}, V_{\max \{ l-1,0 \}}^1(X^{0, x ,a}) \big ) \Big ] \nonumber \\&\quad \le \sum _{l = 0}^{n-1} \frac{1}{M^{n-l}} {\mathbb {E}}\Big [ \big | f\big ( X^{0,x,a}, V_l^0(X^{0,x,a}) \big ) - \mathbb {1}_{\mathbb {N}}(l) f\big ( X^{0,x,a}, V_{\max \{ l-1,0 \}}^1(X^{0, x ,a}) \big ) \big |^2 \Big ]. \end{aligned}$$

Combining this, the fact that for all $(x, a) \in {\mathbb {X}}\times A$ it holds that $\big (V_0^0(x)\big )(a) = 0$, and the fact that for all $n \in {\mathbb {N}}$, $r_1, r_2, \dots , r_n \in [0,\infty )$ it holds that $\sqrt{r_1 + r_2 + \dots + r_n} \le \sqrt{r_1} + \sqrt{r_2} + \dots + \sqrt{r_n}$ proves that for all $n \in {\mathbb {N}}$, $(x,a) \in {\mathbb {X}}\times A$, $\theta \in \Theta$ it holds that

61 $$\begin{aligned}&\big ( \operatorname {Var}\big [\big (V_n^\theta (x)\big )(a) \big ] \big )^\frac{1}{2} \nonumber \\&\quad \le \sum _{l = 0}^{n-1} \frac{1}{\sqrt{M^{n-l}}} \Big ( {\mathbb {E}}\Big [ \big | f\big ( X^{0,x,a}, V_l^0(X^{0,x,a}) \big ) - \mathbb {1}_{\mathbb {N}}(l) f\big ( X^{0,x,a}, V_{\max \{ l-1, 0 \}}^1(X^{0,x,a}) \big ) \big |^2 \Big ] \Big )^\frac{1}{2} \nonumber \\&\quad = \sum _{l = 1}^{n-1} \frac{1}{\sqrt{M^{n-l}}} \Big ( {\mathbb {E}}\Big [ \big | f\big ( X^{0,x,a}, V_l^0(X^{0,x,a}) \big ) - \mathbb {1}_{\mathbb {N}}(l) f\big ( X^{0,x,a}, V_{l-1}^1(X^{0,x,a}) \big ) \big |^2 \Big ] \Big )^\frac{1}{2} \nonumber \\&\quad \quad + \frac{1}{\sqrt{M^n}} \Big ( {\mathbb {E}}\big [ |f( X^{0,x,a},0 )|^2 \big ] \Big )^\frac{1}{2}. \end{aligned}$$

The assumption that for all $x \in {\mathbb {X}}$, $r, s \in {\mathbb {R}}^A$ it holds that $| f(x,r) - f(x,s) | \le L \max _{a \in A} | r(a) - s(a) |$ and the triangle inequality imply that for all $n \in {\mathbb {N}}$, $(x,a) \in {\mathbb {X}}\times A$, $\theta \in \Theta$ it holds that

62 $$\begin{aligned}&\big ( \operatorname {Var}\big [ \big (V_n^\theta (x)\big )(a) \big ] \big )^\frac{1}{2} \nonumber \\  &\quad \le \frac{1}{\sqrt{M^n}} \Big ( {\mathbb {E}}\big [ |f( X^{0,x,a},0 )|^2 \big ] \Big )^\frac{1}{2} + \sum _{l = 1}^{n-1} \frac{L}{\sqrt{M^{n-l}}} \Big ( {\mathbb {E}}\Big [ \big \Vert V_l^0(X^{0,x,a}) - V_{l-1}^1(X^{0,x,a}) \big \Vert ^2_\infty \Big ] \Big )^\frac{1}{2} \nonumber \\  &\quad \le \sum _{l = 1}^{n-1} \frac{L}{\sqrt{M^{n-l}}} \bigg [ \big ( {\mathbb {E}}\big [ \Vert V_l^0(X^{0,x,a}) - v(X^{0,x,a}) \Vert _\infty ^2 \big ] \big )^\frac{1}{2} \nonumber \\&+ \big ( {\mathbb {E}}\big [ \Vert v(X^{0,x,a}) - V_{l-1}^1(X^{0,x,a}) \Vert _\infty ^2 \big ] \big )^\frac{1}{2} \bigg ] \nonumber \\  &\quad + \frac{1}{\sqrt{M^n}} \Big ( {\mathbb {E}}\big [ |f( X^{0,x,a},0 )|^2 \big ] \Big )^\frac{1}{2}. \end{aligned}$$

The fact that for all $l \in {\mathbb {N}}_0$, $(x,a) \in {\mathbb {X}}\times A$ it holds that $\Omega \ni \omega \mapsto V_l^0(X^{0,x,a}(\omega ),\omega ) \in {\mathbb {R}}^A$ and $\Omega \ni \omega \mapsto V_l^1(X^{0,x,a}(\omega ), \omega ) \in {\mathbb {R}}^A$ are identically distributed ensures for all $l \in {\mathbb {N}}_0$, $(x,a) \in {\mathbb {X}}\times A$ that $\Omega \ni \omega \mapsto \Vert v(X^{0,x,a}(\omega )) - V_l^0(X^{0,x,a}(\omega ), \omega ) \Vert ^2_\infty \in [0, \infty )$ and $\Omega \ni \omega \mapsto \Vert v(X^{0,x,a}(\omega )) - V_l^1(X^{0,x,a}(\omega ), \omega ) \Vert ^2_\infty \in [0, \infty )$ are identically distributed. This and (62) establish that for all $n \in {\mathbb {N}}$, $(x,a) \in {\mathbb {X}}\times A$, $\theta \in \Theta$ it holds that

63 $$\begin{aligned}&\big ( \operatorname {Var}\big [ \big (V_n^\theta (x)\big )(a) \big ] \big )^\frac{1}{2} \nonumber \\  &\quad \le \sum _{l = 1}^{n-1} \frac{L}{\sqrt{M^{n-l}}} \bigg [ \big ( {\mathbb {E}}\big [ \Vert V_l^0(X^{0,x,a}) - v(X^{0,x,a}) \Vert _\infty ^2 \big ] \big )^\frac{1}{2}\nonumber \\  &+ \big ( {\mathbb {E}}\big [ \Vert v(X^{0,x,a}) - V_{l-1}^0(X^{0,x,a}) \Vert _\infty ^2 \big ] \big )^\frac{1}{2} \bigg ] \nonumber \\  &\quad + \frac{1}{\sqrt{M^n}} \Big ( {\mathbb {E}}\big [ |f( X^{0,x,a},0 )|^2 \big ] \Big )^\frac{1}{2} \nonumber \\  &\quad = \frac{1}{\sqrt{M^n}} \Big ( {\mathbb {E}}\big [ |f( X^{0,x,a},0 )|^2 \big ] \Big )^\frac{1}{2} + \sum _{l = 1}^{n-1} \frac{L}{\sqrt{M^{n-l}}}\big ( {\mathbb {E}}\big [ \Vert V_l^0(X^{0,x,a}) - v(X^{0,x,a}) \Vert _\infty ^2 \big ] \big )^\frac{1}{2} \nonumber \\  &\quad \quad + \sum _{l = 0}^{n-2} \frac{L\sqrt{M}}{\sqrt{M^{n-l}}} \big ( {\mathbb {E}}\big [ \Vert V_l^0(X^{0,x,a}) - v(X^{0,x,a}) \Vert _\infty ^2 \big ] \big )^\frac{1}{2} \nonumber \\  &\quad = \frac{1}{\sqrt{M^n}} \Big ( {\mathbb {E}}\big [ |f( X^{0,x,a},0 )|^2 \big ] \Big )^\frac{1}{2} + \mathbb {1}_{[2, \infty )}(n) \frac{L}{\sqrt{M}} \big ( {\mathbb {E}}\big [ \Vert V_{n-1}^0(X^{0,x,a}) - v(X^{0,x,a}) \Vert ^2_\infty \big ] \big )^\frac{1}{2} \nonumber \\  &\quad + \mathbb {1}_{[2, \infty )}(n) \frac{L\sqrt{M}}{\sqrt{M^n}} \big ( {\mathbb {E}}\big [ \Vert v(X^{0,x,a}) \Vert ^2_\infty \big ] \big )^\frac{1}{2} \nonumber \\  &\quad + \sum _{l = 1}^{n - 2} \frac{L(1 + \sqrt{M})}{\sqrt{M^{n-l}}} \big ( {\mathbb {E}}\big [ \Vert V_l^0(X^{0,x,a}) - v(X^{0,x,a}) \Vert ^2_\infty \big )^\frac{1}{2}. \end{aligned}$$

The proof of Lemma 3.7 is thus complete. $\square$

### Non-recursive Error Bounds for MLFP Approximations

#### Proposition 3.8

Assume Setting 3.1, assume *A* is finite, let $c_f, c_v, c_{\mathfrak {w}}, L \in [0, \infty )$, let ${\mathfrak {w}}:{\mathbb {X}}\rightarrow (0, \infty )$ be ${\mathcal {X}} / {\mathcal {B}}((0,\infty ))$-measurable, let $v :{\mathbb {X}}\rightarrow {\mathbb {R}}^A$ be ${\mathcal {X}}/\widetilde{{\mathcal {A}}}$-measurable, let $c = \frac{3}{2} \max \big \{ \frac{c_v}{c_{\mathfrak {w}}}, c_vL + c_f, \frac{|A|c_f}{c_{\mathfrak {w}}|A|L + 1} \big \}$, assume that for all $(x,a) \in {\mathbb {X}}\times A$, $r,s \in {\mathbb {R}}^A$ it holds that $|f(x,r) - f(x,s)| \le L \max _{b \in A} |r(b) - s(b)|$,     $\big ( {\mathbb {E}}\big [ |f(X^{0,x,a}, 0)|^2 \big ] \big )^\frac{1}{2} \le c_f {\mathfrak {w}}(x)$,      $\big ( {\mathbb {E}}\big [ \Vert v(X^{0,x,a}) \Vert _\infty ^2 \big ] \big )^\frac{1}{2} \le c_v {\mathfrak {w}}(x)$, $\big ( {\mathbb {E}}\big [ \big |{\mathfrak {w}}(X^{0,x,a})\big |^2 \big ] \big )^\frac{1}{2} \le c_{\mathfrak {w}}{\mathfrak {w}}(x)$, and $\big (v(x)\big )(a) = {\mathbb {E}}\big [ f(X^{0,x,a}, v(X^{0,x,a})) \big ]$. Then it holds for all $n \in {\mathbb {N}}$, $x\in {\mathbb {X}}$, $\theta \in \Theta$ that64

#### Proof of Proposition 3.8

The triangle inequality, Lemma 3.4, and the assumption that *A* is finite ensure that for all $n \in {\mathbb {N}}$, $x\in {\mathbb {X}}$, $\theta \in \Theta$ it holds that

65 $$\begin{aligned}&\Big ( {\mathbb {E}}\big [ \Vert v(x) - V_n^\theta (x) \Vert _\infty ^2 \big ] \Big )^\frac{1}{2}\nonumber \\&\quad = \Big ( {\mathbb {E}}\Big [ \big \Vert v(x) - {\mathbb {E}}\big [ V_n^\theta (x) \big ] + {\mathbb {E}}\big [ V_n^\theta (x) \big ] - V_n^\theta (x)\big \Vert _\infty ^2 \Big ] \Big )^\frac{1}{2}\nonumber \\&\quad \le \Big ( {\mathbb {E}}\Big [ \big \Vert v(x) - {\mathbb {E}}\big [ V_n^\theta (x) \big ] \big \Vert _\infty ^2 \Big ] \Big )^\frac{1}{2} + \Big ( {\mathbb {E}}\Big [ \big \Vert V_n^\theta (x) - {\mathbb {E}}\big [ V_n^\theta (x) \big ] \big \Vert ^2_\infty \Big ] \Big )^\frac{1}{2}\nonumber \\&\quad \le \big \Vert v(x) - {\mathbb {E}}\big [ V_n^\theta (x) \big ] \big \Vert _\infty + \sum _{a \in A} \Big ( {\mathbb {E}}\Big [ \big | V_n^\theta (x)(a) - {\mathbb {E}}\big [ V_n^\theta (x)(a) \big ] \big |^2 \Big ] \Big )^\frac{1}{2}\nonumber \\&\quad = \big \Vert v(x) - {\mathbb {E}}\big [ V_n^\theta (x) \big ] \big \Vert _\infty + \sum _{a \in A} \big ( \operatorname {Var}[V_n^\theta (x)(a)] \big )^\frac{1}{2}. \end{aligned}$$

The assumption that $X^0 :{\mathbb {X}}\times \Omega \rightarrow {\mathbb {X}}^A$ is $({\mathcal {X}} \otimes {\mathbb {F}}^0)/\widetilde{{\mathcal {X}}}$-measurable, the assumption that the sigma-algebras ${\mathbb {F}}^\theta$, $\theta \in \Theta$, are independent, the assumption that *A* is finite, Lemma 3.2, and the disintegration theorem [33, Lemma 2.2] prove that for all $l \in {\mathbb {N}}$, $(x,a) \in {\mathbb {X}}\times A$ it holds that

66 $$\begin{aligned} {\mathbb {E}}\big [ \Vert V_l^0(X^{0,x,a}) - v(X^{0,x,a}) \Vert ^2_\infty \big ]&= \int _{\mathbb {X}}{\mathbb {E}}\big [ \Vert V_l^0(y) - v(y)\Vert ^2_\infty \big ] \big ( X^{0,x,a}({\mathbb {P}}) \big )(dy) \nonumber \\  &\le \bigg (\sup _{y \in {\mathbb {X}}} \frac{{\mathbb {E}}\big [ \Vert V_l^0(y) - v(y) \Vert ^2_\infty \big ]}{|{\mathfrak {w}}(y)|^2} \bigg ) c_{\mathfrak {w}}^2 |{\mathfrak {w}}(x)|^2. \end{aligned}$$

This, Lemma 3.6, Lemma 3.7, and (65) imply that for all $n \in {\mathbb {N}}$, $x \in {\mathbb {X}}$ it holds that

67 $$\begin{aligned} \begin{aligned}&\big ({\mathbb {E}}\big [ \Vert v(x) - V_n^0(x)\Vert ^2_\infty \big ]\big )^\frac{1}{2} \\  &\quad \le c_{\mathfrak {w}}{\mathfrak {w}}(x)L \bigg ( \sup _{y \in {\mathbb {X}}} \frac{{\mathbb {E}}\big [ \Vert v(y) - V_{n-1}^0(y) \Vert ^2_\infty \big ]}{|{\mathfrak {w}}(y)|^2} \bigg )^\frac{1}{2} + \sum _{a \in A} \bigg [ \frac{1}{\sqrt{M^n}} \big ( {\mathbb {E}}\big [ |f(X^{0,x,a}, 0)|^2 \big ] \big )^\frac{1}{2} \\  &\quad + \mathbb {1}_{[2,\infty )(n)} \frac{L\sqrt{M}}{\sqrt{M^n}} \big ( {\mathbb {E}}\big [ \Vert v(X^{0,x,a}) \Vert ^2_\infty \big ] \big )^\frac{1}{2} + \mathbb {1}_{[2,\infty )}(n) \frac{c_{\mathfrak {w}}{\mathfrak {w}}(x) L}{\sqrt{M}}\\&\quad \bigg ( \sup _{y \in {\mathbb {X}}} \frac{{\mathbb {E}}\big [ \Vert v(y) - V_{n-1}^0(y) \Vert ^2_\infty \big ]}{|{\mathfrak {w}}(y)|^2} \bigg )^\frac{1}{2} \\  &\quad \quad + \sum _{l = 1}^{n-2}\frac{c_{\mathfrak {w}}{\mathfrak {w}}(x) L(1 + \sqrt{M})}{\sqrt{M^{n-l}}} \bigg ( \sup _{y \in X} \frac{{\mathbb {E}}\big [ \Vert v(y) - V_l^0(y)\Vert ^2_\infty \big ]}{|{\mathfrak {w}}(y)|^2} \bigg )^\frac{1}{2}\bigg ] \\  &\quad \le c_{\mathfrak {w}}{\mathfrak {w}}(x) L \bigg (\sup _{y \in {\mathbb {X}}} \frac{{\mathbb {E}}\big [ \Vert v(y) - V_{n-1}^0(y) \Vert ^2_\infty \big ]}{|{\mathfrak {w}}(y)|^2} \bigg )^\frac{1}{2} + \frac{|A|{\mathfrak {w}}(x)}{\sqrt{M^n}} \big (c_f + \mathbb {1}_{[2,\infty )}(n) c_v L \sqrt{M}\big ) \\  &\quad \quad + \mathbb {1}_{[2,\infty )}(n) \frac{c_{\mathfrak {w}}|A|L {\mathfrak {w}}(x)}{\sqrt{M}} \bigg (\sup _{y \in {\mathbb {X}}} \frac{{\mathbb {E}}\big [ \Vert v(y) - V_{n-1}^0(y) \Vert ^2_\infty \big ]}{|{\mathfrak {w}}(y)|^2} \bigg )^\frac{1}{2}\\  &\quad \quad + c_{\mathfrak {w}}{\mathfrak {w}}(x) |A|L(1 + \sqrt{M}) \sum _{l = 1}^{n-2} \frac{1}{\sqrt{M^{n-l}}} \bigg ( \sup _{y \in {\mathbb {X}}} \frac{{\mathbb {E}}\big [ \Vert v(y) - V_l^0(y) \Vert ^2_\infty \big ]}{|{\mathfrak {w}}(y)|^2} \bigg )^\frac{1}{2}. \end{aligned} \end{aligned}$$

For all $n \in {\mathbb {N}}_0$ let

68 $$\begin{aligned} F_n = \bigg ( \sup _{x \in {\mathbb {X}}} \frac{{\mathbb {E}}\big [ \Vert v(x) - V_n^0(x) \Vert ^2_\infty \big ]}{|{\mathfrak {w}}(x)|^2} \bigg )^\frac{1}{2} \quad \text {and} \quad a_n&= M^\frac{n}{2} F_n. \end{aligned}$$

Lemma 3.4 ensures that for all $n \in {\mathbb {N}}_0$ it holds that $a_n, F_n \in [0, \infty )$. Note that (67) shows for all $n \in {\mathbb {N}}\cap [2, \infty )$ that

69 $$\begin{aligned} F_n&\le |A| M^{- \frac{n}{2}} \big ( c_f + c_vLM^\frac{1}{2}\big ) \nonumber \\&\quad + c_{\mathfrak {w}}L(1 + |A|M^{- \frac{1}{2}}) \bigg ( \sup _{y \in {\mathbb {X}}} \frac{{\mathbb {E}}\big [ \Vert v(y) - V_{n-1}^0(y) \Vert ^2_\infty \big ]}{|{\mathfrak {w}}(y)|^2} \bigg )^\frac{1}{2} \nonumber \\&\quad + c_{\mathfrak {w}}|A|L(1+ M^\frac{1}{2}) \sum _{l = 1}^{n-2} M^{-\frac{n-l}{2}} \bigg ( \sup _{y \in {\mathbb {X}}} \frac{{\mathbb {E}}\big [ \Vert v(y) - V_l^0(y) \Vert ^2_\infty \big ]}{|{\mathfrak {w}}(y)|^2} \bigg )^\frac{1}{2} \nonumber \\&= |A| M^{- \frac{n}{2}} \big ( c_f + c_vLM^\frac{1}{2}\big ) + c_{\mathfrak {w}}L(1 + |A|M^{- \frac{1}{2}})F_{n-1} \nonumber \\&\quad + c_{\mathfrak {w}}|A|L(1+ M^\frac{1}{2}) \sum _{l = 1}^{n-2} M^{-\frac{n-l}{2}} F_l. \end{aligned}$$

This implies for all $n \in {\mathbb {N}}\cap [2, \infty )$ that

70 $$\begin{aligned} \begin{aligned} a_n&\le |A|(c_f + c_vLM^\frac{1}{2}) + c_{\mathfrak {w}}M^\frac{1}{2}L(1 + |A|M^{-\frac{1}{2}}) M^\frac{n-1}{2}F_{n-1} \\  &+ c_{\mathfrak {w}}|A|L(1+ M^\frac{1}{2})\sum _{l = 1}^{n-2}M^\frac{l}{2}F_l \\  &= |A|(c_f + c_vLM^\frac{1}{2}) + c_{\mathfrak {w}}L(M^\frac{1}{2} + |A|)a_{n-1}\\  &+ c_{\mathfrak {w}}|A|L(1+M^\frac{1}{2})\sum _{l= 1}^{n-2} a_l. \end{aligned} \end{aligned}$$

Moreover it holds that

71 $$\begin{aligned} a_0 = F_0 = \bigg (\sup _{y \in {\mathbb {X}}} \frac{{\mathbb {E}}\big [ \Vert v(y) \Vert ^2_\infty \big ]}{|{\mathfrak {w}}(y)|^2} \bigg )^\frac{1}{2} = \sup _{(y,a) \in {\mathbb {X}}\times A} \frac{\left| \big (v(y)\big )(a) \right| }{|{\mathfrak {w}}(y)|}. \end{aligned}$$

The assumption that for all $(x,a) \in {\mathbb {X}}\times A$ it holds that $\big (v(x)\big )(a) = {\mathbb {E}} \big [ f(X^{0,x,a}, v(X^{0,x,a})) \big ]$, Jensen’s inequality, the triangle inequality, the assumption that for all $x \in {\mathbb {X}}$, $r,s \in {\mathbb {R}}^A$ it holds that $|f(x,r) - f(x,s)| \le L \max _{a \in A} |r(a) - s(a)|$, and the assumption that for all $(x,a) \in {\mathbb {X}}\times A$ it holds that $\big ( {\mathbb {E}}\big [ |f(X^{0,x,a}, 0|^2 \big ] \big )^\frac{1}{2} \le c_f {\mathfrak {w}}(x)$ and $\big ( {\mathbb {E}}\big [ \Vert v(X^{0,x,a}) \Vert _\infty ^2 \big )^\frac{1}{2} \le c_v {\mathfrak {w}}(x)$ prove for all $(x,a) \in {\mathbb {X}}\times A$ that

72 $$\begin{aligned} \frac{\big | \big (v(x)\big )(a) \big |}{|{\mathfrak {w}}(x)|}&= \frac{1}{{\mathfrak {w}}(x)} \big ( \big | {\mathbb {E}}\big [ f(X^{0,x,a}, v(X^{0,x,a})) \big ] \big |^2 \big )^\frac{1}{2} \nonumber \\&\le \frac{1}{{\mathfrak {w}}(x)} \big ( {\mathbb {E}}\big [ \big | f(X^{0,x,a}, v(X^{0,x,a}))\big |^2 \big ] \big )^\frac{1}{2} \nonumber \\&\le \frac{1}{{\mathfrak {w}}(x)} \Big [ L \big ({\mathbb {E}}\big [ \sup _{b \in A} |v(X^{0,x,a})(b)|^2 \big ] \big )^\frac{1}{2} \nonumber \\&\quad + \big ( {\mathbb {E}}\big [ |f(X^{0,x,a}, 0)|^2 \big ] \big )^\frac{1}{2} \Big ] \le c_vL + c_f. \end{aligned}$$

This and (71) ensure that $a_0 \le c_vL + c_f$. Note that (67) implies that $a_1 \le M^\frac{1}{2} \big ( c_{\mathfrak {w}}La_0 + |A|M^{-\frac{1}{2}}c_f \big ) \le c_{\mathfrak {w}}L M^\frac{1}{2}(c_vL + c_f) + |A|c_f$. Moreover (70) ensures $a_2 \le |A|(c_f + c_vLM^\frac{1}{2}) + c_{\mathfrak {w}}L(M^\frac{1}{2} + |A|)a_1$. Let $(\xi _n)_{n \in {\mathbb {N}}_0}$, $(b_n)_{n \in {\mathbb {N}}_0} \subseteq {\mathbb {R}}$ satisfy for all $n \in {\mathbb {N}}_0$ that

73 $$\begin{aligned} b_0&= \xi _0 = \max \Big \{ \frac{c_v}{c_{\mathfrak {w}}}, c_vL + c_f, \frac{|A|c_f}{c_{\mathfrak {w}}|A|L + 1} \Big \}, \quad b_1 = |A|c_f - (c_{\mathfrak {w}}|A|L + 1)b_0, \nonumber \\ b_2&= |A|LM^\frac{1}{2}(c_v - c_{\mathfrak {w}}b_0), \quad b_{n+3} = 0,\quad \xi _1 = c_{\mathfrak {w}}LM^\frac{1}{2}b_0 + |A|c_f, \nonumber \\ \xi _{n+2}&= |A|(c_f + c_vLM^\frac{1}{2}) + c_{\mathfrak {w}}L(M^\frac{1}{2} + |A|)\xi _{n+1} + c_{\mathfrak {w}}|A|L(1 + M^\frac{1}{2})\sum _{l = 1}^{n}\xi _l. \end{aligned}$$

Combining (67), (70), (73), and induction establishes for all $n \in {\mathbb {N}}_0$ that $a_n \le \xi _n$. Observe that (73) ensures that for all $n \in {\mathbb {N}}_0$ it holds that

74 $$\begin{aligned} \xi _0&= b_0, \quad \xi _1 = b_1 + (c_{\mathfrak {w}}L(M^\frac{1}{2} + |A|) + 1)\xi _0, \nonumber \\ \xi _{n + 2}&= b_{n+2} + (c_{\mathfrak {w}}L(M^\frac{1}{2} + |A|) + 1)\xi _{n+1} + c_{\mathfrak {w}}LM^\frac{1}{2}(|A| - 1)\xi _n. \end{aligned}$$

Let $$x_1, x_2 \in {\mathbb {R}}$$ satisfy

75 $$\begin{aligned} x_1&= \frac{c_{\mathfrak {w}}L (M^\frac{1}{2} + |A|) + 1 - \sqrt{\big ( c_{\mathfrak {w}}L (M^\frac{1}{2} + |A|) + 1 \big )^2 + 4c_{\mathfrak {w}}L M^\frac{1}{2} (|A| - 1) } }{2}, \nonumber \\ x_2&= \frac{c_{\mathfrak {w}}L (M^\frac{1}{2} + |A|) + 1 + \sqrt{\big ( c_{\mathfrak {w}}L (M^\frac{1}{2} + |A|) + 1 \big )^2 + 4c_{\mathfrak {w}}L M^\frac{1}{2} (|A| - 1) } }{2}. \end{aligned}$$

Note that for all $i \in \{ 1,2 \}$ it holds that $x_i^2 = \big (c_{\mathfrak {w}}L (M^\frac{1}{2} + |A|) + 1\big ) x_i + c_{\mathfrak {w}}L M^\frac{1}{2}(|A| - 1)$. Moreover note that the assumption that *A* is nonempty yields that $c_{\mathfrak {w}}L(M^\frac{1}{2} + |A|) + 1 \ge 1$ and $c_{\mathfrak {w}}LM^\frac{1}{2}(|A| - 1) \ge 0$. This ensures that $x_2 > 0 \ge x_1$. This and (75) imply that $|x_2| \ge |x_1|$. Hence it holds that

76 $$\begin{aligned} \frac{|x_2|}{|x_2 - x_1|} \le 1 \quad \text { and } \quad \frac{|x_1|}{|x_2 - x_1|} \le \frac{1}{2}. \end{aligned}$$

This, the fact that $|x_1| \le |x_2|$, and the triangle inequality prove that for all $n \in {\mathbb {N}}_0$ it holds that

77 $$\begin{aligned} \frac{x_2^{n+1} - x_1^{n+1}}{x_2 - x_1}&\le \frac{|x_2|^{n + 1}}{x_2 - x_1} + \frac{|x_1|^{n+1}}{x_2- x_1} \le \frac{x_2}{x_2 - x_1} x_2^n + \frac{|x_1|}{x_2 - x_1} x_2^n \le \frac{3}{2}x_2^n. \end{aligned}$$

The discrete Gronwall-type two-step recursion in [36, Lemma 2.1] (applied with $\kappa \curvearrowleft c_{\mathfrak {w}}L(M^\frac{1}{2} + |A|) + 1$, $\lambda \curvearrowleft c_{\mathfrak {w}}LM^\frac{1}{2}(|A| - 1)$, $(a_k)_{k \in {\mathbb {N}}_0} \curvearrowleft (\xi _k)_{k \in {\mathbb {N}}_0}$, $(b_k)_{k \in {\mathbb {N}}_0} \curvearrowleft (b_k)_{k \in {\mathbb {N}}_0}$, $x_{1/2} \curvearrowleft x_{1/2}$ in the notation of [36, Lemma 2.1]) demonstrates that for all $n \in {\mathbb {N}}_0$ it holds that

78 $$\begin{aligned} \xi _n = \frac{1}{x_2 - x_1} \Big ( b_0 (x_2^{n+1} - x_1^{n+1}) + b_1(x_2^n - x_1^n) + b_2(x_2^{ \max \{ n-1, 0\} } - x_1^{ \max \{ n-1, 0 \} } ) \Big ). \end{aligned}$$

Note that (73) ensures that $b_1 \le 0$ and $b_2 \le 0$. This, the fact that $x_2 > x_1$, and the fact that for all $n \in {\mathbb {N}}_0$ it holds that $a_n \le \xi _n$ proves that for all $n \in {\mathbb {N}}_0$ it holds that

79 $$\begin{aligned} a_n \le b_0\frac{x_2^{n+1} - x_1^{n+1}}{x_2 - x_1}. \end{aligned}$$

Combining this and (77) implies that for all $n \in {\mathbb {N}}_0$ it holds that

80 $$\begin{aligned} F_n \le \frac{3}{2}b_0 \big ( M^{-\frac{1}{2}} x_2 \big )^n. \end{aligned}$$

Furthermore, observe that

81 $$\begin{aligned}&M^{-\frac{1}{2}}x_2 = M^{-\frac{1}{2}} \Bigg ( \frac{ c_{\mathfrak {w}}L(M^\frac{1}{2} + |A|) + 1 + \sqrt{\big ( c_{\mathfrak {w}}L(M^\frac{1}{2} + |A|) + 1 \big )^2 + 4c_{\mathfrak {w}}LM^\frac{1}{2}(|A| - 1) } }{2} \Bigg ) \nonumber \\&= \frac{ c_{\mathfrak {w}}L(1 + |A|M^{-\frac{1}{2}}) + M^{-\frac{1}{2}} + \sqrt{\big ( c_{\mathfrak {w}}L(1 + |A|M^{-\frac{1}{2}}) + M^{-\frac{1}{2}} \big )^2 + 4c_{\mathfrak {w}}LM^{-\frac{1}{2}}(|A| - 1)} }{2}. \end{aligned}$$

This, (68), and (80) prove that for all $x \in {\mathbb {X}}$, $n \in {\mathbb {N}}$ it holds that

82 $$\begin{aligned}&\bigg ( \frac{{\mathbb {E}}\big [ \Vert v(x) - V_n^\theta (x) \Vert _\infty ^2 \big ]}{|{\mathfrak {w}}(x)|^2} \bigg )^\frac{1}{2} \nonumber \\&\quad \le c \Bigg ( \frac{c_{\mathfrak {w}}L(1 + |A|M^{-\frac{1}{2}}) + M^{-\frac{1}{2}} + \sqrt{\big ( c_{\mathfrak {w}}L(1 + |A|M^{-\frac{1}{2}}) + M^{-\frac{1}{2}}\big )^2 + 4c_{\mathfrak {w}}LM^{-\frac{1}{2}}(|A| - 1)} }{2} \Bigg )^n. \end{aligned}$$

The proof of Proposition 3.8 is thus complete. $\square$

#### Corollary 3.9

Assume Setting 3.1, assume *A* is finite, let $c_f, c_v, c_{\mathfrak {w}}, L \in [0, \infty )$ with $c_{\mathfrak {w}}L < 1$, let $M > (1 + c_{\mathfrak {w}}L(2|A| - 1))^2(1-c_{\mathfrak {w}}L)^{-2}$, let ${\mathfrak {w}}:{\mathbb {X}}\rightarrow (0, \infty )$ be ${\mathcal {X}}/{\mathcal {B}}((0, \infty ))$-measurable, let $v :{\mathbb {X}}\rightarrow {\mathbb {R}}^A$ be ${\mathcal {X}} / \widetilde{{\mathcal {A}}}$-measurable, assume for all $(x,a) \in {\mathbb {X}}\times A$, $r,s \in {\mathbb {R}}^A$ that $|f(x,r) - f(x,s)| \le L \max _{b \in A} |r(b) - s(b)|$, $\big ( {\mathbb {E}}\big [ |f(X^{0,x,a}, 0)|^2 \big ] \big )^\frac{1}{2} \le c_f {\mathfrak {w}}(x)$, $\big ( {\mathbb {E}}\big [ \Vert v(X^{0,x,a}) \Vert _\infty ^2 \big ] \big )^\frac{1}{2} \le c_v {\mathfrak {w}}(x)$, $\big ( {\mathbb {E}}\big [ \big | {\mathfrak {w}}(X^{0,x,a}) \big |^2 \big ] \big )^\frac{1}{2} \le c_{\mathfrak {w}}{\mathfrak {w}}(x)$, and $\big (v(x)\big )(a) = {\mathbb {E}}\big [ f\big ( X^{0,x,a}, v(X^{0,x,a}) \big ) \big ]$. Then it holds that

83 $$\begin{aligned} \limsup _{n \rightarrow \infty } \bigg [ \sup _{x \in {\mathbb {X}}} \bigg ( \frac{ {\mathbb {E}}\big [ \Vert v(x) - V_n^0(x) \Vert ^2_\infty \big ] }{|{\mathfrak {w}}(x)|^2} \bigg )^\frac{1}{2} \bigg ] = 0. \end{aligned}$$

#### Proof of Corollary 3.9

Let $c \in [0, \infty )$ satisfy $c = \frac{3}{2} \max \big \{ \frac{c_v}{c_{\mathfrak {w}}}, c_vL + c_f, \frac{|A|c_f}{c_{\mathfrak {w}}|A|L + 1} \big \}$. Proposition 3.8 establishes that for all $n \in {\mathbb {N}}$, $x \in {\mathbb {X}}$ it holds that

84 $$\begin{aligned}&\bigg ( \frac{{\mathbb {E}}\big [ \Vert v(x) - V_n^0(x)\Vert ^2_\infty \big ]}{|{\mathfrak {w}}(x)|^2} \bigg )^\frac{1}{2} \nonumber \\&\quad \le c \bigg ( \frac{c_{\mathfrak {w}}L(1 + |A|M^{-\frac{1}{2}}) + M^{-\frac{1}{2}} + \sqrt{\big (c_{\mathfrak {w}}L(1 + |A|M^{-\frac{1}{2}}) + M^{-\frac{1}{2}}\big )^2 + 4c_{\mathfrak {w}}LM^{-\frac{1}{2}}(|A| - 1)} }{2} \Bigg )^n. \end{aligned}$$

Observe that the assumption that $M > (1 + c_{\mathfrak {w}}L(2|A| - 1))^2(1- c_{\mathfrak {w}}L)^{-2}$ implies $M^{-\frac{1}{2}} < (1- c_{\mathfrak {w}}L)(1 + c_{\mathfrak {w}}L(2|A| - 1))^{-1}$. This proves that

85 $$\begin{aligned}&c_{\mathfrak {w}}L(|A| - 1)M^{-\frac{1}{2}}\nonumber \\&\quad< \frac{c_{\mathfrak {w}}L(|A| - 1)(1- c_{\mathfrak {w}}L)}{1 + c_{\mathfrak {w}}L(2|A| - 1)} \nonumber \\&\quad = 1 - \Big ( c_{\mathfrak {w}}L + (1 - c_{\mathfrak {w}}L) - \frac{c_{\mathfrak {w}}L (|A| - 1) (1- c_{\mathfrak {w}}L)}{1 + c_{\mathfrak {w}}L(2 |A| - 1)} \Big ) \nonumber \\&\quad = 1 - \Big ( c_{\mathfrak {w}}L + \big ( 1 + c_{\mathfrak {w}}L (2 |A| - 1) - c_{\mathfrak {w}}L (|A| - 1) \big ) \frac{1 - c_{\mathfrak {w}}L}{1 + c_{\mathfrak {w}}L (2|A| - 1)} \Big ) \nonumber \\&\quad = 1 - \Big ( c_{\mathfrak {w}}L + \big ( 1 + c_{\mathfrak {w}}|A|L \big ) \frac{1 - c_{\mathfrak {w}}L}{1 + c_{\mathfrak {w}}L (2|A| - 1)} \Big ) \nonumber \\&\quad < 1 - \big ( c_{\mathfrak {w}}L + (1 + c_{\mathfrak {w}}|A|L)M^{-\frac{1}{2}} \big ) = 1 - \big (c_{\mathfrak {w}}L(1 + |A|M^{-\frac{1}{2}}) + M^{-\frac{1}{2}} \big ). \end{aligned}$$

The assumption that *A* is nonempty ensures that $|A| \le 2|A| - 1$. This and the assumption that $M > (1 + c_{\mathfrak {w}}L(2|A| - 1))^2(1-c_{\mathfrak {w}}L)^{-2}$ yield $M > (1 + c_{\mathfrak {w}}L|A|)^2(1-c_{\mathfrak {w}}L)^{-2}$. Therefore it holds that $c_{\mathfrak {w}}L(1 + |A|M^{-\frac{1}{2}}) + M^{-\frac{1}{2}} = c_{\mathfrak {w}}L + (1 + c_{\mathfrak {w}}|A|L)M^{-\frac{1}{2}}< 1 < 2$. This and (85) establish that

86 $$\begin{aligned}&\tfrac{c_{\mathfrak {w}}L(1 + |A|M^{-\frac{1}{2}}) + M^{-\frac{1}{2}} + \sqrt{\big ( c_{\mathfrak {w}}L(1 + |A|M^{-\frac{1}{2}}) + M^{-\frac{1}{2}}\big )^2 + 4c_{\mathfrak {w}}LM^{-\frac{1}{2}}(|A| - 1)} }{2} \nonumber \\&\quad < \tfrac{c_{\mathfrak {w}}L(1 + |A|M^{-\frac{1}{2}}) + M^{-\frac{1}{2}} + \sqrt{\big (c_{\mathfrak {w}}L(1 + |A|M^{-\frac{1}{2}}) + M^{-\frac{1}{2}}\big )^2 + 4 - 4\big (c_{\mathfrak {w}}L(1 + |A|M^{-\frac{1}{2}}) + M^{-\frac{1}{2}} \big ) } }{2} \nonumber \\&\quad = \tfrac{c_{\mathfrak {w}}L(1 + |A|M^{-\frac{1}{2}}) + M^{-\frac{1}{2}} + \sqrt{\big (2 - c_{\mathfrak {w}}L(1 + |A|M^{-\frac{1}{2}}) + M^{-\frac{1}{2}}\big )^2 } }{2} = 1. \end{aligned}$$

Combining (84) and (86) implies that

87 $$\begin{aligned} \limsup _{n \rightarrow \infty } \bigg [ \sup _{x \in {\mathbb {X}}} \bigg ( \frac{ {\mathbb {E}}\big [ \Vert v(x) - V_n^0(x) \Vert ^2_\infty \big ] }{|{\mathfrak {w}}(x)|^2} \bigg )^\frac{1}{2} \bigg ] = 0. \end{aligned}$$

The proof of Corollary 3.9 is thus complete. $\square$

## Computational Complexity Analysis for MLFP Approximations

In this section we combine the error analysis for the proposed MLFP approximations in Proposition 3.8 in Sect. 3.5 with a computational cost analysis for the proposed MLFP approximation schemes to obtain in Theorem 4.1 in Sect. 4.1 below as the main result of this article a complexity analysis for the proposed MLFP approximation schemes. Corollary 4.2 in Sect. 4.2 below specializes Theorem 4.1 to the specific situation of a functional fixed-point equation taking the form of a Bellman equation associated to optimal stochastic control problems. Corollary 4.3 in Sect. 4.3 below specializes Corollary 4.2 to the specific situation of a functional fixed-point equation taking the form of a Bellman equation associated to optimal stopping problems. Theorem 1.1 in the introduction, in turn, is an immediate consequence of Corollary 4.2 in Sect. 4.2.

### MLFP Approximations for Functional Fixed-Point Equations

#### Theorem 4.1

Let $M \in {\mathbb {N}}$, let $\Theta = \bigcup _{n \in {\mathbb {N}}} {\mathbb {Z}}^n$, let *A* be a finite nonempty set, let $\kappa \in [0,\infty )$, let ${\mathfrak {D}}$ be a nonempty set, let $\lambda _d, L_d, {\mathfrak {R}}_d\in [0,\infty )$, $d\in {\mathfrak {D}}$, let $(\Omega , {\mathcal {F}}, {\mathbb {P}})$ be a probability space, let $({\mathbb {X}}_d, {\mathcal {X}}_d)$, $d\in {\mathfrak {D}}$, be nonempty measurable spaces, let $\left\| \cdot \right\| _\infty :{\mathbb {R}}^A \rightarrow [0,\infty )$ satisfy for all $r \in {\mathbb {R}}^A$ that $\left\| r \right\| _\infty = \max _{a \in A} \left| r(a) \right|$, for every $d\in {\mathfrak {D}}$ let ${\mathfrak {w}}_d:{\mathbb {X}}_d\rightarrow (0,\infty )$ be ${\mathcal {X}}_d/{\mathcal {B}}((0,\infty ))$-measurable, for every $d\in {\mathfrak {D}}$ let $f_d:{\mathbb {X}}_d\times {\mathbb {R}}^A \rightarrow {\mathbb {R}}$ be $({\mathcal {X}}_d\otimes (\bigotimes _{a \in A}{\mathcal {B}}({\mathbb {R}})) )/{\mathcal {B}}({\mathbb {R}})$-measurable, for every $d\in {\mathfrak {D}}$ let ${\mathbb {F}}_d^\theta \subseteq {\mathcal {F}}$, $\theta \in \Theta$, be independent sub-sigma-algebras of ${\mathcal {F}}$, for every $d\in {\mathfrak {D}}$ let $X_d^\theta :{\mathbb {X}}_d\times \Omega \rightarrow {\mathbb {X}}_d^A$, $\theta \in \Theta$, be i.i.d. random fields which satisfy for all $d\in {\mathfrak {D}}$, $\theta \in \Theta$ that $X_d^\theta$ is $({\mathcal {X}}_d\otimes {\mathbb {F}}_d^\theta )/(\bigotimes _{a \in A} {\mathcal {X}}_d)$-measurable, assume for all $d\in {\mathfrak {D}}$, $(x,a) \in {\mathbb {X}}_d\times A$, $r,s \in {\mathbb {R}}^A$ that $|f_d(x,r) - f_d(x,s)| \le L_d\max _{b \in A} |r(b) - s(b)|$, $\left( {\mathbb {E}}\big [ |f_d(X_d^{0,x,a}, 0)|^2 \big ]\right) ^\frac{1}{2} \le \kappa {\mathfrak {w}}_d(x)$, $\left( {\mathbb {E}}\big [ | {\mathfrak {w}}_d(X_d^{0,x,a})|^2 \big ]\right) ^\frac{1}{2} \le \lambda _d{\mathfrak {w}}_d(x)$, and $\sup _{u \in {\mathfrak {D}}} \lambda _u L_u < 1$, assume

88 $$\begin{aligned} M > \frac{( 1 + (\sup _{d\in {\mathfrak {D}}} \lambda _dL_d)(2|A| - 1) )^2}{ (1 - (\sup _{d\in {\mathfrak {D}}} \lambda _dL_d))^2 }, \end{aligned}$$

for every $d\in {\mathfrak {D}}$ let $V_{n,d}^\theta :{\mathbb {X}}_d\times \Omega \rightarrow {\mathbb {R}}^A$, $n \in {\mathbb {N}}_0$, $\theta \in \Theta$, satisfy for all $n \in {\mathbb {N}}_0$, $(x,a) \in {\mathbb {X}}_d\times A$, $\theta \in \Theta$ that

89 $$\begin{aligned} \begin{aligned} \big (V_{n, d}^\theta (x)\big )(a)&= \sum _{l = 0}^{n-1} \frac{1}{M^{n-l}} \sum _{i = 1}^{M^{n-l}} f_d\big ( X_d^{(\theta , l, i),x ,a}, V_{l, d}^{(\theta , l, i)}(X_d^{(\theta , l, i), x, a}) \big ) \\&\quad -\mathbb {1}_{{\mathbb {N}}}(l) f_d\big ( X_d^{(\theta , l, i), x, a}, V_{\max \{ l-1, 0\}, d}^{(\theta , -l, i)}(X_d^{(\theta , l, i), x, a}) \big ), \end{aligned} \end{aligned}$$

and let ${\mathfrak {C}}_{n,d} \in [0, \infty )$, $n \in {\mathbb {N}}_0$, $d\in {\mathfrak {D}}$, satisfy for all $n \in {\mathbb {N}}_0$, $d\in {\mathfrak {D}}$ that

90 $$\begin{aligned} {\mathfrak {C}}_{n, d} \le \sum _{l = 0}^{n-1} M^{n-l}\big ( {\mathfrak {R}}_d+ |A|{\mathfrak {C}}_{l, d} + \mathbb {1}_{{\mathbb {N}}}(l) |A| {\mathfrak {C}}_{\max \{l-1, 0\}, d} \big ). \end{aligned}$$

Then the following holds:

(i) For every $d\in {\mathfrak {D}}$ there exists a unique function $v_d:{\mathbb {X}}_d\rightarrow {\mathbb {R}}^A$ which is ${\mathcal {X}}_d/ (\bigotimes _{a \in A} {\mathcal {B}}({\mathbb {R}}))$-measurable and satisfies for all   $(x,a) \in {\mathbb {X}}_d\times A$   that    $\sup _{y \in {\mathbb {X}}_d} \frac{\Vert v_d(y) \Vert _\infty }{{\mathfrak {w}}_d(y)} < \infty$, ${\mathbb {E}}\big [ |f_d(X_d^{0,x,a}, v_d(X_d^{0,x,a}))| \big ] < \infty$, and
  91 $$\begin{aligned} \big (v_d(x)\big )(a) = {\mathbb {E}}\big [ f_d(X_d^{0,x,a}, v_d(X_d^{0,x,a})) \big ]. \end{aligned}$$
(ii) There exist $N :(0, 1] \rightarrow {\mathbb {N}}$ and $c \in {\mathbb {R}}$ such that for all $d\in {\mathfrak {D}}$, $\varepsilon \in (0,1]$ it holds that ${\mathfrak {C}}_{N_\varepsilon , d} \le c {\mathfrak {R}}_d\varepsilon ^{-c}$ and
  92 $$\begin{aligned} \sup _{x \in {\mathbb {X}}_d} \bigg ( \frac{{\mathbb {E}}\big [ \Vert v_d(x) - V_{N_\varepsilon , d}^0(x) \Vert ^2_\infty \big ]}{|{\mathfrak {w}}_d(x)|^2} \bigg )^\frac{1}{2} \le \varepsilon . \end{aligned}$$

#### Proof of Theorem 4.1

Throughout this proof let $\alpha , \beta , \gamma \in {\mathbb {R}}\cup \{\infty \}$ satisfy

93 $$\begin{aligned} \alpha&= \frac{1}{2} \sup _{d\in {\mathfrak {D}}} \Big [ \lambda _dL_d(1 + |A|M^{-\frac{1}{2}}) + M^{-\frac{1}{2}} \nonumber \\&\quad + \sqrt{\big ( \lambda _dL_d(1 + |A|M^{-\frac{1}{2}}) + M^{-\frac{1}{2}} \big )^2 + 4M^{-\frac{1}{2}} \lambda _dL_d(|A| - 1) } \Big ], \nonumber \\ \beta&= \frac{\ln (3|A| M)}{\ln (\alpha ^{-1})}, \quad \text {and } \quad \gamma = \frac{3}{2} \sup _{d\in {\mathfrak {D}}} \left( \max \Big \{ \frac{\kappa }{1 - \lambda _dL_d}, \frac{|A|\kappa }{\lambda _d|A|L_d+ 1} \Big \}\right) , \end{aligned}$$

for every $d\in {\mathfrak {D}}$ let ${\mathfrak {B}}_{n, d} \in [0, \infty )$, $n \in {\mathbb {N}}_0$, satisfy for all $n \in {\mathbb {N}}_0$ that ${\mathfrak {B}}_{n, d} = |A|^{-n} {\mathfrak {C}}_{n, d}$, and let $N :(0,1] \rightarrow {\mathbb {N}}\cup \{ \infty \}$ satisfy for all $\varepsilon \in (0,1]$ that $N_\varepsilon = \min (\{ n \in {\mathbb {N}}: \gamma \alpha ^n \le \varepsilon \} \cup \{ \infty \})$. The assumption that $\sup _{d\in {\mathfrak {D}}} \lambda _dL_d< 1$ ensures that $\gamma \in [0, \infty )$. The triangle inequality yields

94 $$\begin{aligned} \alpha&= \frac{1}{2} \sup _{d\in {\mathfrak {D}}} \Big [ \lambda _dL_d(1 + |A|M^{-\frac{1}{2}}) + M^{-\frac{1}{2}} \nonumber \\&\quad + \sqrt{\big ( \lambda _dL_d(1 + |A|M^{-\frac{1}{2}}) + M^{-\frac{1}{2}} \big )^2 + 4M^{-\frac{1}{2}} \lambda _dL_d(|A| - 1) } \Big ], \nonumber \\&\le \frac{1}{2} \bigg [ (\sup _{d\in {\mathfrak {D}}} \lambda _dL_d)(1 + |A|M^{-\frac{1}{2}}) + M^{-\frac{1}{2}} \nonumber \\&\quad + \sqrt{\big ( ( \sup _{d\in {\mathfrak {D}}} \lambda _dL_d)(1 + |A|M^{-\frac{1}{2}}) + M^{-\frac{1}{2}} \big )^2 + 4M^{- \frac{1}{2}} (\sup _{d\in {\mathfrak {D}}}\lambda _dL_d)(|A| - 1) } \bigg ]. \end{aligned}$$

The assumption that $M^{-\frac{1}{2}} < \frac{1 - (\sup _{d\in {\mathfrak {D}}} \lambda _dL_d)}{1 + (\sup _{d\in {\mathfrak {D}}} \lambda _dL_d)(2|A| - 1)}$ ensures that

95 $$\begin{aligned}&1 - \big ( (\sup _{d\in {\mathfrak {D}}} \lambda _dL_d) (1 + |A|M^{-\frac{1}{2}}) + M^{-\frac{1}{2}} \big ) \nonumber \\&\quad = 1 - (\sup _{d\in {\mathfrak {D}}} \lambda _dL_d) - (1 + |A| (\sup _{d\in {\mathfrak {D}}} \lambda _dL_d)) M^{-\frac{1}{2}} \nonumber \\&\quad> (1 - (\sup _{d\in {\mathfrak {D}}} \lambda _dL_d)) - (1 + |A| (\sup _{d\in {\mathfrak {D}}} \lambda _dL_d)) \frac{1 - (\sup _{d\in {\mathfrak {D}}} \lambda _dL_d)}{1 + (\sup _{d\in {\mathfrak {D}}} \lambda _dL_d)(2|A| - 1)} \nonumber \\&\quad = \Big ( 1 + (\sup _{d\in {\mathfrak {D}}} \lambda _dL_d)(2 |A| - 1) - \big ( |A|(\sup _{d\in {\mathfrak {D}}} \lambda _dL_d) + 1 \big ) \Big ) \nonumber \\&\quad \quad \frac{1 - (\sup _{d\in {\mathfrak {D}}} \lambda _dL_d)}{1 + (\sup _{d\in {\mathfrak {D}}} \lambda _dL_d)(2|A| - 1)} \nonumber \\&\quad = \Big ( (\sup _{d\in {\mathfrak {D}}} \lambda _dL_d) (|A| - 1)\Big ) \frac{1 - (\sup _{d\in {\mathfrak {D}}} \lambda _dL_d)}{1 + (\sup _{d\in {\mathfrak {D}}} \lambda _dL_d)(2|A| - 1)} \nonumber \\&\quad > (\sup _{d\in {\mathfrak {D}}} \lambda _dL_d) (|A| - 1) M^{-\frac{1}{2}}. \end{aligned}$$

The assumption that *A* is nonempty implies that $|A| \le 2|A| - 1$. Hence in holds that $M > \frac{(1 + (\sup _{d\in {\mathfrak {D}}} \lambda _dL_d)(2|A| - 1) )^2}{(1 - (\sup _{d\in {\mathfrak {D}}}\lambda _dL_d))^2 } \ge \frac{(1 + |A|(\sup _{t \in T}\lambda _dL_d)^2}{(1 - (\sup _{d\in {\mathfrak {D}}} \lambda _dL_d))^2}$. This establishes that

96 $$\begin{aligned} (\sup _{d\in {\mathfrak {D}}} \lambda _dL_d) + (|A|(\sup _{d\in {\mathfrak {D}}} \lambda _dL_d) + 1) M^{-\frac{1}{2}} < 1. \end{aligned}$$

Combining this, (94), and (95) demonstrates that

97 $$\begin{aligned} \alpha&\le \frac{1}{2} \bigg [ (\sup _{d\in {\mathfrak {D}}} \lambda _dL_d) (1 + |A|M^{-\frac{1}{2}}) + M^{-\frac{1}{2}} \nonumber \\&\quad + \sqrt{\big ( (\sup _{d\in {\mathfrak {D}}} \lambda _dL_d) (1 + |A|M^{-\frac{1}{2}}) + M^{-\frac{1}{2}} \big )^2 + 4M^{- \frac{1}{2}} (\sup _{d\in {\mathfrak {D}}}\lambda _dL_d)( |A| - 1) } \bigg ] \nonumber \\&< \frac{1}{2} \bigg [ (\sup _{d\in {\mathfrak {D}}} \lambda _dL_d) (1 + |A|M^{-\frac{1}{2}}) + M^{-\frac{1}{2}} \nonumber \\&\quad + \sqrt{\big ( (\sup _{d\in {\mathfrak {D}}} \lambda _dL_d) (1 + |A|M^{-\frac{1}{2}}) + M^{-\frac{1}{2}} \big )^2 + 4\big ( 1 - (\sup _{d\in {\mathfrak {D}}} \big (\lambda _dL_d)(1 + |A|M^{-\frac{1}{2}}) + M^{-\frac{1}{2}} \big ) \big ) } \bigg ] \nonumber \\&= \frac{1}{2} \bigg [ (\sup _{d\in {\mathfrak {D}}} \lambda _dL_d) (1 + |A|M^{-\frac{1}{2}}) + M^{-\frac{1}{2}} \nonumber \\&\quad + \sqrt{\big (2 - \big ( (\sup _{d\in {\mathfrak {D}}} \lambda _dL_d) (1 + |A|M^{-\frac{1}{2}}) + M^{-\frac{1}{2}} \big ) \big )^2 } \bigg ] \nonumber \\&= 1. \end{aligned}$$

This and (93) ensure that $\alpha \in [M^{-\frac{1}{2}}, 1)$. The assumption that $M > \frac{(1 + (\sup _{d\in {\mathfrak {D}}} \lambda _dL_d) (2 |A| - 1) )^2}{(1- (\sup _{d\in {\mathfrak {D}}}\lambda _dL_d))^2}$ implies that $M > 1$. Hence it holds that $[M^{- \frac{1}{2}}, 1) \ne \emptyset$. The fact that $\alpha \in (0,1)$ demonstrates that for all $\varepsilon \in (0,1]$ it holds that $N_\varepsilon \in {\mathbb {N}}$. The fact that $\alpha \in [M^{-\frac{1}{2}}, 1)$ and the fact that $M > 1$ ensure that $\beta \in (2, \infty )$. Corollary 2.3 yields that for all $d\in {\mathfrak {D}}$ there exists a unique function $v_d:{\mathbb {X}}_d\rightarrow {\mathbb {R}}^A$ which is ${\mathcal {X}}_d/ (\bigotimes _{a \in A} {\mathcal {B}}({\mathbb {R}}))$-measurable and satisfies for all $d\in {\mathfrak {D}}$, $(x,a) \in {\mathbb {X}}_d\times A$ that $\sup _{y \in {\mathbb {X}}_d} \frac{\Vert v_d(y)\Vert _\infty }{{\mathfrak {w}}_d(y)} < \infty$, ${\mathbb {E}}\big [ |f_d(X_d^{0,x,a} v_d(X_d^{0,x,a}))| \big ] < \infty$, and $v_d(x,a) = {\mathbb {E}}\big [ f_d(X_d^{0,x,a}, v_d(X_d^{0,x,a})) \big ]$. This proves item (i). Lemma 2.4 implies for all $d\in {\mathfrak {D}}$, $(x,a) \in {\mathbb {X}}_d\times A$ that $\big ( {\mathbb {E}}\big [ \Vert v_d(X_d^{0,x,a}) \Vert _\infty ^2\big ]\big )^\frac{1}{2} \le \frac{\kappa \lambda _d}{1 - \lambda _dL_d} {\mathfrak {w}}_d(x)$. Proposition 3.8 establishes for all $n \in {\mathbb {N}}$, $d\in {\mathfrak {D}}$, $x \in {\mathbb {X}}_d$ that

98 $$\begin{aligned}&\bigg (\frac{{\mathbb {E}}\big [ \Vert v_d(x) - V_{n,d}^0(x) \Vert _\infty ^2 \big ]}{|{\mathfrak {w}}_d(x)|^2} \bigg )^\frac{1}{2} \nonumber \\&\quad \le \gamma \left( \tfrac{ \lambda _dL_d(1 + |A|M^{-\frac{1}{2}}) + M^{-\frac{1}{2}} + \sqrt{(\lambda _dL_d(1 + |A|M^{-\frac{1}{2}}) + M^{-\frac{1}{2}})^2 + 4\lambda _dL_dM^{-\frac{1}{2}} (|A| - 1) }}{2} \right) ^n \hspace{-0.3cm}. \end{aligned}$$

This demonstrates for all $n \in {\mathbb {N}}$, $d\in {\mathfrak {D}}$ that

99 $$\begin{aligned} \sup _{x \in {\mathbb {X}}_d} \bigg ( \frac{{\mathbb {E}}\big [ \Vert v_d(x) - V_{n,d}^0(x) \Vert _\infty ^2 \big ]}{|{\mathfrak {w}}_d(x)|^2} \bigg )^\frac{1}{2} \le \gamma \alpha ^n. \end{aligned}$$

Next, note that the fact that for all $n \in {\mathbb {N}}_0$, $d\in {\mathfrak {D}}$ it holds that ${\mathfrak {B}}_{n, d} = |A|^{-n} {\mathfrak {C}}_{n, d}$ and the assumption that for all $n \in {\mathbb {N}}_0$, $d\in {\mathfrak {D}}$ it holds that

100 $$\begin{aligned} {\mathfrak {C}}_{n, d} \le \sum _{l = 0}^{n-1} M^{n-l}\big ( {\mathfrak {R}}_d+ |A|{\mathfrak {C}}_{l, d} + \mathbb {1}_{{\mathbb {N}}}(l) |A| {\mathfrak {C}}_{\max \{l-1, 0\}, d} \big ) \end{aligned}$$

imply for all $n \in {\mathbb {N}}_0$, $d\in {\mathfrak {D}}$ that

101 $$\begin{aligned} \begin{aligned} {\mathfrak {B}}_{n, d}&\le |A|^{-n} \sum _{l = 0}^{n-1} M^{n-l}\big ( {\mathfrak {R}}_d+ |A|{\mathfrak {C}}_{l, d} + \mathbb {1}_{{\mathbb {N}}}(l) |A| {\mathfrak {C}}_{\max \{l-1, 0\}, d} \big ) \\&= \sum _{l = 0}^{n - 1} M^{n - l} \big ( |A|^{-n} {\mathfrak {R}}_d+ |A|^{1 - n + l} {\mathfrak {B}}_{l, d} + \mathbb {1}_{{\mathbb {N}}}(l) |A|^{1 - n + \max \{ l - 1, 0 \}} {\mathfrak {B}}_{\max \{l - 1, 0\}, d} \big ). \end{aligned} \end{aligned}$$

Moreover, observe that the assumption that *A* is nonempty implies that for all $n \in {\mathbb {N}}$, $l \in {\mathbb {N}}_0 \cap [0, n - 1]$ it holds that $\max \! \big \{ |A|^{-n}, |A|^{1 - n + l}, |A|^{1 - n + \max \{l - 1,0\}} \big \} \le 1$. Combining this, the fact that for all $d\in {\mathfrak {D}}$ it holds that ${\mathfrak {B}}_{0, d} = |A|^0 {\mathfrak {C}}_{0, d} = 0$, and (101) assures for all $n \in {\mathbb {N}}_0$, $d\in {\mathfrak {D}}$ that

102 $$\begin{aligned} {\mathfrak {B}}_{n, d} \le \sum _{l = 0}^{n - 1} M^{n - l} \big ( {\mathfrak {R}}_d+ {\mathfrak {B}}_{l, d} + \mathbb {1}_{{\mathbb {N}}}(l) {\mathfrak {B}}_{\max \{l - 1, 0\}, d} \big ). \end{aligned}$$

This and [5, Lemma 3.14] (applied for every $d\in {\mathfrak {D}}$ with $\alpha \curvearrowleft {\mathfrak {R}}_d+ {\mathfrak {B}}_{0,d}$, $\beta \curvearrowleft {\mathfrak {R}}_d$, $M \curvearrowleft M$, $(C_n)_{n \in {\mathbb {N}}_0} \curvearrowleft ({\mathfrak {B}}_{n,d})_{n \in {\mathbb {N}}_0}$ in the notation of [5, Lemma 3.14]) prove that for all $n \in {\mathbb {N}}$, $d\in {\mathfrak {D}}$ it holds that

103 $$\begin{aligned} |A|^{-n} {\mathfrak {C}}_{n, d} = {\mathfrak {B}}_{n, d} \le \bigg ( \frac{{\mathfrak {R}}_d+ {\mathfrak {B}}_{0,d} + {\mathfrak {R}}_d+ {\mathfrak {B}}_{0,d} }{2} \bigg ) (3 M)^n = {\mathfrak {R}}_d(3 M)^n. \end{aligned}$$

Hence, it holds for all $n \in {\mathbb {N}}$, $d\in {\mathfrak {D}}$ that ${\mathfrak {C}}_{n, d} = |A|^n {\mathfrak {B}}_{n, d} \le {\mathfrak {R}}_d(3 |A| M)^n$. This, (99), and [5, Lemma 3.15] (applied for every $d\in {\mathfrak {D}}$ with $m \curvearrowleft 1$, $\alpha \curvearrowleft \alpha$, $\beta \curvearrowleft 3 |A| M$, $\kappa _1 \curvearrowleft \gamma$, $\kappa _2 \curvearrowleft {\mathfrak {R}}_d$,

104 $$\begin{aligned} (c_n)_{n \in {\mathbb {N}}} \curvearrowleft ({\mathfrak {C}}_{n, d})_{n \in {\mathbb {N}}}, \qquad (e_n)_{n \in {\mathbb {N}}} \curvearrowleft \Bigg ( \sup _{x \in {\mathbb {X}}_d} \bigg ( \frac{{\mathbb {E}}\big [ \Vert v_d(x) - V_{n,d}^0(x) \Vert _\infty ^2 \big ]}{|{\mathfrak {w}}_d(x)|^2} \bigg )^{\!\frac{1}{2}} \Bigg )_{n \in {\mathbb {N}}}, \end{aligned}$$

$N \curvearrowleft N$ in the notation of [5, Lemma 3.15]) implies for all $d\in {\mathfrak {D}}$, $\varepsilon \in (0,1]$ that

105 $$\begin{aligned} \sup _{x \in {\mathbb {X}}_d} \bigg ( \frac{{\mathbb {E}}\big [ \Vert v_d(x) \!-\! V_{N_\varepsilon , d}^0(x) \Vert ^2_\infty \big ]}{|{\mathfrak {w}}_d(x)|^2} \bigg )^\frac{1}{2} \!\le \! \varepsilon \quad \text {and} \quad {\mathfrak {C}}_{N_\varepsilon , d} \le 3 |A| M{\mathfrak {R}}_d\max \{ 1, \gamma \}^\beta \varepsilon ^{-\beta }. \end{aligned}$$

Let $$c \in {\mathbb {R}}$$ satisfy

106 $$\begin{aligned} c = \max \{ \beta , 3|A|M \max \{1,\gamma \}^\beta \}. \end{aligned}$$

Note that (105) and (106) ensure for all $d\in {\mathfrak {D}}$, $\varepsilon \in (0,1]$ that ${\mathfrak {C}}_{N_\varepsilon , d} \le c {\mathfrak {R}}_d\varepsilon ^{-c}$. This establishes item (ii). The proof of Theorem 4.1 is thus complete. $\square$

### MLFP Approximations for Bellman Equations of Optimal Control Problems

#### Corollary 4.2

Let $M \in {\mathbb {N}}$, $\kappa \in [0,\infty )$, $\Theta = \bigcup _{n \in {\mathbb {N}}} {\mathbb {Z}}^n$, let *A* be a finite nonempty set, let ${\mathfrak {D}}$ be a nonempty set, let $(\Omega , {\mathcal {F}}, {\mathbb {P}})$ be a probability space, let $\lambda _{d}, \delta _d, {\mathfrak {R}}_d\in [0,\infty )$, $d\in {\mathfrak {D}}$, let $({\mathbb {X}}_d, {\mathcal {X}}_d)$, $d\in {\mathfrak {D}}$, be nonempty measurable spaces, for every $d\in {\mathfrak {D}}$ let ${\mathfrak {w}}_d:{\mathbb {X}}_d\rightarrow (0, \infty )$ be ${\mathcal {X}}_d/{\mathcal {B}}((0,\infty ))$-measurable, for every $d\in {\mathfrak {D}}$ let $g_d:{\mathbb {X}}_d\times A \rightarrow {\mathbb {R}}$ be $({\mathcal {X}}_d\otimes 2^A )/{\mathcal {B}}({\mathbb {R}})$-measurable, for every $d\in {\mathfrak {D}}$ let ${\mathbb {F}}^\theta _d\subseteq {\mathcal {F}}$, $\theta \in \Theta$, be independent sub-sigma-algebras of ${\mathcal {F}}$, for every $d\in {\mathfrak {D}}$ let $X^\theta _d= \big ( X^{\theta , x, a}_d(\omega ) \big )_{x \in {\mathbb {X}}_d, \;a \in A, \; \omega \in \Omega } :{\mathbb {X}}_d\times A \times \Omega \rightarrow {\mathbb {X}}_d$, $\theta \in \Theta$, be i.i.d. random fields which satisfy for all $d\in {\mathfrak {D}}$, $\theta \in \Theta$ that $X^\theta _d$ is $({\mathcal {X}}_d\otimes 2^A \otimes {\mathbb {F}}^\theta _d)/{\mathcal {X}}_d$-measurable, assume $M > \frac{( 1 + (\sup _{d\in {\mathfrak {D}}} \lambda _d\delta _d)(2|A| - 1) )^2}{ (1 - (\sup _{d\in {\mathfrak {D}}}\lambda _d\delta _d))^2 }$, assume for all $d\in {\mathfrak {D}}$, $(x,a) \in {\mathbb {X}}_d\times A$ that $\max _{b \in A} | g_d(x,b) |\le \kappa {\mathfrak {w}}_d(x)$, $\big ( {\mathbb {E}}\big [ \left| {\mathfrak {w}}_d(X_d^{0,x,a})\right| ^2 \big ] \big )^\frac{1}{2} \le \lambda _d{\mathfrak {w}}_d(x)$, $\sup _{u \in {\mathfrak {D}}} \lambda _u \delta _u < 1$, for every $d\in {\mathfrak {D}}$ let ${\mathcal {Q}}^\theta _{n, d} :{\mathbb {X}}_d\times A \times \Omega \rightarrow {\mathbb {R}}$, $\theta \in \Theta$, $n \in {\mathbb {N}}_0$, satisfy for all $n \in {\mathbb {N}}_0$, $(x,a) \in {\mathbb {X}}_d\times A$, $\theta \in \Theta$ that

107 $$\begin{aligned} {\mathcal {Q}}_{n, d}^{\theta }(x,a)&= g_d(x,a) + \sum _{l = 0}^{n-1} \frac{\delta _d}{M^{n-l}} \sum _{i = 1}^{M^{n-l}} \max _{b \in A}\big \{ {\mathcal {Q}}_{l, d}^{(\theta , l, i)}(X_d^{(\theta , l, i), x,a},b) \big \} \nonumber \\&\quad -\mathbb {1}_{{\mathbb {N}}}(l) \max _{b \in A} \big \{ {\mathcal {Q}}_{\max \{ l-1, 0 \}, d}^{(\theta , -l, i)}(X_d^{(\theta , l, i), x,a},b) \big \}, \end{aligned}$$

and let ${\mathfrak {C}}_{n,d} \in [0, \infty )$, $n \in {\mathbb {N}}_0$, $d\in {\mathfrak {D}}$, satisfy for all $n \in {\mathbb {N}}_0$, $d\in {\mathfrak {D}}$ that

108 $$\begin{aligned} {\mathfrak {C}}_{n, d} \le \sum _{l = 0}^{n-1} M^{n-l}\big ( {\mathfrak {R}}_d+ |A|{\mathfrak {C}}_{l, d} + \mathbb {1}_{{\mathbb {N}}}(l)|A| {\mathfrak {C}}_{\max \{l-1, 0\}, d} \big ). \end{aligned}$$

Then the following holds:

(i) For every $d\in {\mathfrak {D}}$ there exists a unique function $Q_d:{\mathbb {X}}_d\times A \rightarrow {\mathbb {R}}$ which is $({\mathcal {X}}_d\otimes 2^A )/ {\mathcal {B}}({\mathbb {R}})$-measurable   and   satisfies   for   all   $(x,a) \in {\mathbb {X}}_d\times A$   that   $\sup _{y \in {\mathbb {X}}_d} \frac{\max \limits _{b \in A}\left| Q_d(y, b) \right| }{{\mathfrak {w}}_d(y)} < \infty$, ${\mathbb {E}}\big [ \big | \max _{b \in A} Q_d(X_d^{0,x,a},b) \big | \big ] < \infty$, and
  109 $$\begin{aligned} Q_d(x,a) = g_d(x,a) + \delta _d{\mathbb {E}}\big [ \max _{b \in A} Q_d(X_d^{0,x,a}, b) \big ]. \end{aligned}$$
(ii) There exist $N :(0, 1] \rightarrow {\mathbb {N}}$ and $c \in {\mathbb {R}}$ such that for all $d\in {\mathfrak {D}}$, $\varepsilon \in (0,1]$ it holds that ${\mathfrak {C}}_{N_\varepsilon , d} \le c {\mathfrak {R}}_d\varepsilon ^{-c}$ and
  110 $$\begin{aligned} \sup _{x \in {\mathbb {X}}_d} \bigg ( \frac{{\mathbb {E}}\big [ \max _{a \in A}| Q_d(x,a) - {\mathcal {Q}}_{N_\varepsilon , d}^0(x, a) |^2 \big ]}{|{\mathfrak {w}}_d(x)|^2} \bigg )^\frac{1}{2} \le \varepsilon . \end{aligned}$$

#### Proof of Corollary 4.2

Note that for every $d\in {\mathfrak {D}}$ it holds that the function ${\mathbb {X}}_d\times {\mathbb {R}}^A \ni (x,r) \mapsto \delta _d\max _{a \in A} \left\{ g_d(x,a) + r(a) \right\} \in {\mathbb {R}}$ is $({\mathcal {X}}_d\otimes ( \bigotimes _{a \in A} {\mathcal {B}}({\mathbb {R}})))/{\mathcal {B}}({\mathbb {R}})$-measurable. Moreover, for all $d\in {\mathfrak {D}}$, $x \in {\mathbb {X}}_d$, $r, s \in {\mathbb {R}}^A$ it holds that

111 $$\begin{aligned}&\big | \delta _d\max _{a \in A} \left\{ g_d(x,a) + r(a) \right\} - \delta _d\max _{a \in A} \left\{ g_d(x,a) + s(a) \right\} \big |\nonumber \\&\quad \le \delta _d\max _{a \in A} \left| r(a) - s(a) \right| . \end{aligned}$$

The assumption that for all $d\in {\mathfrak {D}}$, $(x,a) \in {\mathbb {X}}_d\times A$ it holds that $\max _{b \in A} | g_d(x,b) |\le \kappa {\mathfrak {w}}_d(x)$, $\big ( {\mathbb {E}}\big [ \left| {\mathfrak {w}}_d(X_d^{0,x,a})\right| ^2 \big ] \big )^\frac{1}{2} \le \lambda _d{\mathfrak {w}}_d(x)$, and $\sup _{u \in {\mathfrak {D}}} \lambda _u \delta _u < 1$ yields for all $d\in {\mathfrak {D}}$, $(x,a) \in {\mathbb {X}}_d\times A$ that

112 $$\begin{aligned} \left( {\mathbb {E}}\left[ \left| \delta _d\max _{b \in A} \left\{ g_d(X_d^{0,x,a}, b) \right\} \right| ^2 \right] \right) ^\frac{1}{2}&\le \delta _d\kappa \left( {\mathbb {E}}\left[ \left| {\mathfrak {w}}_d(X_d^{0,x,a}) \right| ^2 \right] \right) ^\frac{1}{2} \nonumber \\&\le \delta _d\kappa \lambda _d{\mathfrak {w}}_d(x) \le \kappa {\mathfrak {w}}_d(x). \end{aligned}$$

For every $d\in {\mathfrak {D}}$ let $R_{n, d}^\theta :{\mathbb {X}}_d\times A \times \Omega \rightarrow {\mathbb {R}}$, $\theta \in \Theta$, $n \in {\mathbb {N}}_0$, satisfy for all $n \in {\mathbb {N}}_0$, $(x,a) \in {\mathbb {X}}_d\times A$, $\theta \in \Theta$ that

113 $$\begin{aligned} R_{n, d}^\theta (x,a)&= \sum _{l = 0}^{n-1} \frac{\delta _d}{M^{n-l}} \sum _{i = 1}^{M^{n - l}} \max _{b \in A} \left\{ g_d(X_d^{(\theta , l, i), x, a}, b) + R_{l, d}^{(\theta , l, i)}\big ( X_d^{(\theta , l, i), x, a}, b \big ) \right\} \nonumber \\&\quad - \mathbb {1}_{{\mathbb {N}}}(l) \max _{b \in A} \left\{ g_d(X_d^{(\theta , l, i), x, a}, b) + R_{\max \{ l-1, 0 \}, d}^{(\theta , -l, i)}\big ( X_d^{(\theta , l, i), x, a}, b \big ) \right\} . \end{aligned}$$

This and Theorem 4.1 (applied with $M \curvearrowleft M$, $\Theta \curvearrowleft \Theta$, $A \curvearrowleft A$, $\kappa \curvearrowleft \kappa$, $(\Omega , {\mathcal {F}}, {\mathbb {P}}) \curvearrowleft (\Omega , {\mathcal {F}}, {\mathbb {P}})$, ${\mathfrak {D}}\curvearrowleft {\mathfrak {D}}$, $(\lambda _d)_{d\in {\mathfrak {D}}} \curvearrowleft (\lambda _d)_{d\in {\mathfrak {D}}}$, $(L_d)_{d\in {\mathfrak {D}}} \curvearrowleft (\delta _d)_{d\in {\mathfrak {D}}}$, $({\mathfrak {R}}_d)_{d\in {\mathfrak {D}}} \curvearrowleft ({\mathfrak {R}}_d)_{d\in {\mathfrak {D}}}$, $({\mathbb {X}}_d, {\mathcal {X}}_d)_{d\in {\mathfrak {D}}} \curvearrowleft ({\mathbb {X}}_d, {\mathcal {X}}_d)_{d\in {\mathfrak {D}}}$, $({\mathfrak {w}}_d)_{d\in {\mathfrak {D}}} \curvearrowleft ({\mathfrak {w}}_d)_{d\in {\mathfrak {D}}}$, $(f_d)_{d\in {\mathfrak {D}}} \curvearrowleft ({\mathbb {X}}_d\times {\mathbb {R}}^A \ni (x,r) \mapsto \delta _d\max _{a \in A} \left\{ g_d(x,a) + r(a) \right\} \in {\mathbb {R}})_{d\in {\mathfrak {D}}}$, $({\mathbb {F}}_d^\theta )_{d\in {\mathfrak {D}}, \; \theta \in \Theta } \curvearrowleft ({\mathbb {F}}_d^\theta )_{d\in {\mathfrak {D}}, \; \theta \in \Theta }$, $(X_d^\theta )_{d\in {\mathfrak {D}}, \; \theta \in \Theta } \curvearrowleft (X_d^\theta )_{d\in {\mathfrak {D}}, \; \theta \in \Theta }$, $({\mathfrak {C}}_{n, d})_{n \in {\mathbb {N}}_0, \; d\in {\mathfrak {D}}} \curvearrowleft ({\mathfrak {C}}_{n, d})_{n \in {\mathbb {N}}_0, \; d\in {\mathfrak {D}}}$, $(V_{n, d}^\theta )_{n \in {\mathbb {N}}_0, \; d\in {\mathfrak {D}}, \; \theta \in \Theta } \curvearrowleft (R_{n, d}^\theta )_{n \in {\mathbb {N}}_0, \; d\in {\mathfrak {D}}, \; \theta \in \Theta }$ in the notation of Theorem 4.1) imply that

(I) for every $d\in {\mathfrak {D}}$ there exists a unique function $R_d:{\mathbb {X}}_d\times A \rightarrow {\mathbb {R}}$ which is $({\mathcal {X}}_d\otimes 2^A) / {\mathcal {B}}({\mathbb {R}})$-measurable and satisfies for all   $(x,a) \in {\mathbb {X}}_d\times A$   that    $\sup _{y \in {\mathbb {X}}_d} \frac{\max _{ b \in A} | R_d(y, b) | }{{\mathfrak {w}}_d(y)} < \infty$, ${\mathbb {E}}\big [ \left| \max _{b \in A} \left\{ g_d(X_d^{0, x, a}, b) + R_d\big ( X_d^{0,x,a},b \big ) \right\} \right| \big ] < \infty$,
  114 $$\begin{aligned} R_d(x,a) = \delta _d{\mathbb {E}}\big [ \max _{b \in A} \left\{ g_d(X_d^{0, x, a}, b) + R_d\big ( X_d^{0,x,a},b \big ) \right\} \big ], \end{aligned}$$
  and
(II) there exist $N :(0, 1] \rightarrow {\mathbb {N}}$ and $c \in {\mathbb {R}}$ such that for all $d\in {\mathfrak {D}}$, $\varepsilon \in (0,1]$ it holds that ${\mathfrak {C}}_{N_\varepsilon , d} \le c {\mathfrak {R}}_d\varepsilon ^{-c}$ and
  115 $$\begin{aligned} \sup _{x \in {\mathbb {X}}_d} \bigg ( \frac{{\mathbb {E}}\big [ \max _{a \in A} | R_d(x,a) - R^0_{N_\varepsilon , d}(x,a)|^2 \big ]}{|{\mathfrak {w}}_d(x)|^2} \bigg )^\frac{1}{2} \le \varepsilon . \end{aligned}$$

For every $d\in {\mathfrak {D}}$ let $Q_d:{\mathbb {X}}_d\times A \rightarrow {\mathbb {R}}$ satisfy for all $(x,a) \in {\mathbb {X}}_d\times A$ that $Q_d(x,a) = g_d(x,a) + R_d(x,a)$. This and item (I) ensure for all $d\in {\mathfrak {D}}$, $(x,a)\in {\mathbb {X}}_d\times A$ that

116 $$\begin{aligned} Q_{d}(x,a)= &   g_{d}(x,a) + R_d(x,a) = g_{d}(x,a) \nonumber \\  &   + \delta _d{\mathbb {E}}\left[ \max _{b\in A} \left\{ g_d(X_d^{0,x,a}, b) + R_d(X_{d}^{0,x,a}, b) \right\} \right] \nonumber \\    &   = g_{d}(x, a) + \delta _{d}{\mathbb {E}}\left[ \max _{b \in A} \left\{ Q_{d}(X_{d}^{0,x,a}, b)\right\} \right] \end{aligned}$$

Moreover,   note   that   the assumption   that   for   all   $d\in {\mathfrak {D}}$,   $x \in {\mathbb {X}}_d$   it   holds   that $\max _{a \in A} \left| g_d(x,a) \right| \le \kappa {\mathfrak {w}}_d(x)$ and item (I) demonstrate that for all $d\in {\mathfrak {D}}$, $(x,a) \in {\mathbb {X}}_d\times A$ it holds that $\sup _{y \in {\mathbb {X}}_d} \frac{\max _{b \in A} \left| Q_d(y,b) \right| }{{\mathfrak {w}}_d(y)} < \infty$ and ${\mathbb {E}}\left[ \left| \max _{b \in A} \left\{ Q_d(X_d^{0,x,a}, b) \right\} \right| \right] < \infty$. Furthermore, for every $d\in {\mathfrak {D}}$ let $S_d:{\mathbb {X}}_d\times A \rightarrow {\mathbb {R}}$ be $({\mathcal {X}}_d\otimes 2^A) / {\mathcal {B}}({\mathbb {R}})$-measurable and satisfy for all $d\in {\mathfrak {D}}$, $(x,a) \in {\mathbb {X}}_d\times A$ that $\sup _{y \in {\mathbb {X}}_d} \frac{\max _{b \in A} \left| S_d(y,b) \right| }{{\mathfrak {w}}_d(y)} < \infty$, ${\mathbb {E}}\left[ \left| \max _{b \in A} \left\{ S_d(X_d^{0,x,a}, b) \right\} \right| \right] < \infty$, and $S_d(x,a) = g_d(x,a) + \delta _d{\mathbb {E}}\left[ \max _{b \in A} \left\{ S_d(X_d^{0,x,a}, b) \right\} \right]$. It holds for all $d\in {\mathfrak {D}}$, $(x,a) \in {\mathbb {X}}_d\times A$ that

117 $$\begin{aligned}&S_d(x,a) - g_d(x,a) \nonumber \\&\quad = \delta _d{\mathbb {E}}\left[ \max _{b \in A} \left\{ S_d(X_d^{0,x,a}, b) \right\} \right] \nonumber \\&\quad = \delta _d{\mathbb {E}}\left[ \max _{b \in A} \left\{ g_d(X_d^{0,x,a}, b) + S_d(X_d^{0,x,a}, b) - g_d(X_d^{0,x,a}, b) \right\} \right] . \end{aligned}$$

Item (I) implies that for all $d\in {\mathfrak {D}}$ it holds that $S_d- g_d= R_d$. This establishes for all $d\in {\mathfrak {D}}$ that $S_d= Q_d$. This proves item (i). Note that induction and (113) demonstrate that for all $d\in {\mathfrak {D}}$, $n \in {\mathbb {N}}_0$, $(x,a) \in {\mathbb {X}}_d\times A$, $\theta \in \Theta$ it holds that ${\mathcal {Q}}_{n, d}^\theta (x, a) = g_d(x, a) + R_{n, d}^\theta (x,a)$. Combining this and item (II) implies for all $d\in {\mathfrak {D}}$, $\varepsilon \in (0,1]$ that

118 $$\begin{aligned}&\sup _{x \in {\mathbb {X}}_d} \bigg ( \tfrac{{\mathbb {E}}\big [ \max _{a \in A} | Q_d(x,a) - {\mathcal {Q}}_{N_\varepsilon , d}^0(x, a) |^2 \big ]}{|{\mathfrak {w}}_d(x)|^2} \bigg )^\frac{1}{2} \nonumber \\&= \sup _{x \in {\mathbb {X}}_d} \bigg ( \tfrac{{\mathbb {E}}\big [ \max _{a \in A}| R_d(x,a) - R_{N_\varepsilon , d}^0(x, a) |^2 \big ]}{|{\mathfrak {w}}_d(x)|^2} \bigg )^\frac{1}{2} \le \varepsilon . \end{aligned}$$

This proves item (ii). The proof of Corollary 4.2 is thus complete. $\square$

### MLFP Approximations for Bellman Equations of Optimal Stopping Problems

#### Corollary 4.3

Let $M \in {\mathbb {N}}$, let $\Theta = \bigcup _{n \in {\mathbb {N}}} {\mathbb {Z}}^n$, let $(\Omega , {\mathcal {F}}, {\mathbb {P}})$ be a probability space, let ${\mathfrak {D}}$ be a nonempty set, let $\delta _d, {\mathfrak {R}}_d\in [0, \infty )$, $d\in {\mathfrak {D}}$, let $({\mathbb {X}}_d, {\mathcal {X}}_d)$, $d\in {\mathfrak {D}}$, be nonempty Borel spaces, for every $d\in {\mathfrak {D}}$ let $g_d:{\mathbb {X}}_d\rightarrow {\mathbb {R}}$ and $G_d:{\mathbb {X}}_d\rightarrow {\mathbb {R}}$ be ${\mathcal {X}}_d/ {\mathcal {B}}({\mathbb {R}})$-measurable, for every $d\in {\mathfrak {D}}$ let ${\mathbb {F}}_d^\theta \subseteq {\mathcal {F}}$, $\theta \in \Theta$, be independent sub-sigma-algebras of ${\mathcal {F}}$, for every $d\in {\mathfrak {D}}$ let $X_d^\theta = (X_d^{\theta , x}(\omega ))_{x \in {\mathbb {X}}_d,\; \omega \in \Omega } :{\mathbb {X}}_d\times \Omega \rightarrow {\mathbb {X}}_d$, $\theta \in \Theta$, be i.i.d. random fields which satisfy for all $d\in {\mathfrak {D}}$, $\theta \in \Theta$ that $X_d^\theta$ is $({\mathcal {X}}_d\otimes {\mathbb {F}}_d^\theta ) / {\mathcal {X}}_d$-measurable, assume $\sup _{d\in {\mathfrak {D}}} \delta _d< 1$, assume $M > \frac{(1 + 3 (\sup _{d\in {\mathfrak {D}}} \delta _d))^2}{(1 - (\sup _{d\in {\mathfrak {D}}} \delta _d))^2}$, assume $\sup _{d\in {\mathfrak {D}}} \left( \sup _{x \in {\mathbb {X}}_d} |g_d(x)| + |G_d(x)| \right) < \infty$, for every $d\in {\mathfrak {D}}$ let ${\mathcal {Q}}_{n, d}^\theta :{\mathbb {X}}_d\times \Omega \rightarrow {\mathbb {R}}$, $n \in {\mathbb {N}}_0$, $\theta \in \Theta$, satisfy for all $n \in {\mathbb {N}}_0$, $x \in {\mathbb {X}}_d$, $\theta \in \Theta$ that

119 $$\begin{aligned} {\mathcal {Q}}_{n, d}^\theta (x)&= g_d(x) + \sum _{l = 0}^{n-1} \frac{\delta _d}{M^{n-l}} \sum _{i = 1}^{M^{n-l}} \max \left\{ G_d\big ( X_d^{(\theta , l, i), x} \big ), {\mathcal {Q}}_{l, d}^{(\theta , l, i)}\big (X_d^{(\theta , l, i), x} \big ) \right\} \nonumber \\&\quad - \mathbb {1}_{{\mathbb {N}}}(l) \max \left\{ G_d\big ( X_d^{(\theta , l, i), x} \big ), {\mathcal {Q}}_{\max \{ l-1, 0 \}, d}^{(\theta , -l, i)} \big ( X_d^{(\theta , l, i),x} \big ) \right\} \end{aligned}$$

and let ${\mathfrak {C}}_{n, d} \in [0, \infty )$, $n \in {\mathbb {N}}_0$, $d\in {\mathfrak {D}}$, satisfy for all $n \in {\mathbb {N}}_0$, $d\in {\mathfrak {D}}$ that

120 $$\begin{aligned} {\mathfrak {C}}_{n, d} \le \sum _{l = 0}^{n-1} M^{n-l} \left( {\mathfrak {R}}_d+ 2{\mathfrak {C}}_{l, d} + \mathbb {1}_{\mathbb {N}}(l) 2{\mathfrak {C}}_{\max \{ l-1, 0 \}, d} \right) . \end{aligned}$$

Then the following holds:

(i) For every $d\in {\mathfrak {D}}$ there exists a unique function $Q_d:{\mathbb {X}}_d\rightarrow {\mathbb {R}}$ which is ${\mathcal {X}}_d/{\mathcal {B}}({\mathbb {R}})$-measurable and satisfies for all $x \in {\mathbb {X}}_d$ that $\sup _{y \in {\mathbb {X}}_d} |Q_d(y)| < \infty$, and
  121 $$\begin{aligned} Q_d(x) = g_d(x) + \delta _d{\mathbb {E}}\left[ \max \left\{ G_d(X_d^{0,x}), Q_d(X_d^{0,x}) \right\} \right] . \end{aligned}$$
(ii) There exist $N :(0,1] \rightarrow {\mathbb {N}}$ and $c\in {\mathbb {R}}$ such that for all $d\in {\mathfrak {D}}$, $\varepsilon \in (0,1]$ it holds that ${\mathfrak {C}}_{N_\varepsilon , d} \le c {\mathfrak {R}}_d\varepsilon ^{-c}$ and
  122 $$\begin{aligned} \sup _{x \in {\mathbb {X}}_d} \left( {\mathbb {E}}\left[ \big | Q_d(x) - {\mathcal {Q}}_{N_\varepsilon , d}^0(x) \big |^2 \right] \right) ^\frac{1}{2} \le \varepsilon . \end{aligned}$$

#### Proof of Corollary 4.3

Let ${\mathbb {Y}}_d$, $d\in {\mathfrak {D}}$, be nonempty sets which satisfy that there exists $\Upsilon \in \bigcap _{d\in {\mathfrak {D}}} {\mathbb {Y}}_d$, and that for all $d\in {\mathfrak {D}}$ it holds that ${\mathbb {Y}}_d\backslash \{ \Upsilon \} = {\mathbb {X}}_d$, for every $d\in {\mathfrak {D}}$ let ${\mathcal {Y}}_d= \sigma _{{\mathbb {Y}}_d}({\mathcal {X}}_d)$, for every $d\in {\mathfrak {D}}$, $\theta \in \Theta$ let $Y^\theta _d= (Y_d^{\theta , y, a}(\omega ))_{y \in {\mathbb {Y}}_d, \; a \in \{ 0,1 \},\; \omega \in \Omega } :{\mathbb {Y}}_d\times \{0,1\} \times \Omega \rightarrow {\mathbb {Y}}_d$ satisfy for all $d\in {\mathfrak {D}}$, $\theta \in \Theta$, $(y, a) \in {\mathbb {Y}}_d\times \{0,1\}$, $\omega \in \Omega$ that

123 $$\begin{aligned} Y_d^{\theta , y, a}(\omega )&= {\left\{ \begin{array}{ll} \Upsilon &  :(y = \Upsilon ) \vee (a = 0),\\ X_d^{\theta , y}(\omega ) &  :(y \ne \Upsilon ) \wedge (a = 1). \end{array}\right. } \end{aligned}$$

Note that for all $d\in {\mathfrak {D}}$ it holds that $Y_d^\theta$, $\theta \in \Theta$, are i.i.d. random fields and that for every $d\in {\mathfrak {D}}$, $\theta \in \Theta$ it holds that $Y_d^\theta$ is $( {\mathcal {Y}}_d\otimes 2^{\{0,1\}} \otimes {\mathbb {F}}_d^\theta ) / {\mathcal {Y}}_d$-measurable. For every $d\in {\mathfrak {D}}$ let $h_d:{\mathbb {Y}}_d\times \{ 0,1\} \rightarrow {\mathbb {R}}$ satisfy for all $d\in {\mathfrak {D}}$, $(y, a) \in {\mathbb {Y}}_d\times \{ 0,1 \}$ that

124 $$\begin{aligned} h_d(y, a)&= {\left\{ \begin{array}{ll} 0 &  :y = \Upsilon ,\\ g_d(y) &  :y \ne \Upsilon , \; a = 1,\\ G_d(y) &  :y \ne \Upsilon , \; a = 0. \end{array}\right. } \end{aligned}$$

Note that for all $d\in {\mathfrak {D}}$ it holds that $h_d$ is $({\mathcal {Y}}_d\otimes 2^{\{0,1 \}})/{\mathcal {B}}({\mathbb {R}})$-measurable. Moreover, for all $d\in {\mathfrak {D}}$, $(y, a) \in {\mathbb {X}}_d\times \{ 0,1 \}$ it holds that

125 $$\begin{aligned} | h_d(y,a) | \le \max \{ |g_d(y)|, |G_d(y)| \} \le \sup _{u \in {\mathfrak {D}}} \left( \sup _{x \in {\mathbb {X}}_d} |g_u(x)| + |G_u(x)| \right) . \end{aligned}$$

For every $d\in {\mathfrak {D}}$ let $q_{n, d}^\theta :{\mathbb {Y}}_d\times \{ 0,1\} \times \Omega \rightarrow {\mathbb {Y}}_d$, $\theta \in \Theta$, $n \in {\mathbb {N}}_0$, satisfy for all $n \in {\mathbb {N}}_0$, $\theta \in \Theta$, $(y, a) \in {\mathbb {Y}}_d\times \{ 0,1\}$ that

126 $$\begin{aligned} q_{n, d}^\theta (y, a)&= h_d(y, a) + \sum _{l = 0}^{n-1} \frac{\delta _d}{M^{n-l}} \sum _{i = 1}^{M^{n-l}} \max _{b \in \{ 0,1 \} }\big \{ q_{l, d}^{(\theta , l, i)}(Y_d^{(\theta , l, i), y,a}, b) \big \} \nonumber \\&\quad -\mathbb {1}_{{\mathbb {N}}}(l) \max _{b \in \{ 0,1 \} } \big \{ q_{\max \{ l-1, 0 \}, d}^{(\theta , -l, i)}(Y_d^{(\theta , l, i), y,a},b) \big \}. \end{aligned}$$

Corollary 4.2 (applied with $A \curvearrowleft \{ 0,1\}$, $\kappa \curvearrowleft \sup _{d\in {\mathfrak {D}}} ( \sup _{x \in {\mathbb {X}}_d} |g_d(x)| + |G_d(x)| )$, $g_d\curvearrowleft h_d$, $({\mathbb {X}}_d, {\mathcal {X}}_d) \curvearrowleft ({\mathbb {Y}}_d, {\mathcal {Y}}_d)$, ${\mathcal {Q}}_{n, d}^\theta \curvearrowleft q_{n,d}^\theta$ for $d\in {\mathfrak {D}}$ in the notation of Corollary 4.2) establishes the following:

(I) For every $d\in {\mathfrak {D}}$ there exists a unique function $q_d:{\mathbb {Y}}_d\times \{ 0,1 \} \rightarrow {\mathbb {R}}$ which is $({\mathcal {Y}}_d\otimes 2^{\{ 0,1\}}) / {\mathcal {B}}({\mathbb {R}})$-measurable and satisfies for all $(y, a) \in {\mathbb {Y}}_d\times \{ 0, 1\}$ that $\sup _{(z, b) \in {\mathbb {Y}}_d\times \{ 0,1 \}} |q_d(z,b)| < \infty$ and
  127 $$\begin{aligned} q_d(y, a) = h_d(y,a) + \delta _d{\mathbb {E}}\Big [ \max _{b \in \{ 0,1 \}} q_d(Y_d^{0, y, a}, b) \Big ]. \end{aligned}$$
(II) There exist $N :(0, 1] \rightarrow {\mathbb {N}}$ and $c \in {\mathbb {R}}$ such that for all $d\in {\mathfrak {D}}$, $\varepsilon \in (0,1]$ it holds that ${\mathfrak {C}}_{N_\varepsilon , d} \le c {\mathfrak {R}}_d\varepsilon ^c$ and
  128 $$\begin{aligned} \sup _{y \in {\mathbb {Y}}_d} \left( {\mathbb {E}}\Big [ \max _{a \in \{ 0,1\} } | q_d(y,a) - q_{N_\varepsilon , d}^0(y, a) |^2 \Big ] \right) ^\frac{1}{2} \le \varepsilon . \end{aligned}$$

Note that for all $d\in {\mathfrak {D}}$, $(y, a) \in {\mathbb {Y}}_d\backslash \{ \Upsilon \} \times \{ 0, 1\}$ it holds that $q_d(\Upsilon , a) = 0$ and $q_d(y, 0) = G_d(y)$. Moreover, for all $d\in {\mathfrak {D}}$, $y \in {\mathbb {Y}}_d\backslash \{ \Upsilon \}$ it holds that

129 $$\begin{aligned} q_d(y, 1)&= h_d(y,1) + \delta _d{\mathbb {E}}\big [ \max \big \{ q_d(Y_d^{0,y,1}, 0), q_d(Y_d^{0,y,1}, 1) \big \} \big ] \nonumber \\&= g_d(y) + \delta _d{\mathbb {E}}\left[ \max \left\{ G_d(X_d^{0, y}), q_d(X_d^{0,y}, 1) \right\} \right] . \end{aligned}$$

This and item (I) demonstrate for all $d\in {\mathfrak {D}}$ that there exists a unique function $Q_d:{\mathbb {X}}_d\rightarrow {\mathbb {R}}$ which is ${\mathcal {X}}_d/{\mathcal {B}}({\mathbb {R}})$-measurable and satisfies for all $x \in {\mathbb {X}}_d$ that $\sup _{y \in {\mathbb {X}}_d} |Q_d(y)| < \infty$ and

130 $$\begin{aligned} Q_d(x) = g_d(x) + \delta _d{\mathbb {E}}\left[ \max \left\{ G_d(X_d^{0,x}), Q_d(X_d^{0,x}) \right\} \right] . \end{aligned}$$

This proves item (i). Furthermore, note that for all $d\in {\mathfrak {D}}$, $\theta \in \Theta$, $(y, a) \in {\mathbb {Y}}_d\backslash \{\Upsilon \} \times \{0,1\}$ it holds ${\mathbb {P}}$-a.s. that $q_{0, d}^\theta (\Upsilon , a) = 0$, $q_{0, d}^\theta (y,0) = G_d(y)$, and $q_{0,d}^\theta (y, 1) = g_d(y)$. This and induction yield that for all $d\in {\mathfrak {D}}$, $\theta \in \Theta$, $n \in {\mathbb {N}}_0$, $(y, a) \in {\mathbb {Y}}_d\backslash \{ \Upsilon \} \times \{0,1\}$ it holds ${\mathbb {P}}$-a.s. that $q_{n, d}^\theta (\Upsilon , a) = 0$ and $q_{n, d}^\theta (y,0) = G_d(y)$. Combining this, (123), (124), and (126) demonstrates that for all $d\in {\mathfrak {D}}$, $\theta \in \Theta$, $n \in {\mathbb {N}}_0$, $y \in {\mathbb {Y}}_d\backslash \{ \Upsilon \} = {\mathbb {X}}_d$ it holds ${\mathbb {P}}$-a.s. that

131 $$\begin{aligned} q_{n, d}^\theta (y, 1)&= h_d(y,1) + \sum _{l = 0}^{n-1} \frac{\delta _d}{M^{n-l}} \sum _{i = 1}^{M^{n-l}} \max _{b \in \{ 0,1 \} } \left\{ q_{l, d}^{(\theta , l, i)}(Y_d^{(\theta , l, i), y, 1}, b) \right\} \nonumber \\&\quad -\mathbb {1}_{{\mathbb {N}}}(l) \max _{b \in \{ 0,1 \} } \left\{ q_{\max \{ l-1, 0 \}, d}^{(\theta , -l, i)}(Y_d^{(\theta , l, i), y, 1}, b) \right\} \nonumber \\&= g_d(y) + \sum _{l = 0}^{n-1} \frac{\delta _d}{M^{n-l}} \sum _{i = 1}^{M^{n-l}} \max \left\{ G_d(X_d^{(\theta , l, i), y}), q_{l, d}^{(\theta , l, i)}(X_d^{(\theta , l, i),y}, 1) \right\} \nonumber \\&\quad - \mathbb {1}_{{\mathbb {N}}}(l) \max \left\{ G_d(X_d^{(\theta , l, i),y}), q_{ \max \{ l-1, 0 \} , d}^{(\theta , -l, i)}(X_d^{(\theta , l, i),y}, 1) \right\} . \end{aligned}$$

This and (119) yield that for all $d\in {\mathfrak {D}}$, $\theta \in \Theta$, $n \in {\mathbb {N}}_0$, $y \in {\mathbb {Y}}_d\backslash \{ \Upsilon \} = {\mathbb {X}}_d$ it holds ${\mathbb {P}}$-a.s. that

132 $$\begin{aligned} q_{n, d}^\theta (y, 1) = Q_{n,d}^\theta (y). \end{aligned}$$

Combining this and (128) implies that for all $d\in {\mathfrak {D}}$, $\varepsilon \in (0,1]$ it holds that

133 $$\begin{aligned}&\sup _{x \in {\mathbb {X}}_d} \left( {\mathbb {E}}\Big [ \big | Q_d(x) - {\mathcal {Q}}_{N_\varepsilon , d}^0(x) \big |^2 \Big ] \right) ^\frac{1}{2} \le \sup _{y \in {\mathbb {Y}}_d} \left( {\mathbb {E}}\Big [ \max _{a \in \{ 0,1\}} \big | q_d(y,a) - q_{N_\varepsilon , d}^0(y,a) \big |^2 \Big ] \right) ^\frac{1}{2}\nonumber \\&\quad \le \varepsilon . \end{aligned}$$

This establishes item (ii). The proof of Corollary 4.3 is thus complete. $\square$

## Footnotes

1 Note that for every finite set *B* it holds that the number of elements of the power set $$\text {2}^{B}$$ of *B* satisfies $$|\text {2}^{B}| = 2^{|B|}$$. Moreover, note that for all finite sets *B* and *C* it holds that the number of elements of the set of all functions $$B^C$$ from *C* to *B* satisfies $$ |B^C| = |B|^{|C|}$$.
